# Janus All‐*Cis* 2,3,4,5,6‐Pentafluorocyclohexyl Building Blocks Applied to Medicinal Chemistry and Bioactives Discovery Chemistry

**DOI:** 10.1002/chem.202102819

**Published:** 2021-10-06

**Authors:** Joshua L. Clark, Rifahath M. Neyyappadath, Cihang Yu, Alexandra M. Z. Slawin, David B. Cordes, David O'Hagan

**Affiliations:** ^1^ School of Chemistry University of St Andrews North Haugh, St Andrews, Fife KY16 9ST UK

**Keywords:** aryl hydrogenation, fluorocyclohexanes, Janus motif, organic chemistry, organofluorine chemistry

## Abstract

Monoalkylated derivatives of the unusually polar all‐*cis* 2,3,4,5,6‐ pentafluorocyclohexyl (Janus face) motif are prepared starting from an aryl hydrogenation of 2,3,4,5,6‐ pentafluorophenylacetate methyl ester **15**. The method used Zeng's Rh(CAAC) carbene catalyst **4** in the hydrogenation following the protocol developed by Glorius. The resultant Janus pentafluorocyclohexylacetate methyl ester **16** was converted to the corresponding alcohol **18**, aldehyde **13**, bromide **29** and azide **14** through functional group manipulations, and some of these building blocks were used in Ugi‐multicomponent and Cu‐catalysed click reactions. NBoc protected pentafluoroarylphenylalanine methyl ester **35** was also subject to an aryl hydrogenation, and then deprotection to generate the Janus face β‐pentafluorocyclohexyl‐alanine amino acid **15**, which was incorporated into representative members of an emerging class of candidate antiviral compounds. Log P measurements demonstrate that the all‐*cis* 2,3,4,5,6‐pentafluorocyclohexyl ring system is more polar than a phenyl ring. In overview the paper introduces new building blocks containing this Janus ring and demonstrates their progression to molecules typically used in bioactives discovery programmes.

## Graphical abstract

**Janus all‐*cis*** 2,3,4,5,6‐pentafluorocyclohexyl building blocks have been incorporated into medicinal chemistry type products. These cyclohexanes have polarized faces and represent a unique motif for medicinal chemistry. The paper introduces a range of new accessible building blocks which can be elaborated into a diversity of drug type structures demonstrating their wider utility for exploration in bioactives discovery chemistry.

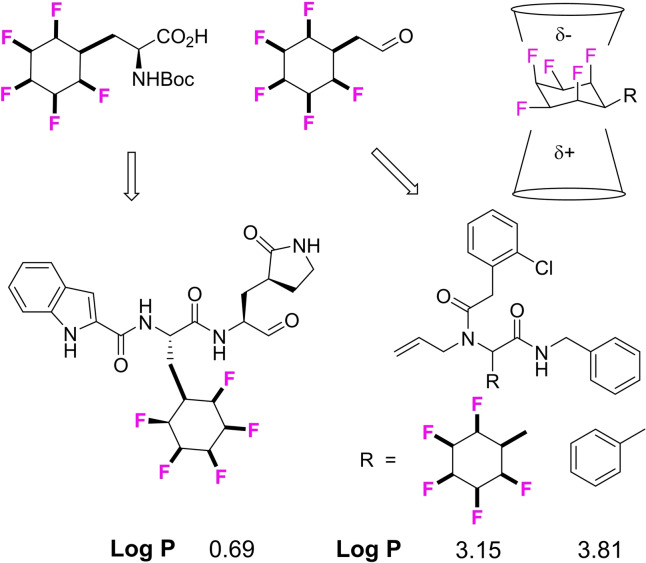

## Introduction

Over the last two decades or so organofluorine chemistry has experienced a ‘Renaissance’, driven by an interest in addressing the synthesis and properties of products carrying isolated −F or −CF_3_ groups, particularly when attached to sp^3^ carbons.^[1]^ This is distinct from an immediately previous era which was defined by the perfluorocarbons and fluoropolymers industries or within fine chemicals, with a focus on aryl −F and −CF_3_ compounds.^[2]^ The prospects of novel bioactives (med‐chem and agrochemicals) resulting from stereogenic fluorine has driven this more recent agenda, and innovation has been stoked by the intense international effort and extraordinary growth in new methods development and catalysis, which defines modern organic chemistry.^[3]^ Access to this next generation of selectively fluorinated alkyls has led to a re‐evaluation of dogmas such as ‘fluorine increases lipophilicity’. In many cases −F or −CF_3_ substituted alkyls become more polar (less lipophilic) than the hydrocarbon and such fluorine effects are being explored in detail.^[4]^

**Figure 1:**
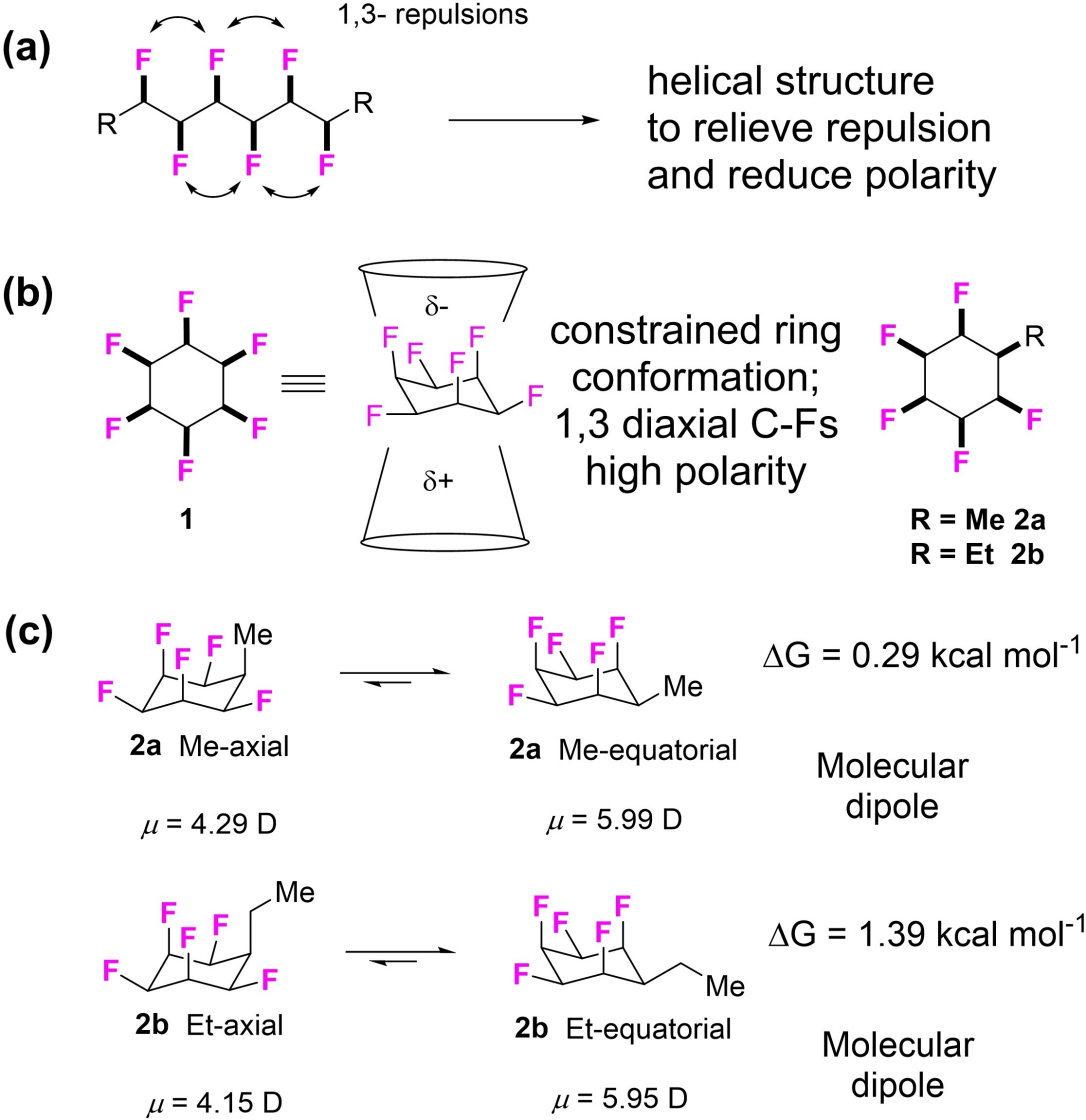
(a) Acyclic multivicinal fluoroalkane chain, adopts a helical conformation. (b) Janus face aspect imparts high polarity to cyclohexane **1** and derivatives **2** with all‐*cis* fluorines. (c) Monoalkylated derivatives **2** favour the more polar triaxial C−F conformation with an equatorial alkyl group.^[10]^

We have had a long interest in selective fluorination of alkyl chains, and particularly in a class of molecules which we have termed ‘multi vicinal fluoroalkanes’ (Figure 1a) that are defined by placing a fluorine on each carbon along a chain and with a defined stereochemistry, to limit isomer mixtures.^[5]^ The programme progressed from linear to cyclic alkanes and most recently we have been exploring the synthesis and properties of cyclohexanes with multiple vicinal fluorines attached around the ring with the all‐*cis* stereochemistry.[^6^, ^7^, ^8^, ^9^, ^10^] The parent structure, all‐*cis* 1,2,3,4,5,6‐hexafluorocyclohexane **1** adopts a chair conformation resulting in triaxial C−F bonds, and this arrangement imparts a very high polarity to the ring system as demonstrated by the extraordinary polar properties of **1** (Figure 1b). Cyclohexane **1** has a melting point of 208 °C and a dipole moment of 6.5 D which are particularly high values for an aliphatic.^[6]^ This has been termed a ‘Janus’ ring system as the fluorine and hydrogen faces, face in opposite directions and are responsible for imparting this polarity.^[11]^

Most recently^[10]^ we have explored monoalkylated structures of all‐*cis* pentafluorocyclohexane represented by **2** **a** and **2** **b**, where R is either Me or Et respectively. These systems adopt a conformation, both in gas phase calculations and in solid state structures, where the R substituent lies equatorial and there are three triaxial C−F bonds, rather than the R group axial and with two diaxial C−F bonds. Therefore against expectation, the preferred conformation is the more polar for mono alkylated ring systems. There is a recent and developing interest in applying this motif to solution state coordination of anions^[12]^ and for controlled supramolecular assembly of polymers^[13]^ due to the self association of the polar rings, however these derivatives have yet to be explored in a medicinal chemistry context. In that context the Janus all‐*cis* pentafluorocyclohexyl ring system is anticipated to have unique properties not represented by any other substituent in organic chemistry as it has an electronegative fluorine face and an electropositive hydrogen face, and both faces have the potential to interact^,[8][12]^ with polar amino acid side chains of opposite electrostatic polarity in protein receptors or enzymes.

**Figure 2:**
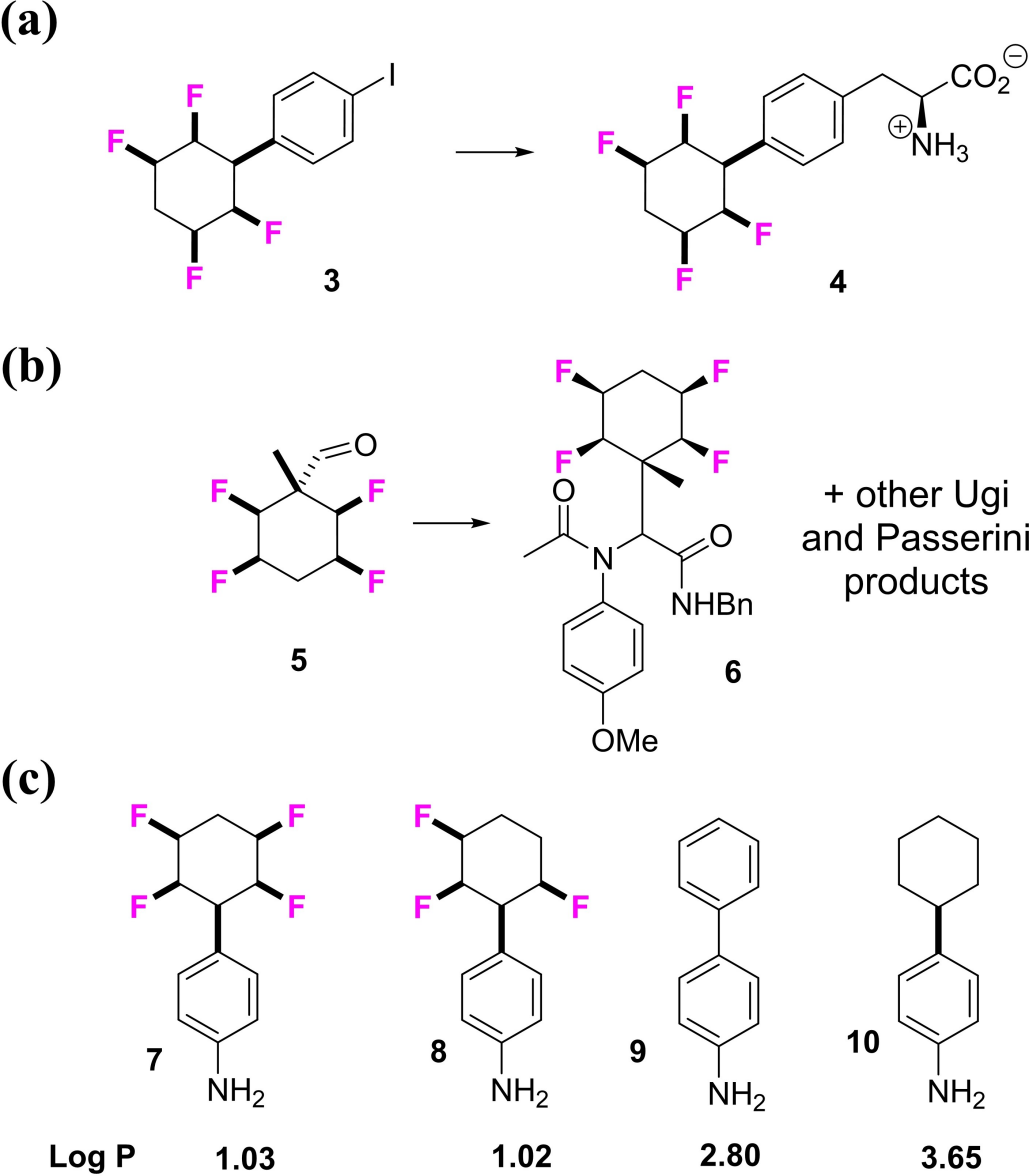
Previous disclosures on all‐*cis* 2,3,5,6 ‐ tetrafluorocyclohexane motifs. (a) An amino acid derivative **4**;^[14d]^ (b) aldehyde **5** as an Ugi component. (c). Log P trends across anilines **7–10** indicate increased hydrophilicity with increased fluorination.^[16]^

**Figure 3:**
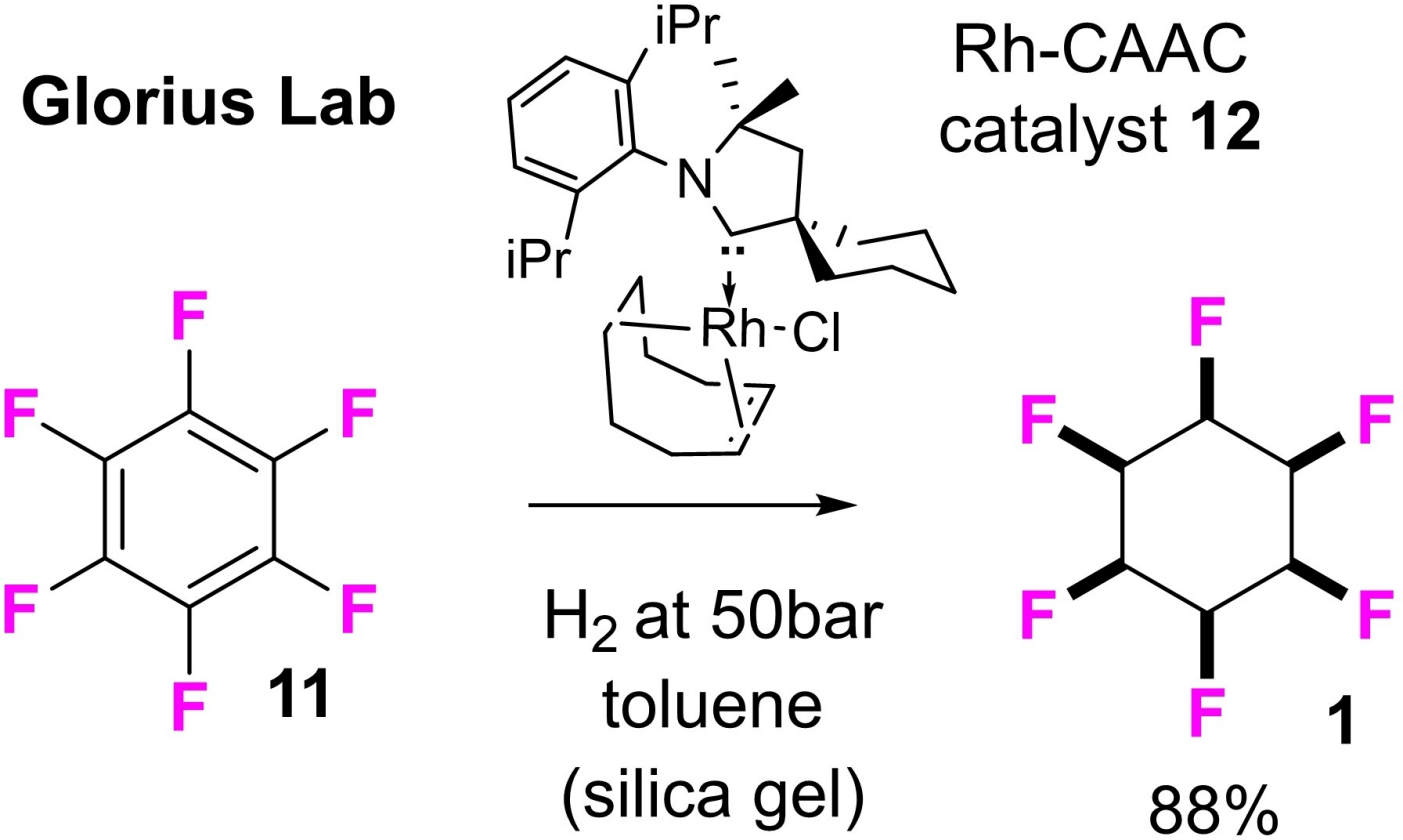
Catalyst and *cis*‐selective hydrogenation of hexafluorobenzene **11** to give **1** as reported by Glorius.[^17^, ^18^]

Previously^[14]^ we have reported on the preparation of building blocks for the incorporation of all‐*cis* 2,3,5,6 ‐ tetrafluorocyclohexane motifs into molecular architectures appropriate to bioactives discovery with illustrative examples shown in Figure 2. For example amino acid **4** could be prepared^[14d]^ in various protected forms and the peptoid product **6** ^[14c]^ represents a range of products which were generated through Ugi and Passerini multicomponent reactions.^[15]^ With such compounds in hand it became apparent that the fluorine of the 2,3,5,6 ‐ tetrafluorocyclohexane ring system rendered these compounds significantly more hydrophilic relative to their aromatic and non‐fluorinated cyclohexyl analogues.^[16]^ This is a striking feature of the ring system as fluorine is generally considered to impart lipophilicity, however in these ring systems the fluorine imparts hydrophilicity, as determined by comparative Log P measurements, illustrated for the anilines **7**–**10** in Figure 2. This is due to the polar nature of the fluorocyclohexane ring with water associating to the protic face of the Janus rings through hydrogen bonding.^[16]^ In this paper we now explore the all‐*cis* 1,2,3,4,5‐pentafluorocyclohexane ring system in a similar context. Until recently this arrangement of a substituted cyclohexane and with five fluorine around the ring was not synthetically accessible, however the Glorius lab have developed^[17]^ a catalytic fluoroaryl hydrogenation which we have used here to access this class of compounds. The prototype example shown in Figure 3 demonstrated the efficient hydrogenation of hexafluorobenzene **11** using Zeng's CAAC catalyst **12^[^** ^18]^ to generate hexafluorocyclohexane **1**, and the method was extended to prepare a range of all‐*cis* fluorocyclohexanes and also with different functionalities.^[17]^

**Figure 4:**
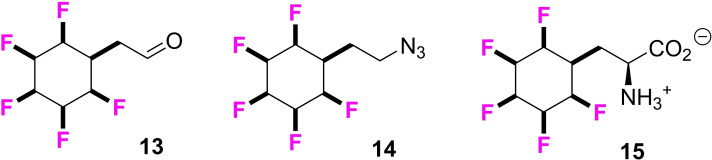
Key building blocks **13**–**15** carrying the all‐*cis* 2,3,4,5,6‐pentafluorocyclohexyl motif used in this study.

In this paper we report the preparation of a range of building blocks incorporating the all‐*cis* 2,3,4,5,6‐pentafluoro‐ cyclohexypentafluoro cyclohexyl motif but with a particular focus on elaborations of aldehyde **13**, azide **14** and amino acid **15** as illustrated in Figure 4.

The utility of aldehyde **13** is explored in Ugi multicomponent reactions,^[15]^ the azide in Cu‐catalysed ‘click’ reactions^[19]^ and the amino acid is incorporated into known coronavirus inhibitor analogues.[^20^, ^21^] It emerges as a general phenomenon that the replacement of cyclohexyl or aryl ring by the all‐*cis* 2,3,4,5,6‐pentafluorocyclohexyl moiety results in a significant reduction in Log P values, which suggests that these motifs would be quite acceptable starting points, for example as fragments for bioactives and drug discovery programmes.

## Results and Discussion

**Scheme 1:**
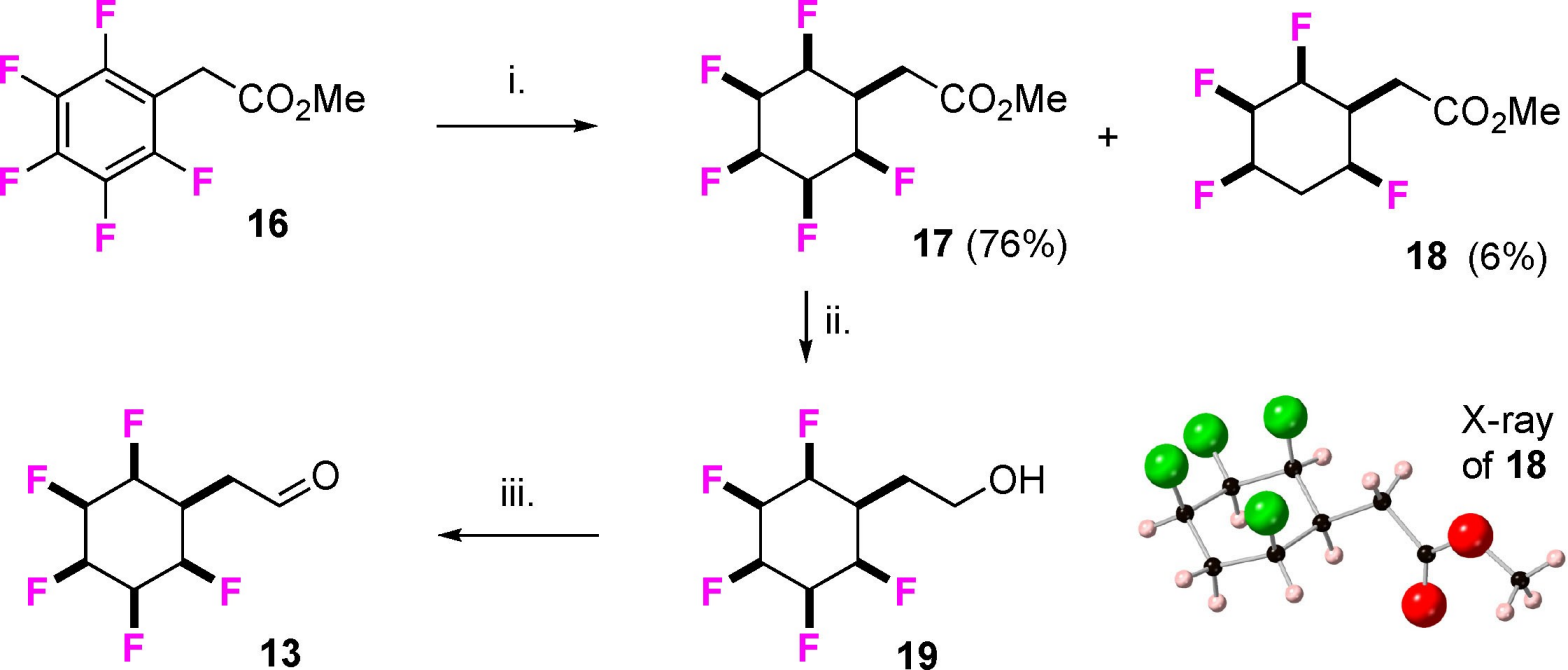
i. **12** (1 mol%), H_2_ (50 bar), hexane, 4 Å MS, r.t., 16h; ii. DIBAL, THF, 0 °C to r.t, 16h, 83%; iii. Dess‐Martin periodinane, THF, r.t, 45min, 85%. X‐ray structure of **17**.

The pentafluoroaryl phenylacetate ester **16** is readily available and offered a convenient starting point for building block development. Aryl hydrogenation using the Zeng catalyst **12** ^[17]^ and following Glorius protocols^[16]^ resulted in an efficient conversion to the corresponding cyclohexyl ester **17** as the major product with the 5‐defluorinated co‐product **18** as a minor product (**17** : **18**, 11 : 1). The regiospecific nature of the defluorination was established by X‐ray crystallography of **18** (Scheme 1). This minor co‐product could be removed by chromatography and **17** was progressed to further transformations. Reduction of the ester moiety of **17** to aldehyde **13** was carried out in a two‐step protocol as a one‐step controlled reduction with DIBAL did not offer any yield advantage. Accordingly, ester **17** was reduced to alcohol **19** with DIBAL and this product was conveniently oxidised to aldehyde **13** after exposure to Dess‐Martin periodinane.^[22]^

**Figure 5:**
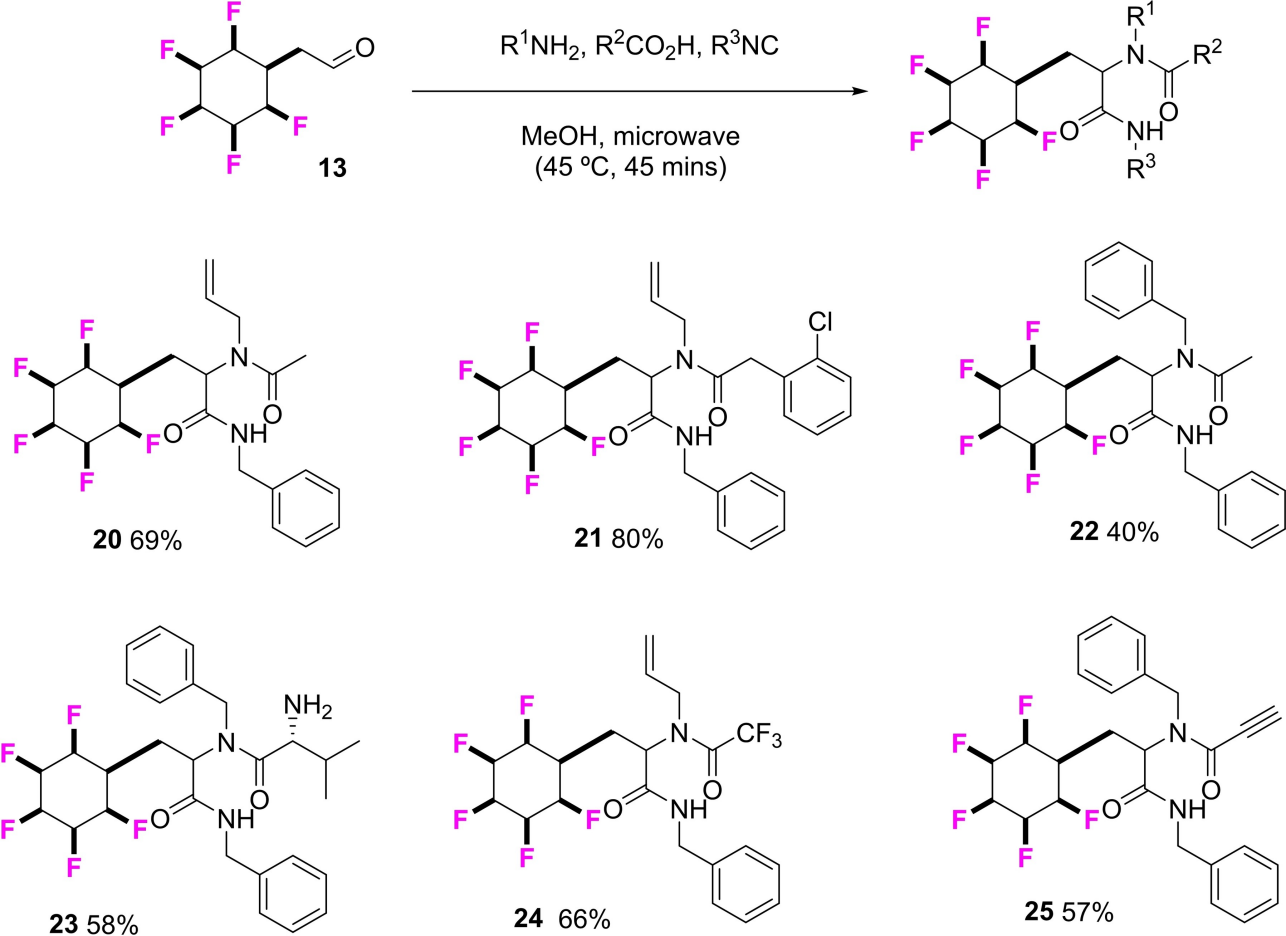
General Ugi multicomponent procedure with aldehyde **13** showing isolated yields and product structures **20**–**25**.

^1^H NMR analysis revealed that aldehyde **13** readily forms a hemiacetal with [^2^H_4_]‐methanol suggesting a significant electron withdrawing effect of the pentafluorocyclohexane ring system. None‐ the‐less it was a good substrate in a number of 4‐component Ugi reactions, of which the products **20**–**25** are summarised in Figure 5.

**Figure 6:**
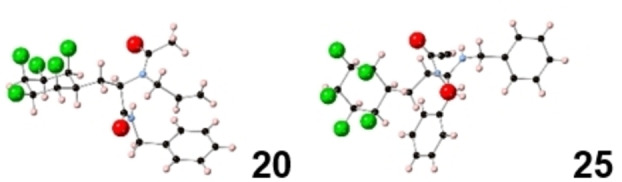
X‐ray crystal structures of Ugi multicomponent products **20** and **25**.

**Figure 7:**
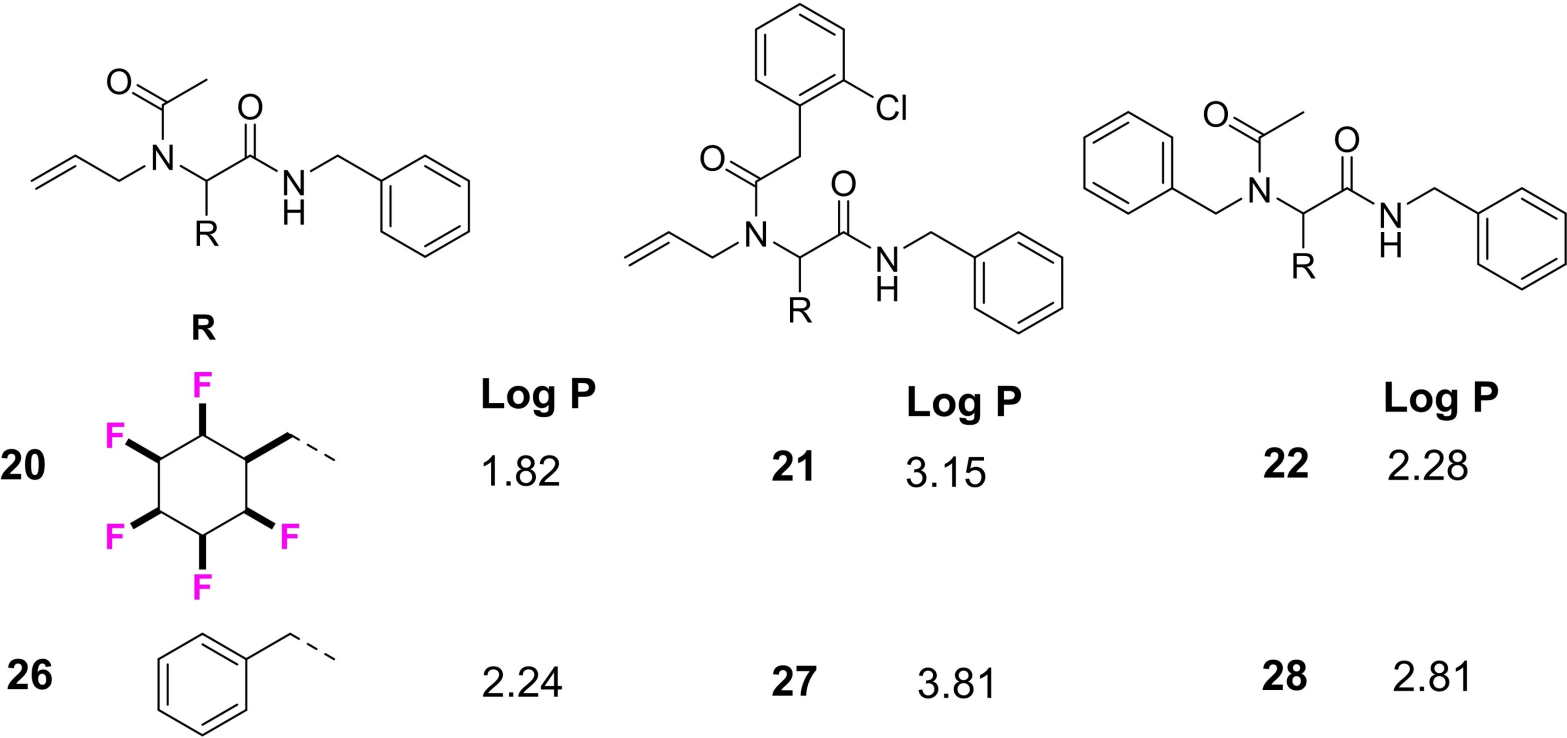
Comparative Log P values of Ugi fluorocyclohexyl products **20**–**22** with the corresponding benzyl products **26**–**28**.

Two of these products (**20** and **25**) were amenable to X‐ray structure analysis as shown in Figure 6 and this assisted in confirming their peptoid like structures. In order to elucidate the effect of the 2,3,4,5,6‐all‐*cis*‐pentafluorocyclohexane ring on lipophilicity of this product class, Log P values were determined for products **20**–**22** and compared to their corresponding analogues **26**–**28** with a phenyl ring in place of the 2,3,4,5,6‐all‐*cis*‐pentafluorocyclohexane rings. The Log P values were determined by HPLC (acetonitrile/water) as described previously[^14^, ^16^] and the comparative data are presented in Figure 7. It is clear that replacement of a phenyl ring by the all‐*cis* 2,3,4,5,6‐pentafluorocyclohexyl moiety increases hydrophilicity, with the Log P decreasing by 0.4–0.65 Log P units. This trend is consistent with that previously observed for the phenyltetrafluorocyclohexyl motifs,^[14]^ and also the all *cis*‐1,2,3‐trifluorocyclopropyl motif,^24^ and it suggests that this aspect reinforces its utility as a candidate isostere^[25]^ for hit optimisation studies.

**Figure 8:**
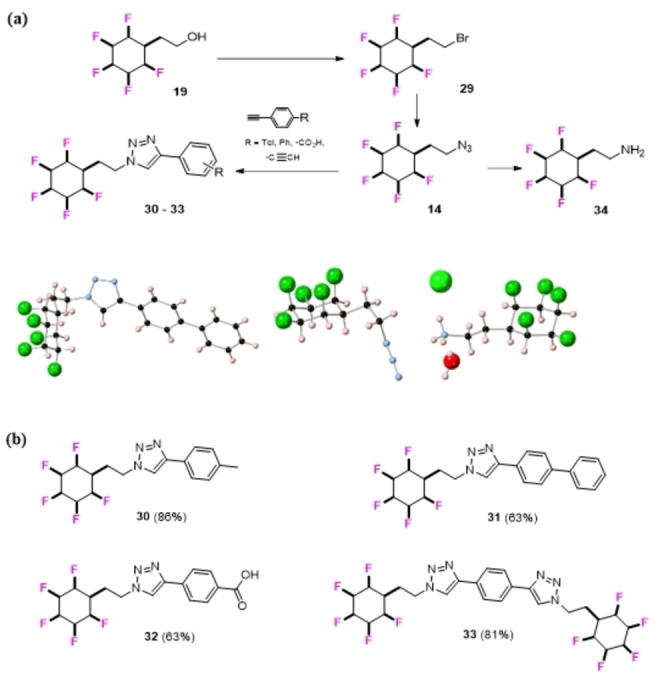
(a) Reactions forming azide **14**, amine **34** and ‘click’ products **30**–**33**. X‐ray structures of triazole **31**, azide **14**, and amine hydrochloride of **34**. (b) ‘Click’ triazole products **30**–**33** derived from **14** and an appropriate phenyl acetylene.

In other functional group interconversions, alcohol **19** was readily converted to the corresponding bromide **29** by an Appel reaction,^[26]^ and the bromide to azide **14** as shown in Figure 8a. Azide **14** is a crystalline solid and the corresponding X‐ray is also shown in Figure 8a. Azide **14** could be readily reduced under Staudinger conditions^[27]^ to amine **34** to provide another building block. Azide **14** was also a relatively efficient substrate in a series of Cu‐catalysed ‘click’ reactions^[19]^ with phenylacetylenes to give the products **30–33** as illustrated in Figure 8b.

**Figure 9:**
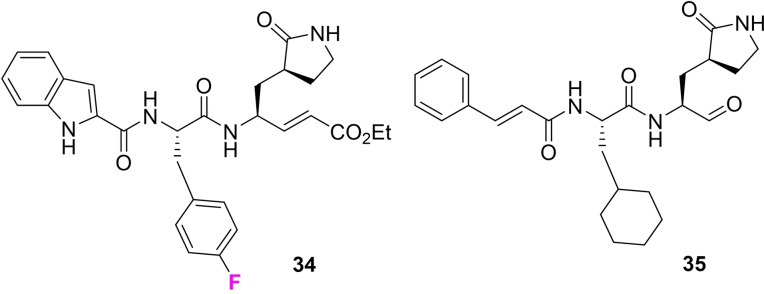
Representatives **34** and **35** of lead Enterovirus‐71 and SARS‐2 proteases inhibitors.^[20]^

**Scheme 2:**
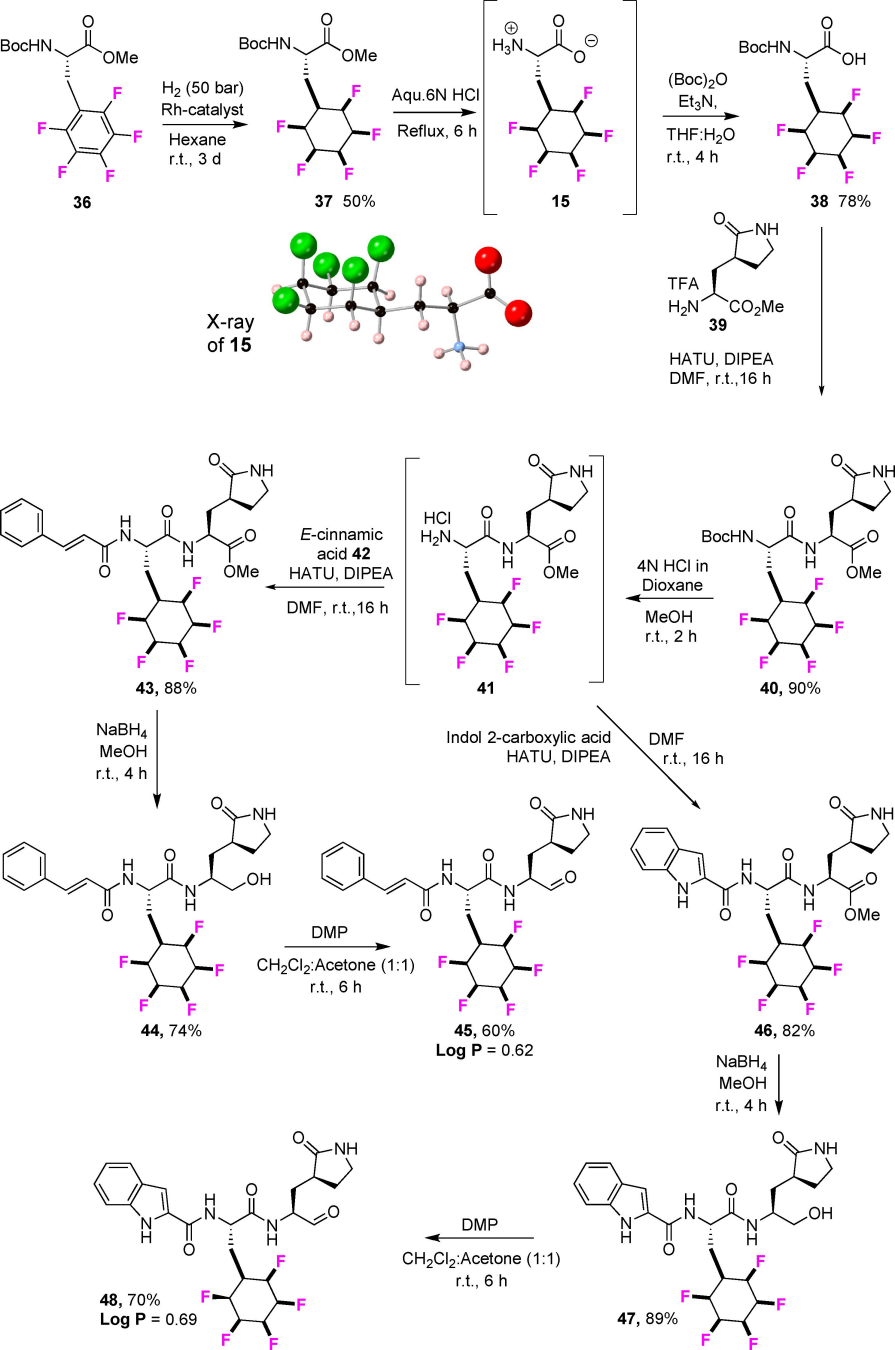
Synthesis of antiviral candidates **45** and **48** from amino acid **15**.

**Scheme 3:**
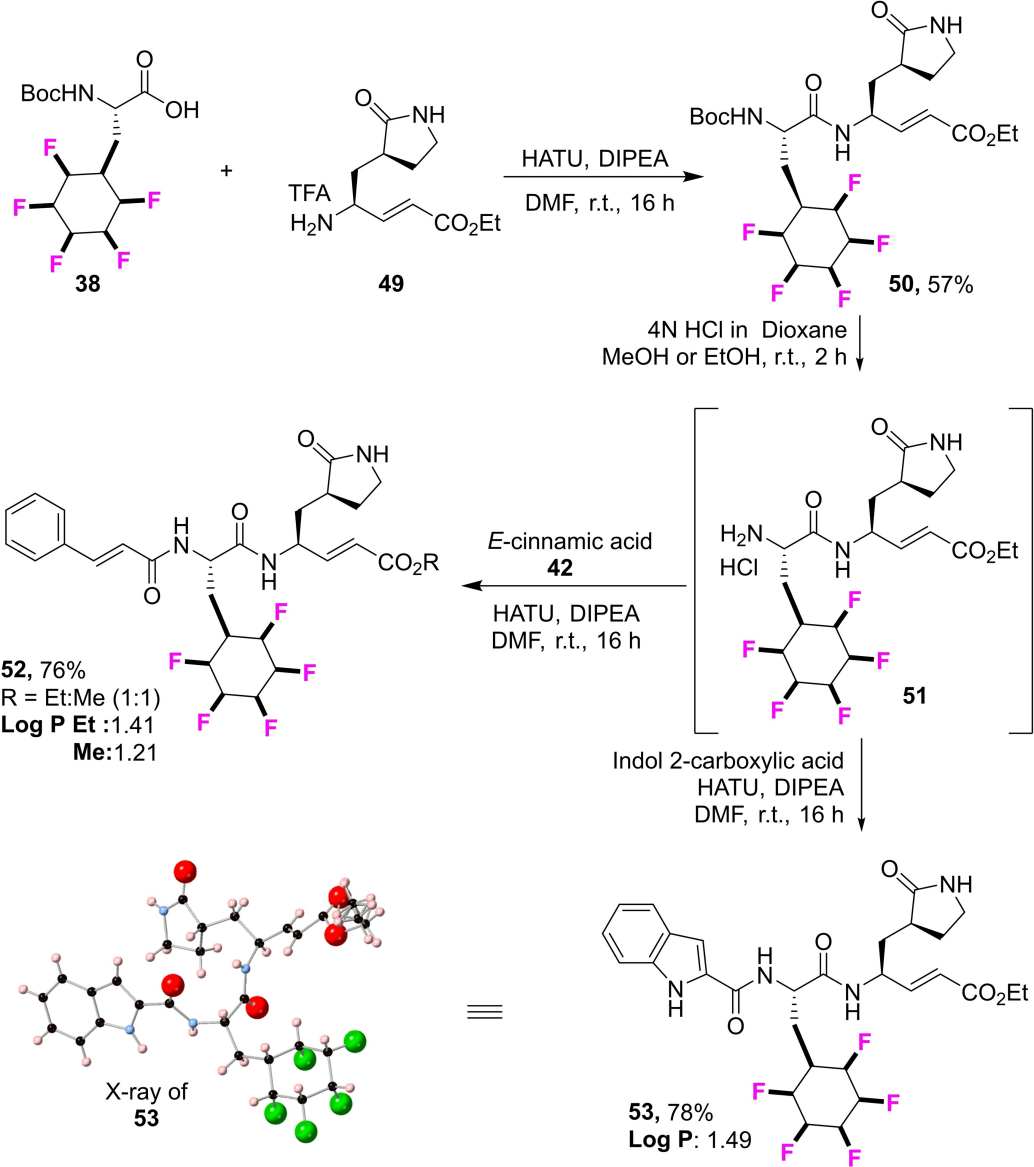
Syntheses of antiviral candidates **52** and **53**.

A potentially interesting building block of wide utility is amino acid **15**. We report its synthesis here and then its incorporation into the structural skeleton of a class of antiviral analogues typified by **34** and **35** in Figure 9. This structural series of peptidomimetic aldehydes and esters has emerged as potent inhibitors of the human enterovirus 71(EV‐71) and coronavirus (SARS) protease,^[20]^ and interest in this class of compounds has received a particular and recent focus as a starting point to find inhibitors of the COVID‐19 coronavirus.^[21]^ In particular those compounds **34** and **35** containing fluoroaryl‐ phenylalanine and cyclohexyl‐amino acid show good antiviral activities.^[20]^ In the context of exploring the all‐*cis* 2,3,4,5,6‐pentafluorocyclohexyl moiety in drug discovery, the two aldehydes **45** and **48** (Scheme 2) and the two esters **52** and **53** (Scheme 3) of this class of antivirals were prepared carrying the Janus ring in place of phenyl or cyclohexyl. These syntheses required amino acid **15** which could be prepared by aryl hydrogenation of the pentafluoroaryl analogue of fully protected phenylalanine **36**, itself prepared after methylation of the commercially available NBoc protected L‐amino acid.^[28]^ Hydrogenation using the Glorius/Zeng protocol[^16^, ^17^] generated the cyclohexylamino acid **37** in good yield. The pentafluorocyclohexyl ring system is susceptible to base decomposition, and selective ester hydrolysis under acidic conditions proved difficult, therefore the free amino acid **15** was generated and its structure confirmed by X‐ray analysis (inset in Scheme 2). This amino acid was then re‐protected with the NBoc group to give **38**.^[29]^ The synthesis to the target aldehydes **45** and **48** with either a cinnamate or indole‐carboxylate amide could then be completed following the published^[20c]^ protocols as illustrated in Scheme 2. Similarly, the corresponding conjugated ethyl ester analogues **52** and **53**, carrying the pendant cinnamate or indole‐carboxylate amides respectively were prepared also from NBoc protected amino acid **38** following the previous protocols,^[20d]^ with only minor modifications as illustrated in Scheme 3. During the Boc deprotection of **50** to get **51** in methanol, trans esterification of ethyl ester to methyl ester was observed (1 : 1 mixture) and the reaction of **51** upon reaction with **42** gave a 1 : 1 mixture of **52**. Knowing this, the de protection of Boc for the synthesis of **53** was performed in ethanol and achieved pure **53**.

Log P values were determined[^10^, ^16^] for products **45**, **48**, **52** and **53**. The values were in the range Log P ∼1.5–2.0. These are low values and in a good range for candidate drug development, and they indicate again that the all‐*cis* 2,3,4,5,6‐pentafluoro‐cyclohexyl ring system possesses good properties for bioactive molecule discovery. In the event the candidate antivirals **45**, **48**, **52** and **53** were assessed for their antiviral activity against Zika virus and the SARS‐CoV‐2 virus, however they did not show any activity in these assays.^[30]^

## Conclusion

Several building blocks have been prepared by direct aryl hydrogenation of all‐ 2,3,4,5,6‐pentafluorophenyl acetaldehyde **16** and the NBoc‐protected methyl ester of pentafluoroaryl phenylalanine. In particular a series of candidate bioactives were prepared from cyclohexylacetaldehyde **13** and amino acid **15**. Aldehyde **13**, was used to prepare a range of Ugi multicomponent products and the amino acid to access direct analogues of known peptidomimetic aldehydes and esters. Comparative Log P determinations between phenyl and all‐*cis* 2,3,4,5,6‐pentafluorocyclohexyl analogues illustrates an increase in hydrophilicity for the fluorocyclohexanes and suggests a utility for this motif in bioactives discovery programmes. These results should encourage the further exploration of this motif in medicinal chemistry.

## X‐ray crystallography

Deposition Number(s) 2098747 (**14**), 2098748 (**15**), 2098749 (**17**), 2098750 (**18**), 2098751 (**19**), 2098752 (**20**), 2098753 (**25**), 2098754 (**31**), 2098755 (**34HCl**), 2098756 (**37**), 2098757 (**53**) contain(s) the supplementary crystallographic data for this paper. These data are provided free of charge by the joint Cambridge Crystallographic Data Centre and Fachinformationszentrum Karlsruhe Access Structures service.

## Conflict of interest

The authors declare no conflict of interest.

## Supporting information

As a service to our authors and readers, this journal provides supporting information supplied by the authors. Such materials are peer reviewed and may be re‐organized for online delivery, but are not copy‐edited or typeset. Technical support issues arising from supporting information (other than missing files) should be addressed to the authors.

Supporting Information

## References

[1a] S. Meyer, J. Hafliger, R. Gilmour, *Chem. Sci*. **2021**, *12*, 10686–10695; 10.1039/d1sc02880d PMC8372324 34476053

[1b] R. Hevey, *Chem. Eur. J*. **2021**, *27*, 2240–2253; 10.1002/chem.202003135 32901973

[1c] I. Ojima (Ed.), *‘Frontiers of Organic Fluorine Chemistry’*, World Scientific Europe, **2020**;

[1d] Q. H. Liu, C. F. Ni, J. B. Hu Hu, *Nat. Sci. Rev*. **2017**, *4*, 303–325;

[1e] A. Harsanyi, G. Sandford, *Green Chem*. **2015**, *17*, 2081–2086;

[1f] P. Kirsch, ‘*Modern Fluoroorganic Chemistry. Synthesis, Reactivity, Applications*.’ 2nd Edn., Wiley-VCH, Weinheim, **2013**.

[2a] R. D. Chambers, ‘*Fluorine in Organic Chemistry’* 2^nd^ Edn., Blackwell Publishing Ltd., Oxford, **2004**;

[2b] M. Hudlicky, A. E. Pavlath, ‘*Chemistry of Organofluorine Compounds II,’* ACS Monograph 187, American Chemical Society, Washington **1995**.

[3a] W. Zhu , X. Zhen , J. Wu , Y. Cheng , J. An , X. Ma , J. Liu , Y. Qin , H. Zhu , J. Xue , X. Jiang , Nat. Commun. 2021, 12, 3957; 34172752 10.1038/s41467-021-24278-3 PMC8233348

[3b] F. Scheidt , M. Schäfer , J. C. Sarie , C. G. Daniliuc , J. J. Molloy , R. Gilmour , Angew. Chem. Int. Ed. 2018, 57, 16431–16435; 10.1002/anie.201810328 30255972

[3c] S. M. Banik , J. W. Medley , E. N. Jacobsen , J. Am. Chem. Soc. 2016, 138, 5000–5003; 27046019 10.1021/jacs.6b02391 PMC5097459

[3d] I. G. Molnar , R. Gilmour , J. Am. Chem. Soc. 2016, 138, 5004–5007; 26978593 10.1021/jacs.6b01183

[3e] T. D. Beeson , D. W. C. MacMillan , J. Am. Chem. Soc. 2005, 127, 8826–8828; 15954790 10.1021/ja051805f

[3f] L. Hintermann , A. Togni , Angew. Chem. Int. Ed. 2000, 39, 4359–4362; 10.1002/1521-3773(20001201)39:23<4359::AID-ANIE4359>3.0.CO;2-P 29711907

[4a] B. F. J. Jeffries , Z. Wang , H. R. Felstead , J. Y. Le Questel , J. Scott , E. Chiarparin , J. Graton , B. Linclau , J. Med. Chem. 2020, 63, 1002–1031; 31894985 10.1021/acs.jmedchem.9b01172

[4b] Q. A. Huchet , N. Trapp , B. Kuhn , B. Wagner , H. Fischer , N. A. Kratochwil , E. M. Carreira , K. Müller , J. Fluorine Chem. 2017, 198, 34–46;

[4c] R. Vorberg , N. Trapp , D. Zimmerli , B. Wagner , H. Fischer , N. A. Kratochwil , M. Kansy , E. M. Carreira , K. M. Müller , ChemMedChem 2016, 11, 2216–2239; 27629993 10.1002/cmdc.201600325

[4d] H.-J Bôhm , D. Banner , S. Bendels , M. Kansy , B. Kuhn , K. Müller , U. Obst-Sander , M. Stahl , ChemBioChem. 2004, 5, 637–643. 15122635 10.1002/cbic.200301023

[5a] D. O'Hagan , Chem. Eur. J. 2020, 26, 7981–7997; 32083392 10.1002/chem.202000178

[5b] N. Al-Maharik , D. B. Cordes , A. M. Z. Slawin , M. Bühl , D. O'Hagan , Org. Biomol. Chem. 2020, 18, 878–887; 31942902 10.1039/c9ob02647a

[5c] D. O′Hagan , J. Org. Chem. 2012, 77, 3689–3699; 22404655 10.1021/jo300044q

[5d] L. Hunter , P. Kirsch , A. M. Z. Slawin , D. O'Hagan , Angew. Chem. Int. Ed. 2009, 48, 5457–5460; 10.1002/anie.200901956 19551794

[6] N. S. Keddie , A. M. Z. Slawin , T. Lebl , D. Philp , D. O'Hagan , Nat. Chem. 2015, 7, 483–488. 25991526 10.1038/nchem.2232

[7] R. A. Cormanich , N. Keddie , R. Rittner , D. O'Hagan , M. Bühl , Phys. Chem. Chem. Phys. 2015, 44, 29475–29478. 10.1039/c5cp04537a 26507700

[8] B. E. Ziegler , M. Lecours , R. A. Marta , J. Featherstone , E. Fillion , W. S. Hopkins , V. Steinmetz , N. S. Keddie , D. O'Hagan , T. B. McMahon , J. Am. Chem. Soc. 2016, 138, 7460–7463. 27149387 10.1021/jacs.6b02856

[9] M. J. Lecours , R. A. Marta , V. Steinmetz , N. Keddie , E. Fillion , D. O'Hagan , T. B. McMahon , W. S. Hopkins , J. Phys. Chem. Lett. 2017, 8, 109–113. 27966976 10.1021/acs.jpclett.6b02629

[10] J. L. Clark , A. Taylor , A. Geddis , R. M. Neyyappadath , B. A. Piscelli , C. Yu , D. B. Cordes , A. M. Z. Slawin , R. A. Cormanich , S. Guldin , D. O'Hagan , Chem. Sci. 2021, 12, 9712–9719. 34349942 10.1039/d1sc02130c PMC8293821

[11] N. Santschi , R. Gilmour , Nat. Chem. 2015, 7, 467–468. 25991520 10.1038/nchem.2240

[12] O. Shyshov , K. A. Siewerth , M. von Delius , Chem. Commun. 2018, 54, 4353–4355. 10.1039/c8cc01797b 29645043

[13] O. Shyshov , S. V. Haridas , L. Pesce , H. Qi , A. Gardin , D. Bochicchio , U. Kaiser , G. M. Pavan , M. von Delius , Nat. Commun. 2021, 12, 3134. 34035277 10.1038/s41467-021-23370-y PMC8149861

[14a] T. Bykova, N. Al-Maharik, A. M. Z. Slawin, M. Bühl, T. Lebl, D. O’ Hagan, *Chem. Eur. J*. **2018**, *24*, 13290–13296; 10.1002/chem.201802166 29882357

[14b] T. Bykova, N. Al-Maharik, A. M. Z. Slawin, D. O'Hagan, *Beilstein J. Org. Chem*. **2017**, *13*, 728–733; 10.3762/bjoc.13.72 PMC5405691 28503208

[14c] T. Bykova , N. Al-Maharik , A. M. Z. Slawin , D. O'Hagan , Org. Biomol. Chem. 2016, 14, 1117–1123; 26646211 10.1039/c5ob02334c

[14d] M. S. Ayoup , D. B. Cordes , A. M. Z. Slawin , D. O'Hagan , Org. Biomol. Chem. 2015, 13, 5621–5624; 25900403 10.1039/c5ob00650c

[14e] M. Salah Ayoup , D. B. Cordes , A. M. Z. Slawin , D. O'Hagan , Beilstein J. Org. Chem. 2015, 11, 2671–2676; 26877788 10.3762/bjoc.11.287 PMC4734450

[14f] A. J. Durie , T. Fujiwara , R. Cormanich , M. Bűhl , A. M. Z. Slawin , D. O'Hagan , Chem. Eur. J. 2014, 20, 6259–6263. 24740763 10.1002/chem.201400354

[15a] Q. Wang , D.-X. Wang , M.-X. Wang , J. Zhu , Acc. Chem. Res. 2018, 51, 1290–1300; 29708723 10.1021/acs.accounts.8b00105

[15b] I. Ugi , A. Dömling , W. Hörl , Endeavour 1994, 18, 115–122.

[16] A. Rodil , S. Bosisio , M. S. Ayoup , L. Quinn , D. B. Cordes , A. M. Z. Slawin , C. D. Murphy , J. Michel , D. O'Hagan , Chem. Sci. 2018, 9, 3023–3028. 29732086 10.1039/c8sc00299a PMC5916015

[17a] Z. Nairoukh , M. Wollenburg , C. Schlepphorst , K. Bergander , F. Glorius , Nat. Chem. 2019, 11, 264–270; 30664720 10.1038/s41557-018-0197-2 PMC6522351

[17b] M. P. Wiesenfeldt , T. Knecht , C. Schlepphorst , F. Glorius , Angew. Chem. Int. Ed. 2018, 57, 8297–8300; 10.1002/anie.201804124 29790639

[17c] M. P. Wiesenfeldt , Z. Nairoukh , W. Li , F. Glorius , Science 2017, 357, 908–912. 28798044 10.1126/science.aao0270

[18a] X. Zhang , L. Ling , M. Luo , X. Zeng , Angew. Chem. Int. Ed. 2019, 58, 16785–16789; 10.1002/anie.201907457 31518488

[18b] Y. Wei , B. Rao , X. Cong , X. Zeng , J. Am. Chem. Soc. 2015, 137, 9250–9253. 26172049 10.1021/jacs.5b05868

[19a] C. W. Tornøe , C. Christensen , M. Meldal , J. Org. Chem. 2002, 67, 3057–3064; 11975567 10.1021/jo011148j

[19b] V. V. Rostovtsev , L. G. Green , V. V. Fokin , K. B. Sharpless , Angew. Chem. Int. Ed. 2002, 41, 2596–2599; 10.1002/1521-3773(20020715)41:14<2596::AID-ANIE2596>3.0.CO;2-4 12203546

[20a] W. Dai , D. Jochmans , H. Xie , H. Yang , J. Li , H. Su , D. Chang , J. Wang , J. Peng , L. Zhu , Y. Nian , R. Hilgenfeld , H. Jiang , K. Chen , L. Zhang , Y. Xu , J. Neyts , H. Liu , J. Med. Chem. 2021, 64, 10.1021/acs.jmedchem.0c02258; 33872498

[20b] W. Dai , B. Zhang , X.-M. Jiang , H. Su , J. Li , Y. Zhao , X. Xie , Z. Jin , J. Peng , F. Liu , C. Li , Y. Li , F. Bai , H. Wang , X. Cheng , X. Cen , S. Hu , X. Yang , J. Wang , X. Liu , G. Xiao , H. Jiang , Z. Rao , L.-K. Zhang , Y. Xu , H. Yang , H. Liu , Science 2020, 363, 1331–1335; 10.1126/science.abb4489 PMC7179937 32321856

[20c] Y. Zhai , X. Zhao , Z. Cui , M. Wang , Y. Wang , L. Li , Q. Sun , X. Yang , D. Zeng , Y. Liu , Y. Sun , Z. Lou , L. Shang , Z. Yin , J. Med. Chem. 2015, 58, 9414–9420; 26571192 10.1021/acs.jmedchem.5b01013

[20d] C.-J. Kuo , J.-J. Shie , J.-M. Fang , G.-R. Yen , J. T. A. Hsu , H.-G. Liu , S.-N. Tseng , S.-C. Chang , C.-Y. Lee , S.-R. Shih , P.-H. Liang , Bioorg. Med. Chem. 2008, 16, 7388–7398; 18583140 10.1016/j.bmc.2008.06.015 PMC7125518

[20e] K. Anand , J. Ziebuhr , P. Wadhwani , J. R. Mesters , R. Hilgenfeld , Science 2003, 300, 1763–1767. 12746549 10.1126/science.1085658

[21a] R. L. Hoffman , R. S. Kania , M. A. Brothers , J. F. Davies , R. A. Ferre , K. S. Gajiwala , M. He , R. J. Hogan , K. Kozminski , L. Y. Li , J. W. Lockner , J. Lou , M. T. Marra , L. J. Mitchell , B. W. Murray , J. A. Nieman , S. Noell , S. P. Planken , T. Rowe , K. Ryan , G. J. Smith , J. E. Solowiej , C. M. Steppan , B. Taggart , J. Med. Chem. 2020, 63, 12725–12747; 33054210 10.1021/acs.jmedchem.0c01063

[21b] L. Zhang , D. Lin , Y. Kusov , Y. Nian , Q. Ma , J. Wang , A. von Brunn , P. Leyssen , K. Lanko , J. Neyts , A. de Wilde , E. J. Snijder , H. Liu , R. Hilgenfeld , J. Med. Chem. 2020, 63, 4562–4578. 32045235 10.1021/acs.jmedchem.9b01828

[22] D. B. Dess , J. C. Martin , J. Org. Chem. 1983, 48, 4155–4156.

[23a] C. Giaginis , A. Tsantili-Kakoulidou , J. Liq. Chromatogr. Relat. Technol. 2008, 31, 79–96;

[23b] C. M. Du , K. Valko , C. Bevan , D. Reynolds , M. H. Abraham , Anal. Chem. 1998, 70, 4228–4234.

[24] Z. Fang , D. B. Cordes , A. M. Z. Slawin , D. O'Hagan , Chem. Commun. 2019, 55, 10539–10542. 10.1039/c9cc05749h 31414105

[25a] B. M. Johnson , Y. Z. Shu , X. L. Zhuo , N. A. Meanwell , J. Med. Chem. 2020, 63, 6315–6386; 32182061 10.1021/acs.jmedchem.9b01877

[25b] H. Mei , J. Han , S. White , D. J. Graham , K. Izawa , T. Sato , S. Fustero , N. A. Meanwell , V. A. Soloshonok , Chem. Eur. J. 2020, 26, 11349–11390; 32359086 10.1002/chem.202000617

[25c] E. P. Gillis , K. J. Eastman , M. D. Hill , D. J. Donnelly , N. A. Meanwell , J. Med. Chem. 2015, 58, 8315–8359. 26200936 10.1021/acs.jmedchem.5b00258

[26] R. Appel , Angew. Chem. Int. Ed. 1975, 14, 801–811;

[27] Y. G. Gololobov , L. F. Kasukhin , Tetrahedron 1992, 48, 1353–1406.

[28] P. P. Geurink , N. Liu , M. P. Spaans , S. L. Downey , A. M. C. H. van den Nieuwendijk , G. A. van der Marel , A. F. Kisselev , B. I. Florea , H. S. Overkleeft , J. Med. Chem. 2010, 53, 2319–2323; N-Boc-L-(pentafluoroaryl)phenylalanine - Fluorochem. Ltd., Catalogue No 008086; CAS Number 34702–60-8. 20131905 10.1021/jm9015685 PMC3037272

[29] E. Vaz , M. Fernandez-Saurez , L. Muñoz , Tetrahedron: Asymmetry 2003, 14, 1935–1942.

[30] Antiviral assays were conducted the MRC-Centre for Virus Research at the University of Glasgow, UK.

