# Supplementary material for: Janus All‐Cis 2,3,4,5,6‐Pentafluorocyclohexyl Building Blocks Applied to Medicinal Chemistry and Bioactives Discovery Chemistry
Source: Chemistry. 2021 Oct 6;27(64):16000–5. doi: 10.1002/chem.202102819 (PMC9292521; doi:10.1002/chem.202102819)
Supplement: Supplementary file 1 — Supporting Information [file CHEM-27-16000-s001.pdf]

Chemistry–A European Journal

Supporting Information

Janus All-*Cis* 2,3,4,5,6-Pentafluorocyclohexyl Building Blocks Applied to Medicinal Chemistry and Bioactives Discovery Chemistry

Joshua L. Clark, Rifahath M. Neyyappadath, Cihang Yu, Alexandra M. Z. Slawin, David B. Cordes, and David O'Hagan*

Synthetic Protocols and Characterisation Data

Compounds **39** and **49** was synthesized and characterized according to the literature.[1]

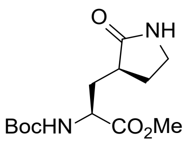

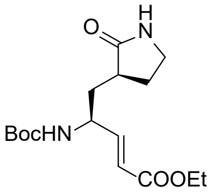

Chloro[2-(2,6-diisopropylphenyl)-3,3-dimethyl-2-azaspiro[4.5]dec-1-ylidene][1,2,5,6-η-1,5-cyclooctadiene]rhodium 12[2]

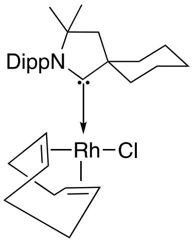

$[RhCODCl]_2$ (77 mg, 0.16 mmol), 2-(2,6-Diisopropylphenyl)-3,3-dimethyl-2-azaspiro[4.5]dec-1-en-2-ium hydrogen dichloride (131 mg, 0.329 mmol) and KHMDS (150 mg, 0.750 mmol) were added to a Schlenk tube inside an argon-filled glovebox. THF (10 mL) was added dropwise over 10 min at -78 °C. The resulting suspension was stirred for 10 min at -78 °C, and then warmed to room temperature and stirred for 16 h. The mixture was filtered and concentrated *in vacuo* to give the crude product, which was purified by flash column chromatography (19:1 pentane:Et_2O). The pure fractions were combined and concentrated *in vacuo* to give an oily residue. The residue was redissolved in CH_2Cl_2 (1 mL) and **12** was precipitated by dropwise addition of pentane. The excess solvent was decanted giving **12** as a yellow powder (97 mg, 0.170 mmol, 53%): ^{1}H NMR ($CDCl_3$, 500 MHz) δ_H 7.46-7.38 (2H, m, ArH), 7.14 (1H, dd *J* = 7.5, 1,5, ArH), 5.24 (1H, t, *J* = 7.6), 4.60 (1H, q, *J* = 7.8), 3.94-3.85 (1H, m), 3.48-3.42 (1H, m), 2.92-2.84 (2H, m), 2.64-2.45 (3H, m), 2.31-2.24 (1H, m), 2.20-2.09 (1H, m), 2.04-1.91 (3H, m), 1.79-1.72 (7H, m), 1.60-1.52 (2H, m), 1.50 (s, 3H), 1.46-1.30 (3H, m), 1.27-1.22 (9H, m), 1.20 (3H, s), 0.95 (3H, d, J = 6.7); ^{13}C NMR ($CDCl_3$, 125 MHz) δ_C 148.2, 146.4, 137.0, 129.0, 126.5, 124.1, 101.3 (d, J = 6.1 Hz), 98.2 (d, J = 5.8 Hz), 78.2, 71.9 (d, J = 15.0 Hz), 64.8, 64.6 (d, J = 13.9 Hz), 45.6, 41.8, 38.1, 35.0, 33.7, 30.9, 30.3, 28.9, 28.3, 28.0, 26.6, 26.4, 26.1, 25.9, 25.5, 24.6, 24.0, 22.6; data are in agreement with literature.[2]

Methyl 2-((*1r,2R,3R,4s,5S,6S*)-2,3,4,5,6-pentafluorocyclohexyl)acetate 17

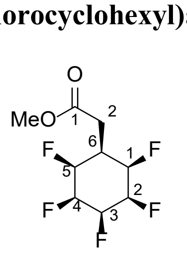

Activated 4 Å molecular sieves (7 g), **16** (0.850 g, 3.54 mmol) and **12** (16 mg, 0.028 mmol, 1 mol%) were suspended in hexane (40 mL) in a vial and the vial placed inside an autoclave. The autoclave was pressurised with hydrogen to 50 Bar and the reaction mixture stirred at room temperature for 16 h. After depressurising and removing the vial, the suspension was filtered and concentrated *in vacuo* to give the crude product, which was purified by flash column chromatography (SiO_2, 50% EtOAc in hexane) to give **17** as a white crystalline solid (0.662 g, 2.69 mmol, 76%);

m.p. (acetone): 151 °C; ^{1}H NMR (700 MHz, Acetone-d_6) δ_H 5.50-5.38 (1H, m, FCH-3), 5.12-4.83 (overlapping m, FCH-1, FCH-2, FCH-4, FCH-5), 3.70 (3H, s, OCH_3), 2.85 (2H, d J = 7.2 Hz, CH_2-2) , 2.64-2.47 (1H, m, FCCH-6); ^{13}C NMR (126 MHz, Acetone-d_6) δ_C 172.4 (C=O), 89.5 (C-F), 88.0 (C-F), 86.6 (C-F), 52.2 (O*C*H_3), 36.0 (C-6), 31.0 (H_2*C*-2); ^{19}F NMR (659 MHz, Acetone-d_6) δ_F −205.5, −212.5, −217.6; HRMS m/z (ESI$^+$) (calculated $C_9H_{12}F_5O_2^+$ = 247.0752) found 247.0750 $(M+H)^+$; ν_{max}/cm^{-1} 1728 (C=O).

Methyl 2-((*1S,2S,3R,4R,6S*)-2,3,4,6-tetrafluorocyclohexyl)acetate 18

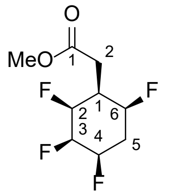

Activated 4 Å molecular sieves (7 g), **16** (0.850 g, 3.54 mmol) and **12** (16 mg, 0.028 mmol, 1 mol%) were suspended in hexane (40 mL) in a vial and the vial placed inside an autoclave. The autoclave was pressurised with hydrogen to 50 Bar and the reaction mixture stirred at room temperature for 16 h. After depressurising and removing the vial, the suspension was filtered and concentrated *in vacuo* to give the crude product, which was purified by flash column chromatography (SiO_2, 50% EtOAc in hexane) to give **18** as a white crystalline solid (0.049 g, 0.21 mmol, 6%); m.p. (acetone): 129-130 °C; ^{1}H NMR (500 MHz, Acetone-d_6) δ_H 5.17-4.78 (4H, overlapping m, FCH-2, FCH-3 FCH-4 and FCH-6), 3.68 (3H, s, OCH_3), 2.76 (2H, d J = 6.9 Hz, CH_2-2), 2.67-2.48 (2H, overlapping m, FCCH-1 and CH-5a), 2.19 (1H, app dtt J = 39.9, 16.3, 3.3 Hz, CH-5b); ^{19}F NMR (470 MHz, Acetone-d_6) δ_F −191.65, −199.85, −201.6, −211.6; ^{13}C NMR (126 MHz, Acetone-d_6) δ_C 172.7 (*C*=O), 89.3 (*C*F), 87.55 (*C*F), 86.7 (*C*F), 52.1 (O*C*H_3), 39.35 (FCC-1), 33.35 (C-5), 31.65 (*C*H_2-2); HRMS m/z ESI$^+$ (Calculated $C_9H_{12}F_4O_2Na^+$ = 251.0666) found 251.0664 $[M+Na]^+$; ν_{max}/cm^{-1} 1726 (C=O), 1094 and 1045 (C-F).

2-((*1r,2R,3R,4s,5S,6S*)-2,3,4,5,6-Pentafluorocyclohexyl)ethan-1-ol 19

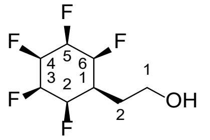

DIBAlH (1M in hexane, 9.7 mL, 9.7 mmol) was added dropwise to a solution of **17** (0.588 g, 2.39 mmol) in THF (20 mL) at 0 °C. The solution was slowly warmed to r.t. and stirred for 16 h. The reaction was then diluted with Et_2O (50 mL) cooled to 0 °C and quenched by the slow subsequent addition of water (0.4 mL), aqueous sodium hydroxide (15% w/w, 0.4 mL) and water (1 mL). The mixture was warmed to r.t. and stirred for 15 mins before $MgSO_4$ was added and the resulting suspension stirred for 15 mins. The suspension was then filtered to remove aluminium salts and the filtrate concentrated *in vacuo*. The crude product was purified by flash column chromatography (SiO_2, 60% EtOAc in hexane to 100% EtOAc) to give **19** as a white crystalline solid (0.433 g, 1.98 mmol, 83%) as a crystalline white solid: m.p. (MeOH): 126-127 °C; ^{1}H NMR (500 MHz, Methanol-d_4) δ_H 5.34-5.18 (1H, m, FCH-4), 5.01-4.87 (2H, m, FCH-2 and FCH-6), 4.77-4.52 (2H, m, FCH-3 and FCH-5), 3.73 (2H, d J = 6.0 Hz, CH_2-1), 2.11-1.85 (3H, overlapping m, CH_2-2 and FCCH-1); ^{19}F NMR (659 MHz, Methanol-d_4) δ_F -205.8, -213.2, -218.2; ^{13}C NMR (126 MHz, Methanol-d_4): δ_C 89.9 (FC-4), 88.4 (FC-2 and FC-6), 87.1 (FC-3 and FC-5), 59.3 (H_2C-1), 36.0 (FCC-1), 29.8 (H_2C-2); HRMS m/z (ESI+) (calculated $C_8H_{11}F_5ONa^+$ = 241.0622) found 241.0618 $[M+Na]^+$; ν_{max}/cm^{-1} 3400 br (O-H), 2940 (C-H), 1364 (O-H), 1045 (C-O).

2-((*1r,2R,3R,4s,5S,6S*)-2,3,4,5,6-Pentafluorocyclohexyl)acetaldehyde 13

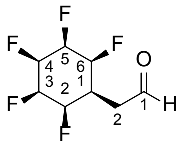

To a solution of **19** (0.346 g, 1.41 mmol) in THF (21 mL) was added dess-martin periodinane (0.88 g, 2.1 mmol) at 0 °C. The suspension was warmed to r.t. and stirred for 45 mins until complete consumption of starting material was observed by thin layer chromatography. The reaction was quenched by the addition of sodium thiosulfate solution (25% w/w, 9.7 mL) and saturated $NaHCO_3$ solution (9.7 mL). The mixture was stirred for 30 mins before being extracted into EtOAc (3 x 50 mL). The combined organic phase was dried over $MgSO_4$, filtered and concentrated *in vacuo*. The residue was purified by flash column chromatography (SiO_2, 40% EtOAc in hexane to 60% EtOAc in hexane) to give **13** as a white crystalline solid, (0.259 g, 1.20 mmol, 85%), only peaks for the keto form are assigned: m.p. (acetone) 205-206 °C; ^{1}H NMR (500 MHz, Acetone-d_6) δ_H 9.85 (1H, s, C(O)H-1), 5.51-5.34 (1H, m, FCH-4), 5.10-4.88 (4H, overlapping m, FCH-2, FCH-3, FCH-5 and FCH-6), 3.07 (2H, d J = 6.7 Hz, CH_2-2), 2.79-2.59 (1H, m, FCCH-1); ^{19}F NMR (471 MHz, Acetone-d_6) δ_F −205.3, −212.1, −217.6; ^{13}C NMR (126 MHz, Acetone-d_6) δ_C 200.6 (*C*=O), 89.7 (*C*F), 88.1 (*C*F), 86.5 (*C*F), 40.6 (H_2C-2), 33.3 (FCC-1); HRMS m/z ESI$^+$ (Calculated $C_8H_9F_5ONa^+$ = 239.0466) found 239.0463 [M+Na]$^+$; ν_{max}/cm^{-1} 2820 (C-H), 1721 (C=O), 1398 (C-H).

2-(N-Allylacetamido)-N-benzyl-3-((*1r,2R,3R,4s,5S,6S*)-2,3,4,5,6-pentafluorocyclohexyl)propenamide 20

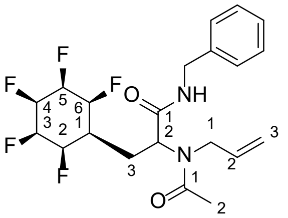

Allylamine (0.012 mL, 0.16 mmol) was added to a solution of **13** (0.034 g, 0.16 mmol) in MeOH (0.5 mL) in a microwave vial and the solution was stirred for 5 mins. Then, acetic acid (0.0080 mL, 0.13 mmol) and benzyl isocyanide (0.016 mL, 0.13 mmol) were added and the vial sealed. The solution was heated in a 2.45 GHz microwave reactor targeting 45 °C for 45 mins before being concentrated *in vacuo*. The residue was redissolved in EtOAc (5 mL) and washed with brine (3 x 5 mL). The organic phase was dried over $MgSO_4$, filtered and concentrated *in vacuo* to give the crude product which was purified by flash column chromatography (SiO_2, 70% EtOAc in hexane to 90% EtOAc in hexane) to give **20** as a white crystalline solid (0.040 g, 0.093 mmol, 72%): m.p. ($CHCl_3$): 158-159 °C; ^{1}H NMR (500 MHz, $CDCl_3$) δ_H 7.33-7.22 (5H, overlapping m, ArH), 5.75-5.67 (1H, m, NCH_2CH-2), 5.35-5.13 (4H, overlapping m, NCH_2CHCH_2-3, NCH-2 and FCH-4), 5.02-4.86 (2H, m, FCH-2 and FCH-6), 4.46-4.20 (4H, overlapping m, $PhCH_2$, FCH-3 and FCH-5), 4.03-3.89 (2H, m, NCH_2-1) 2.51-2.42 (1H, m, $FCHCHCH_2$-3a), 2.21-2.13 (1H, m, $FCHCHCH_2$-3b), 2.07 (3H, s, CH_3), 1.73-1.54 (1H, m, FCHCH-1); ^{19}F NMR (470 MHz, $CDCl_3$) δ_F −203.4, −211.45, −216.7; ^{13}C NMR (126 MHz, $CDCl_3$) δ_C 173.1 (C=O), 170.0 (C=O), 138.1 (ArC), 133.3 (C=C-2), 128.9 (Ar*C*H), 127.9 (Ar*C*H), 127.7 (Ar*C*H), 117.9 (C=C-3), 88.2 (*C*F), 87.2 (*C*F), 86.2 (*C*F), 54.0 (N*C*H-2), 48.35 (N*C*H_2-1), 43.6 (Ph*C*H_2), 35.9 (FCH*C*H-1), 25.5 (FCHCH*C*H_2-3), 22.1 (H_3C-2); HRMS m/z ESI$^+$ (Calculated $C_{21}H_{25}F_5O_2N_2Na^+$ = 455.1728) found 455.1718 [M+Na]$^+$; ν_{max}/cm^{-1} 1624 (C=O), 1134 and 1049 (C-F).

2-(N-Allyl-2-(2-chlorophenyl)acetamido)-N-benzyl-3-((*1r,2R,3R,4s,5S,6S*)-2,3,4,5,6-pentafluorocyclohexyl)propenamide 21

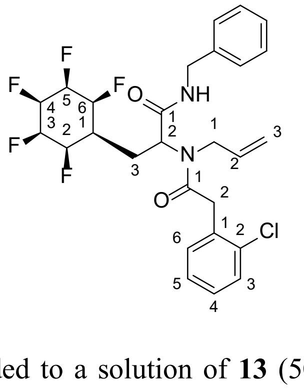

Allylamine (0.017 mL, 0.23 mmol) was added to a solution of **13** (50 mg, 0.23 mmol) in MeOH (0.5 mL) in a microwave vial and the solution was stirred for 5 mins. Then, 2-chlorophenylacetic acid (0.032 g, 0.19 mmol) and benzyl isocyanide (0.023 mL, 0.19 mmol) were added and the vial sealed. The solution was heated in a 2.45 GHz

microwave reactor targeting 45 °C for 45 mins before being concentrated *in vacuo*. The residue was redissolved in EtOAc (5 mL) and washed with brine (3 x 5 mL). The organic phase was dried over $MgSO_4$, filtered and concentrated *in vacuo* to give the crude product which was purified by flash column chromatography (SiO_2, 20% EtOAc in hexane to 40% EtOAc in hexane) to give **21** as a white crystalline solid (0.082 g, 0.15 mmol, 79%): m.p. ($CHCl_3$): 182-183 °C; ^{1}H NMR (500 MHz, $CDCl_3$) δ_H 7.38-7.19 (9H, overlapping m, ArH), 6.92 (1H, t *J* = 5.4 Hz, NH), 5.81-5.73 (1H, m, C=CH-2), 5.35-5.20 (3H, overlapping m, FCH-4 and C=C-H_2-3), 5.08 (1H, t *J* = 7.3 Hz, NCH-2), 5.02-4.96 (1H, m, FCH-2a and FCH-6a), 4.92-4.85 (1H, m, FCH-2b and FCH-6b), 4.39-4.25 (4H, overlapping m, FCH-3, FCH-5 and $PhCH_2$), 4.13-4.00 (2H, m, NCH_2-1), 3.77 (2H, s, $ArCH_2$) 2.58-2.53 (1H, m, $FCHCHCH_2$-3a), 2.26-2.18 (1H, m, $FCHCHCH_2$-3b), 1.84-1.54 (1H, m, FCHCH-1); ^{19}F NMR (471 MHz, $CDCl_3$) δ_F −203.3, −203.6, −211.2, −211.65, −216.75; ^{13}C NMR (126 MHz, $CDCl_3$) δ_C 172.6 (C=O), 169.8 (C=O), 137.8 (ArC), 134.1 (ArC), 133.1 (C=C-2), 131.6 (Ar*C*H), 129.6 (Ar*C*H), 129.05 (Ar*C*H), 128.9 (Ar*C*H), 128.0 (Ar*C*H), 127.8 (Ar*C*H), 127.4 (Ar*C*H), 118.6 (C=C-3), 88.2 (*C*F), 86.3 (*C*F), 85.1 (*C*F), 54.9 (N*C*H-2), 48.3 (N*C*H_2-1), 43.8 (Ph*C*H_2), 38.9 (Ar*C*H_2), 35.9 (FCH*C*H-1), 25.4 ($FCHCHCH_2$-3); HRMS m/z ESI$^+$ (Calculated $C_{27}H_{28}F_5ClN_2O_2Na^+$ = 565.1652) found 565.1650 $[M+Na]^+$; ν_{max}/cm^{-1} 1682 and 1626 (C=O), 1136 (C-F).

N-Benzyl-2-(N-benzylacetamido)-3-((*1r,2R,3R,4s,5S,6S*)-2,3,4,5,6-pentafluorocyclohexyl)propenamide 22

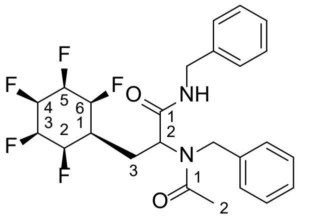

Benzylamine (0.030 mL, 0.28 mmol) was added to a solution of **13** (0.054 g, 0.25 mmol) in MeOH (0.5 mL) in a microwave vial and the solution was stirred for 5 mins. Then, acetic acid (0.016 mL, 0.28 mmol) and benzyl isocyanide (0.033 mL, 0.28 mmol) were added and the vial sealed. The solution was heated in a 2.45 GHz microwave reactor targeting 45 °C for 45 mins before being concentrated *in vacuo*. The residue was redissolved in EtOAc (5 mL) and washed with brine (3 x 5 mL). The organic phase was dried over $MgSO_4$, filtered and concentrated *in vacuo* to give the crude product which was purified by flash column chromatography (SiO_2, 80% EtOAc in hexane) to give **22** as a white crystalline solid (0.049 g, 0.10 mmol, 41%): m.p. (MeOH): 142-143 °C; ^{1}H NMR (500 MHz, $CDCl_3$) δ_H 7.36-7.17 (10H, overlapping m, ArH, 5.30-5.16 (1H, m, FCH-4), 5.10-5.03 (1H, m, NCH-2), 4.91-4.78 (1H, m, FCH-2a and FCH-6a), 4.70-4.60 (2H, m, $PhCH_2$), 4.54-4.44 (1H, m, FCH-2b and FCH-6b), 4.38-4.27 (1H, m, $PhCH_2$), 4.35-4.04 (2H, m, FCH-3 and FCH-5), 2.48-2.39 (1H, m, $FCHCHCH_2$-3a), 2.15 (3H, s, CH_3-2), 2.16-2.07 (1H, m, $FCHCHCH_2$-3b), 1.64-1.47 (1H, m, FCHCH-1); ^{19}F NMR (470 MHz, $CDCl_3$) δ_F −203.4, −203.5, −211.4, −211.8, −216.9; ^{13}C NMR (126 MHz, $CDCl_3$) δ_C 173.4 (*C*=O), 170.0 (*C*=O), 138.0 (ArC), 136.8 (ArC), 129.3 (Ar*C*H), 128.9 (Ar*C*H), 128.2 (Ar*C*H), 127.8 (Ar*C*H), 127.75 (Ar*C*H), 126.5 (Ar*C*H), 87.8 (*C*F), 86.9 (*C*F), 86.0 (*C*F), 54.85 (N*C*H-2), 50.0 (Ph*C*H_2), 43.6 (Ph*C*H_2), 35.6 (FCH*C*H-1), 25.55 ($FCHCHCH_2$-3), 22.5 (H_3C-2); HRMS m/z ESI$^+$ (Calculated $C_{25}H_{27}F_5N_2O_2Na^+$ = 505.1885) found 505.1875 $[M+Na]^+$; ν_{max}/cm^{-1} 1641 (C=O), 1140 (C-F).

***tert*-Butyl ((2S)-1-(benzyl(1-(benzylamino)-1-oxo-3-((*1r,2R,3R,4R,5S,6S*)-2,3,4,5,6-pentafluorocyclohexyl)propan-2-yl)amino)-3-methyl-1-oxobutan-2-yl)carbamate 23**

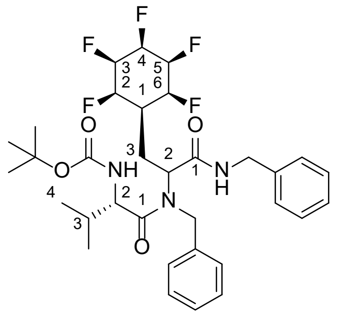

Benzylamine (0.031 mL, 0.29 mmol) was added to a solution of **13** (62 mg, 0.29 mmol) in MeOH (0.5 mL) in a microwave vial and the solution was stirred for 5 mins. Then, Boc-L-valine (0.052 g, 0.24 mmol) and benzyl isocyanide (0.029 mL, 0.24 mmol) were added and the vial sealed. The solution was heated in a 2.45 GHz microwave reactor targeting 45 °C for 45 mins before being concentrated *in vacuo*. The residue was redissolved in EtOAc (5 mL) and washed with brine (3 x 5 mL). The organic phase was dried over $MgSO_4$, filtered and concentrated *in vacuo* to give the crude product which was purified by flash column chromatography (SiO_2, 80% EtOAc in hexane) to give **23** as a white crystalline solid (0.088 g, 0.14 mmol, 58%): m.p. ($CHCl_3$): 178-179 °C; ^{1}H NMR (500 MHz, $CDCl_3$) δ_H 7.38-7.09 (10H, overlapping m, ArH), 5.32-5.09 (1H, m, FCH-4), 5.14-4.96 (1H, m, CH_2CH-2), 4.88-4.75 (1H, m, FCH-2a and FCH-6a), 4.72-4.60 (2H, m, PhCH_2), 4.59-4.12 (7H, m, PhCH_2, FCH-2b, FCH-6b $(CH_3)_2$CHCH-2, FCH-3, FCH-5, CH_2CH-2), 2.58-2.37 (1h, m, CH_2-3a), 2.11-2.03 (1H, m, CH_2-3b), 1.94-1.87 (1H, m, $(CH_3)_2$CH-3), 1.91-1.76 (1H, m, FCCH-1), 1.43 (9H, s, $(CH_3)_3$), 0.93-0.79 (6H, m, $(CH_3)_2$-4); ^{19}F NMR (471 MHz, $CDCl_3$) δ_F −203.65, −211.55, −216.8; ^{13}C NMR (126 MHz, $CDCl_3$) δ_C 174.8 (*C*=O), 169.9 (*C*=O), 156.25 (*C*=O), 137.8 (ArC), 136.0 (ArC), 129.2 (Ar*C*H), 128.9 (Ar*C*H), 128.7 (Ar*C*H), 128.1 (Ar*C*H), 127.7 (Ar*C*H), 127.5 (Ar*C*H), 87.9 (*C*F), 86.7 (*C*F), 85.1 (*C*F), 80.3 ($(CH_3)_3$*C*), 56.4 ((CH$_3$)$_2$-4), 51.1 (Ph*C*H_2), 43.6 (Ph*C*H_2), 35.8 (FCC-1), 28.3 ((CH$_3$)$_3$), 25.8 (*C*H_2-3), 19.8 (H_3C-4a), 17.1 (H_3C-4b); HRMS m/z ESI$^+$ (Calculated $C_{33}H_{42}F_5N_3O_4Na^+$ = 662.2988) found 662.2984 $[M+Na]^+$; ν_{max}/cm^{-1} 2965br (C-H), 1672 and 1639 (C=O), 1136 and 1047 (C-F).

2-(N-Allyl-2,2,2-trifluoroacetamido)-N-benzyl-3-((*1r,2R,3R,4s,5S,6S*)-2,3,4,5,6-pentafluorocyclohexyl)propenamide 24

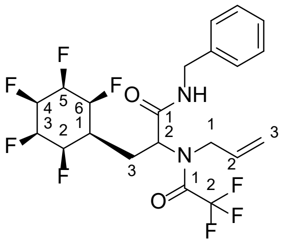

Allylamine (0.017 mL, 0.19 mmol) was added to a solution of **13** (0.050 g, 0.23 mmol) in MeOH (0.5 mL) in a microwave vial and the solution was stirred for 5 mins. Then, trifluoroacetic acid (0.015 mL, 0.19 mmol) and benzyl isocyanide (0.023 mL, 0.19 mmol) were added and the vial sealed. The solution was heated in a 2.45 GHz microwave reactor targeting 45 °C for 45 mins before being concentrated *in vacuo*. The residue was redissolved in EtOAc (5 mL) and washed with brine (3 x 5 mL). The organic phase was dried over $MgSO_4$, filtered and concentrated *in vacuo* to give the crude product which was purified by flash column chromatography (SiO_2, 50% EtOAc in hexane) to give **24** as a white crystalline solid (0.061 g, 0.13 mmol, 68%): m.p. (MeOH): 141-142 °C ^{1}H NMR (400 MHz, Methanol-*d*$_4$) δ_H 7.34-7.23 (5H, overlapping m, ArH), 5.92-5.78 (1H, m, C=C-H-2), 5.34-4.50 (8H, overlapping m, FCH-2, FCH-3, FCH-4, FCH-5, FCH-6, NCH_2CHCH_2-3, NCH-2), 4.40-4.31 (2H, m, PhCH_2), 4.27-4.13 (2H, m, NCH_2-1), 2.61-2.51 (1H, m, $FCHCHCH_2$-3a), 2.29-2.16 (1H, m, $FCHCHCH_2$-3b), 1.95-1.73 (1H, m, FCHCH-1); ^{19}F NMR (377 MHz, Methanol-*d*$_4$) δ_F −70.1 (CF_3), −206.0, −212.6, −213.1, −218.1; ^{13}C NMR (126 MHz, Methanol-*d*$_4$) δ 168.9 (C=O), 157.8 (C=O), 138.2 (ArC), 133.1 (C=C-2), 128.2 (ArH), 127.3 (ArH), 126.9 (ArH), 118.5 (C=C-3), 87.6 (*C*F), 87.5 (*C*F), 86.2 (*C*F), 57.0 (N*C*H-2), 49.0 (N*C*H_2-1) 43.0 (Ph*C*H_2), 35.3 (FCH*C*H-1), 25.5 (FCHCH*C*H_2-3); HRMS m/z ESI$^+$ (Calculated $C_{21}H_{22}F_8O_2N_2Na^+$ = 509.1446) found 509.1442 $[M+Na]^+$; ν_{max}/cm^{-1} 1682 (C=O), 1150 and 1136 (C-F).

N-Benzyl-N-(1-(benzylamino)-1-oxo-3-((*1r,2R,3R,4s,5S,6S*)-2,3,4,5,6-pentafluorocyclohexyl)propan-2-yl)propiolamide 25

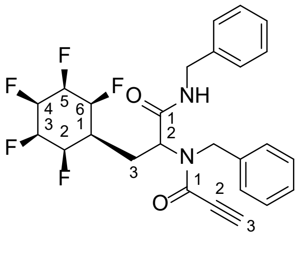

Benzylamine (0.025 mL, 0.23 mmol) was added to a solution of **13** (0.050 g, 0.23 mmol) in MeOH (0.5 mL) in a microwave vial and the solution was stirred for 5 mins. Then propiolic acid (0.012 mL, 0.23 mL) and benzyl isocyanide (0.023 mL, 0.19 mmol) were added and the vial sealed. The solution was heated in a 2.45 GHz microwave reactor targeting 45 °C for 45 mins before being concentrated *in vacuo*. The residue was redissolved in EtOAc (5 mL) and washed with brine (3 x 5 mL). The organic phase was dried over $MgSO_4$, filtered and concentrated *in vacuo* to give the crude product which was purified by flash column chromatography (SiO_2, 50% EtOAc in hexane to 70% EtOAc in hexane) to give **25** as a white crystalline solid (0.043 g, 0.087 mmol, 46%): m.p. (Acetone): 186-187 °C; Rotamers are present and observable in NMR spectra: ^{1}H NMR (500 MHz, Acetone-*d*$_6$) δ_H 8.09 and 7.86 (1H, s, NH), 7.59-7.24 (10H, overlapping m, ArH), 5.39-5.35 and 5.19-5.12 (1H, m, NCH-2), 5.29-3.80 (10H, overlapping m, FCH-2, FCH-3, FCH-4, FCH-5, FCH-6, $PhCH_2$, C≡CH-3), 2.52-1.94 (2H, overlapping m (rotamers), $FCCCH_2$-3), 1.79-1.63 (1H, m, FCCH-1); ^{19}F NMR (470 MHz, Acetone-*d*$_6$) δ_F −205.2, −212.4, −217.5; ^{13}C NMR (126 MHz, Acetone-*d*$_6$) δ_C 169.3 (C=O), 155.8 (C=O), 155.5 (C=O), 140.0 (ArC), 139.6 (ArC), 138.6 (ArC), 129.5 (ArCH), 129.35 (ArCH), 129.2 (ArCH), 128.9 (ArCH), 128.8 (ArCH), 128.55 (ArCH), 128.5 (ArCH), 128.2 (ArCH), 127.9 (ArCH), 127.8 (ArCH), 89.5 (*C*F), 88.6 (*C*F), 87.4 (*C*F), 82.8 (C≡C-2a), 82.0 (C≡C-2b), 60.5 (N*C*H-2a), 55.3 (N*C*H-2b), 51.45, 46.9, 43.9 (Ph*C*H$_2$), 43.8 (Ph*C*H$_2$), 35.8 (FCC-1), 27.5 (FCCC-3a), 26.9 (FCCC-3b); HRMS m/z ESI$^+$ (Calculated $C_{26}H_{25}F_5NO_2Na^+$ = 515.1728) found 515.1708; ν_{max}/cm^{-1} 2106 (C≡C), 1620 (C=O), 1134 and 1049 (C-F).

2-(N-Allylacetamido)-N-benzyl-3-phenylpropanamide 26

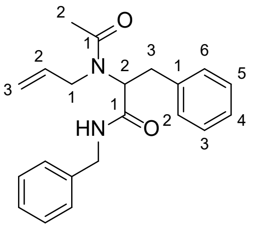

Allylamine (0.034 mL, 0.46 mmol) was added to a solution of phenylacetaldehyde (0.055 g, 0.46 mmol) in MeOH (1 mL) in a microwave vial and the solution was stirred for 5 mins. Then, acetic acid (0.022 mL, 0.38 mmol) and benzyl isocyanide (0.046 mL, 0.38 mmol) were added and the vial sealed. The solution was heated in a 2.45 GHz microwave reactor targeting 45 °C for 45 mins before being concentrated *in vacuo*. The residue was redissolved in EtOAc (5 mL) and washed with brine (3 x 5 mL). The organic phase was dried over $MgSO_4$, filtered and concentrated *in vacuo* to give the crude product which was purified by flash column chromatography (SiO_2, 30% EtOAc in hexane to 100% EtOAc in hexane) to give **26** as a colourless oil (0.060 g, 0.18 mmol, 47%): ^{1}H NMR (500 MHz, $CDCl_3$) δ_H 7.31-7.21 (8H, overlapping m, ArH), 7.09-7.06 (2H, m, ArH), 6.80 (1H, t J = 5.5 Hz, NH), 5.70-5.62 (1H, m, C=CH-2), 5.20-5.12 (3H, overlapping m, C=CH_2-3 and NCH-2), 4.42 (1H, dd J = 15.0, 6.3 Hz, $PhCH_2$-a), 4.28 (1H, dd J = 15.0, 5.6 Hz, $PhCH_2$-b), 4.02-3.93 (2H, m, NCH_2-1), 3.33 (1H, dd J = 13.8, 8.9 Hz, $PhCH_2$-3a), 3.06 (1H, dd J = 13.8, 7.0 Hz, $PhCH_2$-3b), 2.04 (3H, s, CH_3); ^{13}C NMR (126 MHz, $CDCl_3$) δ_C 172.7 (*C*=O), 170.1 (*C*=O), 138.05 (ArC), 137.2 (ArC), 133.85 (C=C-2), 129.3 (Ar*C*H), 128.6 (Ar*C*H), 127.6 (Ar*C*H), 127.3 (Ar*C*H), 126.75 (Ar*C*H), 117.1 (C=C-3), 58.9 (N*C*H-2), 48.65 (N*C*H$_2$-1), 43.3 (Ph*C*H$_2$), 34.7 (Ph*C*H$_2$-3), 22.1 (H_3C-2); HRMS m/z ESI$^+$ (Calculated $C_{21}H_{24}N_2O_2Na^+$ = 359.1730) found 359.1723 [M+Na]$^+$; ν_{max}/cm^{-1} 1624 (C=O), 1410 (C=C Ar).

2-(N-Allyl-2-(2-chlorophenyl)acetamido)-N-benzyl-3-phenylpropanamide 27

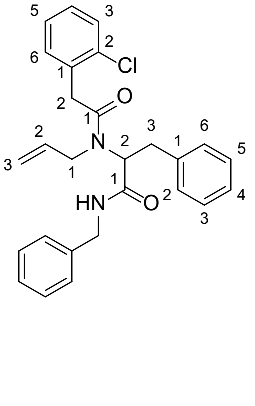

Benzylamine (0.025 mL, 0.23 mmol) was added to a solution of phenylacetaldehyde (0.025 mL, 0.23 mmol) in MeOH (0.5 mL) in a microwave vial and the solution was stirred for 5 mins. Then, acetic acid (0.011 mL, 0.19 mmol) and benzyl isocyanide (0.023 mL, 0.19 mmol) were added and the vial sealed. The solution was heated in a 2.45 GHz microwave reactor targeting 45 °C for 45 mins before being concentrated *in vacuo*. The residue was redissolved in EtOAc (5 mL) and washed with brine (3 x 5 mL). The organic phase was dried over $MgSO_4$, filtered and concentrated *in vacuo* to give the crude product which was purified by flash column chromatography (SiO_2, 50% EtOAc in hexane to 70% EtOAc in hexane) to give **27** as a colourless oil (0.030 g, 0.067 mmol, 35%): ^{1}H NMR (500 MHz, $CDCl_3$) δ_H 7.35-7.05 (14H, overlapping m, ArH), 6.75 (1H, app t *J* 5.5 Hz, NH), 5.70-5.61 (1H, m, C=CH-2), 5.21-5.12 (3H, overlapping m, NCH-2 and C=CH_2-3), 4.36 (1H, dd *J* = 14.9, 6.1 Hz, $PhCH_2$a), 4.30 (1H, dd *J* = 14.9, 5.8 Hz, $PhCH_2$b), 4.05 (1H, app ddt *J* = 17.9, 5.0, 1.7 Hz, NCH_2-1a), 3.99 (1H, app ddt *J* = 17.9, 5.5, 1.6 Hz, NCH_2-1b), 3.71 (2H, s, $OCCH_2$-2), 3.34 (1H, dd *J* = 14.0, 8.2 Hz, $PhCH_2$-3a), 3.13 (1H, dd *J* = 14.0, 7.8 Hz, $PhCH_2$-3b); ^{13}C NMR (126 MHz, $CDCl_3$) δ_C 172.1 (C=O), 170.0 (C=O), 138.0 (ArC), 137.1 (ArC), 134.2 (ArC), 133.55 (C=C-2), 133.2 (ArC), 129.4 (ArCH), 129.3 (ArCH), 128.6 (ArCH), 128.6 (ArCH), 128.55 (ArCH), 127.6 (ArCH), 127.3 (ArCH), 127.0 (ArCH), 126.7 (ArCH), 117.5 (C=C-3), 59.4 (N*C*H-2), 48.35 (N*C*H_2-1), 43.3 (Ph*C*H_2), 38.8 (OC*C*H_2-2), 34.4 (Ph*C*H_2-3); HRMS m/z ESI$^+$ (Calculated $C_{27}H_{27}O_2N_2ClNa^+$ = 469.1653) found 469.1643; ν_{max}/cm^{-1} 1636 (C=O), 1454 (C=C Ar).

N-Benzyl-2-(N-benzylacetamido)-3-phenylpropanamide 28

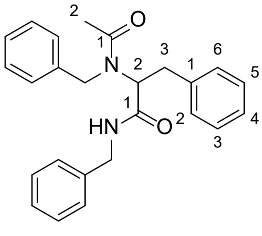

Benzylamine (0.025 mL, 0.23 mmol) was added to a solution of phenylacetaldehyde (0.025 mL, 0.23 mmol) in MeOH (0.5 mL) in a microwave vial and the solution was stirred for 5 mins. Then, acetic acid (0.011 mL, 0.19 mmol) and benzyl isocyanide (0.023 mL, 0.19 mmol) were added and the vial sealed. The solution was heated in a 2.45 GHz microwave reactor targeting 45 °C for 45 mins before being concentrated *in vacuo*. The residue was redissolved in EtOAc (5 mL) and washed with brine (3 x 5 mL). The organic phase was dried over $MgSO_4$, filtered and concentrated *in vacuo* to give the crude product which was purified by flash column chromatography (SiO_2, 50% EtOAc in hexane to 70% EtOAc in hexane) to give **28** as a colourless oil (0.030 g, 0.078 mmol, 35%): ^{1}H NMR (500 MHz, $CDCl_3$) δ_H 7.30-7.01 (15H, overlapping m, ArH), 6.68 (1H, app t *J* = 5.5 Hz, NH), 5.12 (1H, dd *J* = 9.0, 6.8 Hz, NCH-2), 4.61 (1H, d *J* = 17.7, $PhCH_2$a) 4.55 (1H, d *J* = 17.7, $PhCH_2$b), 4.41 (1H, dd *J* =14.9, 6.4 Hz, $PhCH_2$a'), 4.18 (1H, dd, *J* = 14.9, 5.4 Hz, $PHCH_2$-b'), 3.25 (1H, dd *J* = 13.6, 9.0 Hz, $PhCH_2$-3a), 3.00 (1H, dd *J* = 13.6, 6.8, $PhCH_2$-3b), 2.04 (3H, s, CH_3-2); ^{13}C NMR (126 MHz, $CDCl_3$) δ_C 173.0 (*C*=O), 170.0 (*C*=O), 138.0 (ArC), 137.3 (ArC), 129.4 (ArCH), 129.0 (ArCH), 128.7 (ArCH), 127.6 (ArCH), 127.5 (ArCH), 127.4 (ArCH), 126.8 (ArCH), 126.2 (ArCH), 60.0 (N*C*H-2), 50.2 (Ph*C*H_2), 43.3 (Ph*C*H_2), 34.9 (Ph*C*H_2-3), 22.5 (H_3C-2); HRMS m/z ESI$^+$ (Calculated $C_{25}H_{26}O_2N_2Na^+$ = 409.1886) found 409.1875 $[M+Na]^+$; ν_{max}/cm^{-1} 1626 (C=O), 1452 (C=C Ar).

(*1r,2R,3R,4s,5S,6S*)-1-(2-Bromoethyl)-2,3,4,5,6-pentafluorocyclohexane 29

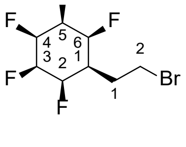

Ph_3P (0.278 g, 1.06 mmol) and CBr_4 (0.352 g, 1.06 mmol) were added to a solution of **19** (0.1155 g, 0.5294 mmol) in CH_3CN (5 mL) at r.t. for 16 h. The reaction mixture was concentrated *in vacuo* and purified directly by flash column chromatography (SiO_2, hexane to 40% EtOAc in hexane) to give **29** as a white crystalline solid (0.125 g, 0.445 mmol, 84%): m.p. (MeOH): 154-155 °C; ^{1}H NMR (400 MHz, Methanol-*d*$_4$) δ_H 5.37-5.17 (1H, m, FCH-4), 5.06-4.88 (2H, m, FCH-2 and FCH-6), 4.83-4.54 (2H, m, FCH-3 and FCH-5), 3.65 (2H, t *J* = 6.7 Hz, CH_2-2), 2.32 (2H, m, CH_2-1), 2.13 (1H, m, FCCH-1); ^{19}F NMR (377 MHz, Methanol-*d*$_4$) δ_F -206.1, -213.0, -218.3; ^{13}C NMR (101

MHz, Methanol-d_4) δ_C 89.7 (FC-4), 88.5 (FC-2 and FC-6), 87.6 (FC-3 and FC-5), 37.9 (FCC-1), 31.0 (H_2C-2), 30.2 (H_2C-1); HRMS m/z (ESI$^+$) (calculated $C_8H_{10}F_5BrNa^+$ = 302.9778) found 302.9782 [M+Na]$^+$; ν_{max}/cm^{-1} 2900 br (C-H).

(*1r,2R,3R,4s,5S,6S*)-1-(2-Azidoethyl)-2,3,4,5,6-pentafluorocyclohexane 14

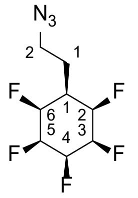

To a solution of **29** (0.441 g, 1.57 mmol) in DMF (10 mL) was added sodium azide (0.201 g, 3.09 mmol). The solution was warmed to 70 °C and stirred for for 6 h until TLC showed complete consumption of the starting material. Water (5 mL) was added, and the mixture extracted into EtOAc (3 x 20 mL). The combined organic phase was washed with brine, dried over $MgSO_4$, filtered and concentrated *in vacuo* to give the crude product which was purified by flash column chromatography (SiO_2, 40% EtOAc in hexane) to give **14** as a white crystalline solid (0.381 g, 1.57 mmol, quantitative): m.p. (MeOH): 102-103 °C; ^{1}H NMR (500 MHz, Methanol-d_4) δ_H 5.36-5.17 (1H, m, H-4), 5.00-4.88 (2H, m, H-2 and H-6), 4.78-4.55 (2H, m, H-3 and H-5), 3.54 (2H, t *J* = 6.5, CH_2-2), 2.08-1.88 (3H, overlapping m, CH_2-1 and FCCH-1); ^{19}F NMR (471 MHz, Methanol-d_4) δ_F -205.9, -213.1, -218.2; ^{13}C NMR (126 MHz, Methanol-d_4) δ_C 89.8 (FC-4), 88.4 (FC-2 and FC-6), 87.1 (FC-3 and FC-5), 49.4 (H_2C-2), 36.8 (FCC-1), 26.4 (H_2C-1); HRMS m/z (ESI$^+$) (Calculated $C_8H_{10}F_5N_3Na$ = 266.0693) found 266.0682 [M+Na]$^+$; ν_{max}/cm^{-1} 2976 and 2951 (C-H), 2102 (N=N=N).

2-((*1r,2R,3R,4s,5S,6S*)-2,3,4,5,6-Pentafluorocyclohexyl)ethan-1-amine 34

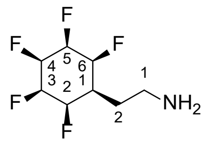

Ph_3P (147 mg, 0.560 mmol) was added to a solution of **14** (0.068 g, 0.28 mmol) in THF (1 mL). The solution was stirred at r.t. for 2h and then water (0.1 mL) was added. The mixture was stirred for a further 22 h before being quenched by addition of HCl (2M, 0.25 mL). The aqueous layer was washed with EtOAc (3 x 1 mL) before being basified with NaOH (2M) to pH 2. The basic aqueous phase was extracted into EtOAc (3 x 5 mL) and this organic phase was washed with brine (2 x 5 mL), dried over $MgSO_4$, filtered and concentrated *in vacuo* to give **34** (40 mg, 0.18 mmol, 64%) as a crystalline white solid: m.p. (EtOAc) 141-142 °C; ^{1}H NMR (500 MHz, Methanol-d_4) δ_H 5.37-5.19 (1H, m, FCH-4), 4.98-4.87 (2H, m, FCH-2 and FCH-6), 4.75-4.55 (2H, m, FCH-3 and FCH-5), 2.81 (2H, t *J* = 6.9 Hz, CH_2-1), 1.99-1.82 (3H, overlapping m, FCCH-1 and CH_2-2); ^{19}F NMR (471 MHz, Methanol-d_4) δ_F -205.8, -213.5, -218.2; ^{13}C NMR (126 MHz, Methanol-d_4) δ_C 89.9 (FC), 88.5 (FC), 87.1 (FC-3 and FC-5), 39.2 (H_2C-1), 36.6 (FCC-1), 29.9 (H_2C-2); HRMS m/z (ESI$^+$) (Calculated $C_8H_{13}F_5N^+$ = 218.0963) found 218.0958 [M+H]$^+$; ν_{max}/cm^{-1} 3354 br (N-H), 2955 (C-H), 1576 (N-H), 1124 and 1047 (C-N).

1-(2-((*1r,2R,3R,4s,5S,6S*)-2,3,4,5,6-Pentafluorocyclohexyl)ethyl)-4-(p-tolyl)-1H-1,2,3-triazole 30

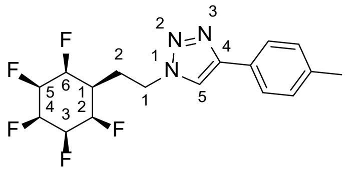

4-Ethynyltoluene (0.034 g, 0.29 mmol), sodium ascorbate (0.008 g, 0.04 mmol) and copper sulfate pentahydrate (0.002 g, 0.008 mmol) were added to a solution of **14** (0.071 g, 0.29 mmol) in ethanol (1 mL) and water (1 mL). The solution was warmed to 65 °C and stirred for 4 h before being extracted into EtOAc (3 x 5 mL). The combined

organic phase was washed with brine (5 mL), dried over $MgSO_4$, filtered and concentrated *in vacuo*. The crude product was purified by flash column chromatography (SiO_2, 50% EtOAc in hexane) to give **30** (0.090 g, 0.25 mmol, 86%) as a white powder: m.p. (Acetone): 230-231 °C; ^{1}H NMR (500 MHz, Acetone-d_6) δ_H 8.40 (1H, s, C=CH-5, 7.77-7.75 (2H, m, ArH), 7.26-7.24 (2H, m, ArH), 5.50-5.32 (1H, m, FCH-4), 5.26-5.10 (2H, m, FCH-2 and FCH-6), 5.03-4.83 (2H, m, FCH-3 and FCH-5), 4.72 (2H, t *J* = 6.8 Hz, NCH_2-1), 2.51-2.47 (2H, m, $FCCCH_2$-2), 2.35 (3H, s, CH_3), 2.18-2.05 (1H, m, FCCH-1); ^{19}F NMR (470 MHz, Acetone-d_6) δ_F −205.2, −212.8, −217.5; ^{13}C NMR (126 MHz, Acetone-d_6) δ_C 148.1 (C=C-4), 138.3 (ArC), 130.23 (Ar*C*H), 129.5 (ArC), 126.14 (Ar*C*H), 121.2 (C=C-5), 88.5 (*C*F), 88.4 (*C*F), 87.3 (*C*F), 47.5 (N*C*H_2-1), 36.1 (FCC-1), 27.6 (FCC*C*H_2-2), 21.2 (H_3C); HRMS m/z ESI$^+$ (Calculated $C_{17}H_{19}F_5N_3^+$ = 360.1494) found 360.1486 [M+H]$^+$; ν_{max}/cm^{-1} 1520 (C=C Ar).

4-([1,1'-Biphenyl]-4-yl)-1-(2-((*1r,2R,3R,4s,5S,6S*)-2,3,4,5,6-pentafluorocyclohexyl)ethyl)-1H-1,2,3-triazole 31

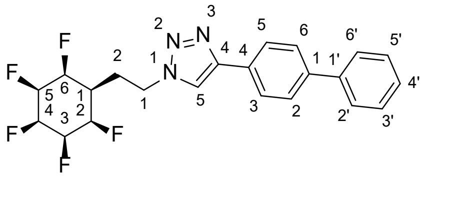

4-Ethynylbenzene (0.067 g, 0.37 mmol), sodium ascorbate (0.034 g, 0.17 mmol) and copper sulfate pentahydrate (0.008 g, 0.03 mmol) were added to a solution of **14** (0.076 g, 0.31 mmol) in EtOH (1 mL) and water (1 mL). The solution was heated to 65 °C and stirred for 16 h before being extracted into EtOAc (3 x 5 mL). The combined organic phase was washed with brine (5 mL), dried over $MgSO_4$, filtered, and concentrated *in vacuo*. The crude product was purified by flash column chromatography (SiO_2, 70% EtOAc in hexane) to give **31** as a white crystalline solid (0.083 g, 0.20 mmol, 65%): m.p. (acetone): 267-268 °C; ^{1}H NMR (700 MHz, Acetone-d_6) δ_H 8.51 (1H, s, C=CH-5), 7.99-7.97 (2H, m, ArCH), 7.76-7.74 (2H, m, ArCH), 7.72-7.70 (2H, m, ArCH), 7.49-7.46 (2H, m, ArCH), 7.39-7.36 (1H, m, ArCH), 5.45-5.38 (1H, m, FCH-4), 5.23-5.16 (2H, m, FCH-2 and FCH-6), 5.00-4.88 (2H, m, FCH-3 and FCH-5), 4.76 (2H, t *J* = 6.8 Hz, NCH_2-1), 2.53-2.50 (2H, m, $FCCCH_2$-2), 2.17-2.06 (1H, m, FCCH-1); ^{19}F NMR (659 MHz, Acetone-d_6) δ_F −205.2, −212.75, −217.5; ^{13}C NMR (176 MHz, Acetone-d_6) δ_C 147.7 (C=C-4), 141.3 (ArC), 141.2 (ArC), 131.3 (ArC), 129.8 (ArCH), 128.3 (ArCH), 128.1 (ArCH), 127.6 (ArCH), 126.7 (ArCH), 121.7 (C=CH-5), 88.95 (*C*F), 87.9 (*C*F), 86.9 (*C*F), 47.6 (N*C*H_2-1), 36.1 (FCC-1), 27.6 (FCCC-2); HRMS m/z ESI$^+$ (Calculated $C_{22}H_{21}F_5N_3^+$ = 422.1650) found 422.1641 [M+H]$^+$; ν_{max}/cm^{-1} 1693 (C=C), 1485 (C=C Ar), 1130 and 1049 (C-F).

4-(1-(2-((*1r,2R,3R,4s,5S,6S*)-2,3,4,5,6-Pentafluorocyclohexyl)ethyl)-1H-1,2,3-triazol-4-yl)benzoic acid 32

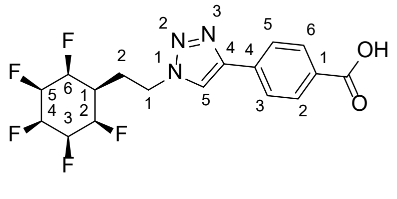

Sodium ascorbate (0.017 g, 0.086 mmol) and copper sulfate pentahydrate (0.021 g, 0.086 mmol), ^{i}PrNEt$_2$ (0.060 mL, 0.34 mmol) and **14** (0.050 g, 0.21 mmol) were added to a suspension of 4-ethynylbenzoic acid (0.025 g, 0.17 mmol) in MeOH (1 mL) and water (1 mL) The solution was heated to 65 °C and stirred for 16 h. After cooling to r.t. the crude product was precipitated from solution by addition of HCl (1M, 1 mL). The precipitate was isolated by filtration and then partitioned between EtOAc (3 mL) and saturated $NaHCO_3$ (3 mL). The aqueous phase was acidified by addition of HCl (2M) and then extracted into EtOAc (3 x 10 mL). The combined organic phase was washed with brine (30 mL), dried over $MgSO_4$, filtered and concentrated *in vacuo* to give **32** as a white crystalline solid (0.042 g, 0.11 mmol, 63%): m.p. (Acetone): >300 °C (no melt observed); ^{1}H NMR (500 MHz, DMSO-d_6) δ_H 12.94 (1H, br s, CO_2H), 8.80 (1H, s, C=CH-5), 8.03-7.96 (4H, overlapping m, ArCH), 5.46-5.28 (1H, m, FCH-4), 5.19-4.77 (4H, overlapping m, FCH-2, FCH-3, FCH-5 and FCH-6), 4.63 (1H, t *J* = 7.1 Hz, NCH_2-1), 2.33-2.28 (2H, m, $FCCCH_2$-2), 2.04-1.83 (1H, m, FCCH-1); ^{19}F NMR (470 MHz, DMSO-d_6) δ_F −203.6, −211.6, −216.3; ^{13}C NMR (126 MHz, DMSO-d_6) δ_C 167.0 (*C*=O), 145.5 (C=C-4), 134.9 (ArC), 130.0 (Ar*C*H), 129.85 (ArC), 125.05 (Ar*C*H), 122.8 (C=C-5), 88.1 (*C*F), 86.6 (*C*F), 85.45 (*C*F), 46.6 (N*C*H_2-1), 34.5 (FCC-1), 26.25 (FCC*C*H_2-2); HRMS m/z ESI$^+$ (Calculated

$C_{17}H_{15}F_5N_3O_2^-$ = 388.1090) found 388.1086 [M−H]$^-$; ν_{max}/cm^{-1} 1670 (C=O), 1427 (C=C Ar), 1132, 1103 and 1053 (C-F).

1,4-Bis(1-(2-((*1r,2R,3R,4s,5S,6S*)-2,3,4,5,6-pentafluorocyclohexyl)ethyl)-1H-1,2,3-triazol-4-yl)benzene 33

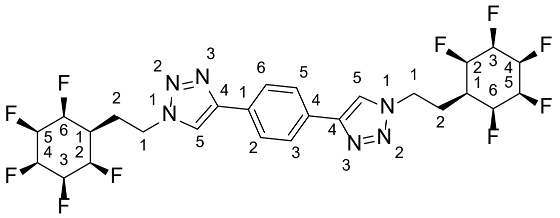

Sodium ascorbate (0.008 g, 0.04 mmol) and copper sulfate pentahydrate (2 mg, 0.008 mmol) were added to a solution of **14** (0.050 g, 0.21 mmol) and 1,4-diethynylbenzene (0.013 g, 0.10 mmol) in ^{t}BuOH (3 mL) and water (3 mL). The solution was heated to 80 °C for 3 h. After cooling to r.t. a white precipate was isolated by filtration and washed with cold Et_2O (5 mL) to give **33** as a fine white powder (0.051 g, 0.083 mmol, 83%): m.p. (Acetone): >300 °C (no melt); ^{1}H NMR (500 MHz, DMSO-*d*$_6$) δ_H 8.70 (2H, s, C=CH-5), 7.93 (4H, s, ArCH), 5.44-5.29 (2H, m, FCH-4), 5.18-5.05 (4H, m, FCH-2 and FCH-6), 5.00-4.78 (4H, m, FCH-3 and FCH-5), 4.61 (4H, t *J* = 7.1 Hz, NCH_2-1), 2.32-2.28 (4H, m, $FCCCH_2$-2), 2.06-1.86 (2H, m, FCCH-1); ^{19}F NMR (471 MHz, DMSO-*d*$_6$) δ_F −203.7, −211.6, −216.3; ^{13}C NMR (126 MHz, DMSO-*d*$_6$) δ_C 146.1 (C=C-4), 130.2 (ArC), 125.6 (Ar*C*H), 121.7 (C=C-5), 88.2 (*C*F), 86.8 (*C*F), 85.5 (*C*F), 46.55 (N*C*H_2-1), 34.5 (FCC-1), 26.3 (FCC*C*H_2-2); HRMS m/z ESI$^+$ (Calculated $C_{26}H_{27}F_{10}N_6^+$ = 613.2132) found 613.2122 [M+H]$^+$; ν_{max}/cm^{-1} 1132 (C-F).

Methyl (*S*)-2-((*tert*-butoxycarbonyl)amino)-3-(perfluorophenyl)propanoate 36

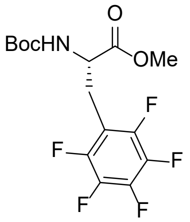

To a solution of (*S*)-2-((*tert*-butoxycarbonyl)amino)-3-(perfluorophenyl)propanoic acid (1.04 g, 3.0 mmol, 1.0 equiv.) in a mixture of toluene:methanol (12:12 mL), added TMS-diazomethane (6.0 mL (2M in hexane), 12.0 mmol, 4.0 equiv.) at 0 °C and stirred at r.t.. Upon completion (aprox. 2 hr), the reaction was concentrated *in vacuo* and purified by silica gel column chromatography using 0-10% EtOAc in petroleum ether to afford methyl (*S*)-2-((*tert*-butoxycarbonyl)amino)-3-(perfluorophenyl)propanoate **36** as a white solid (1.08 g, quant.). ^{1}H NMR (400 MHz, Methanol-*d*$_4$) δ: 4.41 (dd, *J* = 9.2, 5.3 Hz, 1H), 3.75 (s, 3H), 3.33 – 3.22 (m, 1H), 3.11 (dd, *J* = 14.1, 9.2 Hz, 1H), 1.37 (s, 9H). Data in accordance with literature[3, 4]

(*S*)-2-((tert-Butoxycarbonyl)amino)-3-((*1r,2R,3R,4R,5S,6S*)-2,3,4,5,6-pentafluorocyclohexyl)propanoic acid 37

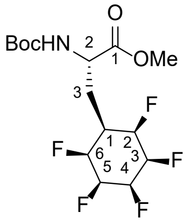

To a solution of methyl (*S*)-2-((*tert*-butoxycarbonyl)amino)-3-(perfluorophenyl)propanoate **36** (500 mg, 1.33 mmol) and **12** (2 mol%) in dry hexane (10 mL) in a glass vial under argon atmosphere, added oven dried 4Å molecular sieves (400 mg). The glass vial was placed in a 150 mL stainless steel autoclave under argon atmosphere. The autoclave was pressurized (40-45 bar) and depressurized with hydrogen gas three times before keeping the reaction under 50 bar hydrogen gas pressure and stirred at r.t. for 3 days. After the autoclave was carefully depressurized,

filtered through celite and washed with a solution of 3% MeOH in CH_2Cl_2 (10 mL x 3). Filtrate was concentrated and the residue was purified by silica gel column chromatography (1-2% MeOH in CH_2Cl_2) to afford **37** as a white solid (254 mg, 50%): m.p. 170-172 °C; $[\alpha]_D^{20}$ = −22.5 (c = 1.0, MeOH); ^{1}H NMR (400 MHz, Methanol-d_4) δ: 5.27 (m, 1H), 5.10-4.50 (m, 5H), 4.30 (dd, *J* = 10.6, 4.6 Hz, 1H), 3.75 (s, 3H), 2.42 (m, 1H), 2.11 – 1.83 (m, 2H), 1.44 (s, 9H).^{13}C NMR (126 MHz, Methanol-d_4) δ: 172.9 (*C*=O), 156.9 (*C*=O), 89.5-85.2 (5 x *C*HF), 79.5 (*C*), 51.5 (*C*H_3), 50.9 (*C*H), 35.3-34.9 (m, 5x *C*HF), 27.8 (*C*H_2), 27.2 (3 x *C*H_3).^{19}F NMR (376 MHz, Methanol-d_4) δ: -206.1 (m, 2 x CH*F*), -212.4 (m, CH*F*), -214.0 (m, CH*F*), -218.28 (tt, *J* = 25.6, 10.7 Hz, CH*F*); HRMS (ESI$^+$) $C_{15}H_{22}O_4NF_5Na$ $[M+Na]^+$ found 398.1353, requires 398.1361; ν_{max}/cm^{-1} 1722 (C=O), 1487 (C-H), 1234 (C-H), 867 (C-F).

(S)-2-Amino-3-((*1r,2R,3R,4R,5S,6S*)-2,3,4,5,6-pentafluorocyclohexyl)propanoic acid 15

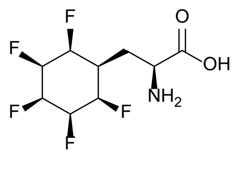

Compound **37** (210 mg, 0.56 mmol) was dissolved in 6 M HCl, heated under reflux for 12 hours. The water and HCl was removed *in vacuo*. The product was recrystalised in MeOH to afford **15** as a crystalline solid; m.p.: 272-274 °C (decomposition);), $[\alpha]_D^{20}$ = +20.7 (c = 0.67, MeOH); ^{1}H NMR (700 MHz, DMSO-d_6) δ: 5.37 (d, *J*= 54.3 Hz, C*H*F), 5.12-4.17 (m, 5H), 4.10 (1H, m, overlap with solvent), (2.30-2.21 ,2H) , ^{13}C NMR (176 MHz, DMSO-d_6) δ 171.0 (*C*OOH), 88.8-85.9 (*C*HF), 49.8 (*C*NH), 34.2 (CH), 27.2 (*C*H_2); ^{19}F NMR (659 MHz, DMSO-d_6) δ -203.6 (2F), -211.9, -212.8, -216.5; ν_{max}/cm^{-1} 1736 (C=O), 1717 (C-O), 1501 (N-H), 1229 (C-H), 1128 (C-O),1049 (C-N), 796 (C-F).

(2*S*)-2-((*tert*-Butoxycarbonyl)amino)-3-*cis-(*2,3,4,5,6-pentafluorocyclohexyl)propanoic acid 38

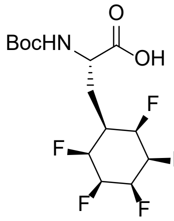

A suspension of methyl (2*S*)-2-((tert-butoxycarbonyl)amino)-3-(2,3,4,5,6-pentafluorocyclohexyl)propanoate **37** (100 mg, 0.27 mmol, 1.0 equiv.) in 6N HCl (10 mL) was stirred at reflux for 6 h. Upon completion, the reaction mass to dryness to afford crude (2*S*)-2-amino-3-(2,3,4,5,6-pentafluorocyclohexyl)propanoicacid.HCl as a white solid (quant.). Dried under vacuum for 2 hr. Dissolved in THF:Water (7:3 mL), added triethylamine (93 µL, 0.67 mmol, 2.5 equiv.) followed by di-*tert*-butyl dicarbonate (74 µL, 0.32 mmol, 1.2 equiv.) and stirred at r.t. for 4 h. Upon completion evaporated the solvent and extracted with EtOAc (10 mL x 3), dried (anhyd.Na_2SO_4), filtered and evaporated under reduced pressure. Residue was washed with diethylether:pentane (2 mL:2 mL) to get the pure (2*S*)-2-((*tert*-butoxycarbonyl)amino)-3-(2,3,4,5,6-pentafluorocyclohexyl)propanoic acid **38** as a white solid (75 mg, 78%); m.p.: 255-257 °C; $[\alpha]_D^{20}$ = −13.65 (c = 0.67, MeOH); ^{1}H NMR (400 MHz, Methanol-d_4) δ: 5.27 (m, 1H), 5.11-4.51 (m, 4H), 4.26 (dd, *J* = 10.6, 4.5 Hz, 1H), 2.51 – 2.36 (m, 1H), 2.10-1.83 (m, 2H), 1.45 (s, 9H).^{13}C NMR (126 MHz, Methanol-d_4) δ: 175.5 (*C*=O), 158.3 (*C*=O), 90.9-86.7 (m, 5 x *C*HF), 80.8 (*C*), 52.2 (*C*H), 37.02-36.2 (m, *C*H), 29.5 (*C*H_2), 28.7 (3 x *C*H_3). ^{19}F NMR (377 MHz, Methanol-d_4) δ: -206.1 (m, 2 x CH*F*), -212.5 (m, CH*F*), -214.0 (m, CH*F*), -218.3 (m, CH*F*). HRMS (ESI$^-$) $C_{14}H_{19}O_4NF_5$ $[M-H]^-$ found 360.1242, requires 360.1234; ν_{max}/cm^{-1} 1730 (C=O), 1699 (C-O), 1506 (C-C), 1242 (C-O), 796 (C-F).

Methyl (*S*)-2-((*S*)-2-((*tert*-butoxycarbonyl)amino)-3-((*1r,2R,3R,4R,5S,6S*)-2,3,4,5,6-pentafluorocyclohexyl)propanamido)-3-((*S*)-2-oxopyrrolidin-3-yl)propanoate 40

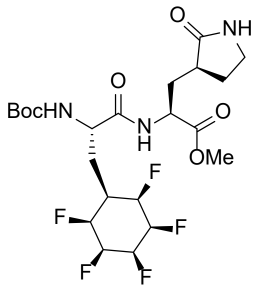

To a solution of methyl (*S*)-2-((*tert*-butoxycarbonyl)amino)-3-((*S*)-2-oxopyrrolidin-3-yl)propanoate **39** (77 mg, 0.27 mmol, 1.2 equiv.) in CH_2Cl_2 (5 mL) at 0 °C, added TFA (168 µL, 2.20 mmol, 10 equiv.) and stirred at r.t.. Upon completion of the reaction from TLC (aprox. 2 h), evaporated the solvent and dried under vacuum (to remove excess of TFA). Residue dissolved in DMF (2.0 mL). Added (2*S*)-2-((*tert*-butoxycarbonyl)amino)-3-(2,3,4,5,6-pentafluorocyclohexyl)propanoic acid **38** (80 mg, 0.22 mmol, 1.0 equiv.), DIPEA (152 µL, 0.88 mmol, 4.0 equiv.) followed by HATU (103 mg, 0.27 mmol, 1.2 equiv.) and stirred overnight at 20-25 °C. Upon completion of the reaction, Product was extracted with EtOAc (20 mL x 3), dried (anhydr. Na_2SO_4), filtered and evaporated under reduced pressure. The residue was purified by silica gel column chromatography (0-5% MeOH in DCM) to afford pure Methyl (*S*)-2-((*S*)-2-((*tert*-butoxycarbonyl)amino)-3-((1*r*,2*R*,3*R*,4*R*,5*S*,6*S*)-2,3,4,5,6-pentafluorocyclohexyl) propanamido)-3-((*S*)-2-oxopyrrolidin-3-yl)propanoate **40** as a white solid (105 mg, 90%): m.p.: 220-222°C; $[\alpha]_D^{20}$ = -8.2 (*c* 1.0, MeOH); ^{1}H NMR (400 MHz, Methanol-d_4) δ: 5.31 (m, C*H*), 5.18 – 4.93 (m, 2H), 4.86 – 4.50 (m, 3H), 4.21 (m, 1H), 3.75 (s, 3H), 3.41 – 3.25 (m, 2H), 2.70-2.56 (m, 1H), 2.41-2.29 (m, 1H), 2.29 – 2.13 (m, 2H), 2.13 – 1.94 (m, 2H), 1.94 – 1.75 (m, 2H), 1.45 (s, 9H); ^{13}C NMR (126 MHz, Methanol-d_4) δ: 181.8 (C=O), 174.8 (C=O), 173.9 (C=O), 157.7 (C=O), 90.6 – 86.6 (m, 5 x *C*H), 80.7 (*C*), 53.4 (*C*H), 53.0 (*C*H), 51.7 (*C*H), 41.5 (*C*H$_2$), 39.5 (*C*H), 36.6-36.0 (m, 5 x *C*HF), 33.7 (*C*H$_2$), 29.6 (*C*H$_2$), 28.7 (*C*H$_3$), 28.6 (*C*H$_2$); ^{19}F NMR (470 MHz, $CDCl_3$) δ: -204.19 (m, 2 x C*F*), -212.22 (m, C*F*), -212.43 (m, C*F*), -217.04 (m, C*F*); HRMS (ASAP$^+$) $C_{22}H_{33}N_3O_6Na$ $[M+Na]^+$ found 552.2097, requires 552.2103; ν_{max}/cm^{-1} 1697 (C=O), 1683 (C=O), 1670 (C=O), 1134 and 1047 (C-F).

Methyl (*S*)-2-((*S*)-2-cinnamamido-3-((*1r,2R,3R,4R,5S,6S*)-2,3,4,5,6-pentafluorocyclohexyl)propanamido)-3-((*S*)-2-oxopyrrolidin-3-yl)propanoate 43

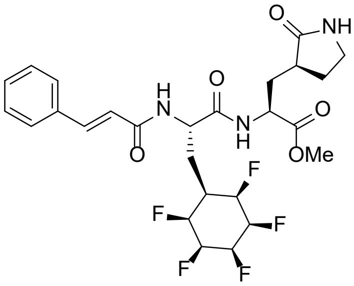

To a solution of methyl (*S*)-2-((*S*)-2-((*tert*-butoxycarbonyl)amino)-3-((1*r*,2*R*,3*R*,4*R*,5*S*,6*S*)-2,3,4,5,6-pentafluorocyclohexyl)propanamido)-3-((*S*)-2-oxopyrrolidin-3-yl)propanoate **40** (89 mg, 0.17 mmol, 1.0 equiv.) in MeOH (3 mL), added 4 N HCl in Dioxane (2.0 ml) and stirred at 20-25 °C. Upon completion of the reaction from TLC (aprox. 3 hr), evaporated the solvent and dried under vacuum. Residue dissolved in DMF (1.0 ml). To this added *E*-cinnamic acid (32 mg, 0.22 mmol, 1.3 equiv.), DIPEA (118 µL, 0.68 mmol, 4.0 equiv.) followed by HATU (71 mg, 0.19 mmol, 1.1 equiv.) at 20-25 °C and stirred for 16 hr. Upon completion of the reaction, Product was extracted with EtOAc (10 mL x 3), dried (anhydr.Na_2SO_4), filtered and evaporated under reduced pressure. The residue was purified by silica gel column chromatography (0-5% MeOH in DCM) to afford pure methyl (*S*)-2-((*S*)-2-cinnamamido-3-((1*r*,2*R*,3*R*,4*R*,5*S*,6*S*)-2,3,4,5,6-pentafluorocyclohexyl)propanamido)-3-((*S*)-2-oxopyrrolidin-3-yl)propanoate **43** as a white solid (83 mg, 88%); m.p.: 264-268 °C; $[\alpha]_D^{20}$ = -14.0 (*c* 0.5, MeOH); ν_{max}/cm^{-1} 1697 (C=O), 1647 (C=O); 1136 and 1049 (C-F); ^{1}H NMR (400 MHz, Methanol-d_4) δ: 7.60 – 7.51 (m, 3H), 7.43 – 7.34 (m, 3H), 6.68 (d, *J* = 15.8 Hz, 1H), 5.30 (m, 1H), 5.19-4.92 (m, 2H), 4.85 – 4.59 (m, 3H), 4.54 (dd, *J* = 11.7, 4.0 Hz, 1H), 3.74 (s, 3H), 3.37 – 3.25 (m, 2H), 2.67-2.56 (m, 1H), 2.40 – 2.26 (m, 2H), 2.24 – 2.14 (m, 2H), 2.13-2.92 (m, 1H), 1.91 – 1.73 (m, 2H); ^{13}C NMR (126 MHz, Acetone-d_6) δ: 179.6 (*C*=O), 173.2 (*C*=O), 172.2 (*C*=O), 166.2

(*C*=O), 141.0 (*C*H), 136.0 (*C*=O), 130.5 (*C*H), 129.8 (2 x *C*H), 128.6 (2 x *C*H), 122.2 (*C*H), 90.4 – 86.5 (m, 5 x *C*HF)), 52.5 (*C*H), 52.3 (CH_3), 51.0 (*C*H), 40.8 (CH_2), 40.6 (CH_2), 39.1 (*C*H), 35.9 (tt, *J* = 18.9, 6.0 Hz, *C*H), 33.5 (CH_2), 30.9 (CH_2); ^{19}F NMR (376 MHz, Methanol-d_4) δ: -205.94 (dt, *J* = 34.2, 11.2 Hz, 2 x C*F*), -213.11 (m, C*F*), -213.35 (m, C*F*), -218.30 (tt, *J* = 26.0, 10.6 Hz, C*F*); HRMS (ESI$^+$) $C_{26}H_{30}N_3O_5F_5Na$ $[M+Na]^+$ found 582.1990, requires 582.2003.

N-((*S*)-1-(((*S*)-1-Hydroxy-3-((*S*)-2-oxopyrrolidin-3-yl)propan-2-yl)amino)-1-oxo-3-((*1r,2R,3R,4R,5S,6S*)-2,3,4,5,6-pentafluorocyclohexyl)propan-2-yl)cinnamamide 44

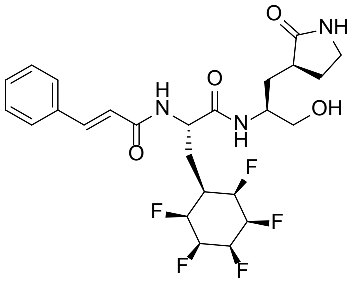

According to a literature procedure,[5] to a solution of methyl (*S*)-2-((*S*)-2-cinnamamido-3-((1*r*,2*R*,3*R*,4*R*,5*S*,6*S*)-2,3,4,5,6-pentafluorocyclohexyl)propanamido)-3-((*S*)-2-oxopyrrolidin-3-yl)propanoate (43) (50 mg, 0.09 mmol, 1.0 equiv.) in anhydrous MeOH (5.0 mL) was added sodium borohydride (68 mg, 1.79 mmol, 20 equiv.) slowly at 0 °C. Then, the reaction mixture was stirred at r.t. for 16 h. Upon completion, saturated NH_4Cl solution (20 mL) was added to quench the reaction. Evaporated methanol and extracted in EtOAc (10 mL × 3). The organic phase was washed with brine (5 mL), dried (anhyd. Na_2SO_4), filtered and concentrated under vacuum. The residue was purified by silica gel column chromatography (0-6 % MeOH in CH_2Cl_2) to afford pure N-((*S*)-1-(((*S*)-1-hydroxy-3-((*S*)-2-oxopyrrolidin-3-yl)propan-2-yl)amino)-1-oxo-3-((1*r*,2*R*,3*R*,4*R*,5*S*,6*S*)-2,3,4,5,6-pentafluorocyclohexyl)propan-2-yl)cinnamamide (44) as white solid (35 mg, 74%); m.p.: 194-196 °C; $[\alpha]_D^{20}$ = -20.0 (*c* 0.5, MeOH); ν_{max}/cm^{-1} 1653 (C=O), 1645 (C=O); ^{1}H NMR (500 MHz, Acetone-d_6) δ: 7.89 (d, *J* = 8.4 Hz, 1H), 7.83 (d, *J* = 8.4 Hz, 1H), 7.61 – 7.49 (m, 4H), 7.43 – 7.32 (m, 4H), 7.00 (s, 1H), 6.80 (d, *J* = 15.7 Hz, 1H), 5.48 – 4.63 (m, 6H), 4.16 (t, *J* = 5.7 Hz, 1H), 4.10 – 3.99 (m, 2H), 3.57 (h, *J* = 5.5 Hz, 2H), 3.30 – 3.17 (m, 3H), 2.49 – 2.26 (m, 4H), 2.24 – 2.10 (m, 1H), 2.03 – 1.94 (m, 1H), 1.74 (ddt, *J* = 12.4, 10.1, 8.9 Hz, 1H), 1.53 (ddd, *J* = 13.9, 9.6, 4.1 Hz, 1H); ^{13}C NMR (126 MHz, Acetone-d_6 with 5 drops of DMSO-d_6) δ: 180.0 (*C*=O), 172.1 (*C*=O), 166.1(*C*=O), 140.3 (*C*H), 136.0 (*C*), 130.2 (*C*H), 129.7 (2 x *C*H), 128.4 (2 x *C*H), 122.7 (*C*H), 90.1 – 86.3 (m, 5 x *C*F), 65.1 (CH_2), 51.5 (*C*H), 50.5 (*C*H), 40.5 (CH_2), 38.6 (*C*H), 35.9-35.3 (m, *C*H), 33.4 (CH_2), 30.6 (CH_2), 29.0 (CH_2); ^{19}F NMR (376 MHz, Acetone-d_6) δ: -204.7 - -205.1 (m, C*F*), -211.7 - -212.0 (m, C*F*), -212.5 - -212.8 (m, C*F*), -217.2 - -217.5 (m, C*F*); HRMS (ASAP$^+$) $C_{25}H_{29}O_4N_3F_5$ $[M-H]^-$ found 530.2087, requires 530.2084.

N-((*S*)-1-Oxo-1-(((*S*)-1-oxo-3-((*S*)-2-oxopyrrolidin-3-yl)propan-2-yl)amino)-3-((*1r,2R,3R,4R,5S,6S*)-2,3,4,5,6-pentafluorocyclohexyl)propan-2-yl)cinnamamide 45

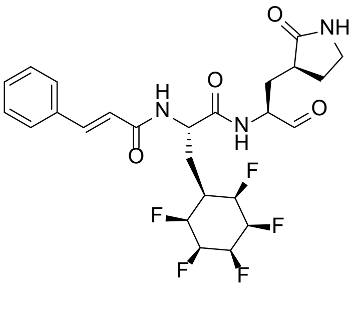

To a solution of N-((*S*)-1-(((*S*)-1-hydroxy-3-((*S*)-2-oxopyrrolidin-3-yl)propan-2-yl)amino)-1-oxo-3-((1*r*,2*R*,3*R*,4*R*,5*S*,6*S*)-2,3,4,5,6-pentafluorocyclohexyl)propan-2-yl)cinnamamide **44** (20 mg, 0.038 mmol, 1.0 equiv.) in Acetone:CH_2Cl_2 (1 ml:1 ml), added Dess-Martin periodinane (24 mg, 0.056 mmol, 1.5 equiv.) at 0 °C and then stirred at 20-25°C. Upon completion of the reaction (aprox. 6 hr), concentrated under vacuum, and the residue was purified by silica gel column chromatography (0-5 % MeOH in CH_2Cl_2) to afford pure N-((*S*)-1-oxo-1-(((*S*)-1-

oxo-3-((*S*)-2-oxopyrrolidin-3-yl)propan-2-yl)amino)-3-((1*r*,2*R*,3*R*,4*R*,5*S*,6*S*)-2,3,4,5,6-pentafluorocyclohexyl)propan-2-yl)cinnamamide **45** as a white solid (12 mg, 60%); m.p.: 225-227 °C; $[\alpha]_D^{20}$= -6.6 (*c* 0.7, MeOH); ^{1}H NMR (500 MHz, Acetone-*d*$_6$) δ: 9.53 (bs, 1 H,), 8.68 (d, *J* 6.4, 1 H (N*H*)), 7.64 – 7.50 (m, 3 H), 7.44 – 7.32 (m, 3 H), 6.90 (s, 1 H (N*H*)), 6.79 (d, *J* =15.7, 1 H), 5.50 – 4.74 (m, 7 H), 4.37–4.29 (m, 1H, N*H*), 3.37 – 3.21 (m, 2 H), 2.65 – 2.42 (m, 2 H), 2.40 – 2.10 (m, 3 H), 2.02 – 1.76 (m, 3 H); ^{13}C NMR (126 MHz, Acetonitrile-*d*$_3$) δ: 201.3 (H*C*=O), 180.4 (*C*=O), 175.5 (*C*=O), 172.9 (*C*=O), 166.9 (*C*=O), 141.3 (*C*H), 135.9 (*C*), 130.8 (*C*H), 129.9 (2x*C*H), 128.7 (2x*C*H), 122.0 (*C*H), 90.7-86.1 (m, 5x*C*HF), 58.9 (*C*H), 51.3 (*C*H), 41.0 (*C*H$_2$), 39.0 (*C*H), 36.2-35.4 (m, *C*H), 30.2 (*C*H$_2$), 30.1(*C*H$_2$), 29.1(*C*H$_2$); ^{19}F NMR (470 MHz, Acetone-*d*$_6$) δ: -217.7 – -217.2 (m, 2xC*F*), -213.0-212.8 (m, C*F*), -212– -211.6 (m, C*F*), -205.2 – -204.70 (m, 2xC*F*); HRMS (ASAP$^+$) $C_{25}H_{27}N_3O_4F_5$ $[M-H]^-$ found 528.1930, requires 528.1927; ν_{max}/cm^{-1} 1668 (C=O), 1653 (C=O), 1645 (C=O), 1136 and 1049 (C-F)

Methyl (*S*)-2-((*S*)-2-(1H-indole-2-carboxamido)-3-((*1r,2R,3R,4R,5S,6S*)-2,3,4,5,6-pentafluorocyclohexyl)propanamido)-3-((*S*)-2-oxopyrrolidin-3-yl)propanoate 46

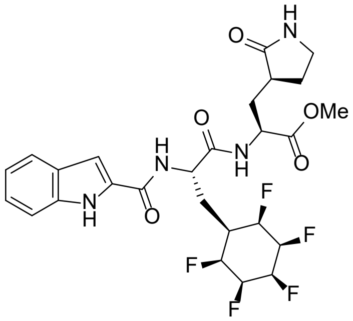

To a solution of methyl (*S*)-2-((*S*)-2-((tert-butoxycarbonyl)amino)-3-((1*r*,2*R*,3*R*,4*R*,5*S*,6*S*)-2,3,4,5,6-pentafluorocyclohexyl)propanamido)-3-((*S*)-2-oxopyrrolidin-3-yl)propanoate **40** (130 mg, 0.25 mmol, 1.0 equiv.) in MeOH (3 mL), added 4N.HCl in Dioxane (3.0 ml) and stirred at 20-25 °C. Upon completion of the reaction from TLC (aprox. 2 hr), evaporated the solvent and dried under vacuum. Residue dissolved in DMF (2.0 ml). To this added Indol-2-carboxylic acid (44 mg, 0.28 mmol, 1.1 equiv.), DIPEA (175 µL, 1.00 mmol, 4.0 equiv.) followed by HATU (105 mg, 0.28 mmol, 1.1 equiv.) at 20-25 °C and stirred for 16 hr. Upon completion of the reaction, Product was extracted in EtOAc (10 mL x 3), dried (anhydro.Na_2SO_4), filtered and evaporated under reduced pressure. The residue was purified by silica gel column chromatography (0-5% MeOH in DCM) to afford pure Methyl (*S*)-2-((*S*)-2-(1*H*-indole-2-carboxamido)-3-((1*r*,2*R*,3*R*,4*R*,5*S*,6*S*)-2,3,4,5,6-pentafluorocyclohexyl) propanamido)-3-((*S*)-2-oxopyrrolidin-3-yl)propanoate **46** as a white solid (115 mg, 82%); m.p.: 252-254 °C; $[\alpha]_D^{20}$ = -2.5 (*c* 0.4, MeOH); ^{1}H NMR (400 MHz, Methanol-*d*$_4$) δ: 7.61 (dt, *J* = 8.1, 1.0 Hz, 1H), 7.43 (dq, *J* = 8.3, 0.9 Hz, 1H), 7.22 (ddd, *J* = 8.3, 7.0, 1.1 Hz, 1H), 7.19 (d, *J* = 0.9 Hz, 1H), 7.06 (ddd, *J* = 8.0, 7.0, 1.0 Hz, 1H), 5.41 – 4.94 (m, 3H), 4.84 – 4.52 (m, 4H), 3.74 (s, 3H), 3.30 – 3.20 (m, 2H), 2.63 (qd, *J* = 10.4, 4.2 Hz, 1H), 2.44 (dt, *J* = 14.5, 7.3 Hz, 1H), 2.36 – 2.11 (m, 3H), 2.01 (d, *J* = 34.1 Hz, 1H), 1.93 – 1.70 (m, 2H); ^{13}C NMR (126 MHz, Methanol-*d*$_4$) δ: 181.8 (*C*=O), 174.2 (*C*=O), 173.9 (*C*=O), 164.1 (*C*=O), 138.5 (*C*), 131.5 (*C*), 128.9 (*C*), 125.3 (*C*H), 122.9 (*C*H), 121.3 (*C*H), 113.0 (*C*H), 105.4 (*C*H), 9.33 – 86.62 (m, 5 x *C*HF), 53.0 (*C*H), 52.2 (*C*H), 52.0 (*C*H), 41.5 (*C*H$_2$), 39.6 (*C*H), 36.77 – 36.16 (m, *C*H), 33.6 (*C*H$_2$), 29.7 (*C*H$_2$), 28.6 (*C*H$_2$); ^{19}F NMR (376 MHz, Methanol-*d*$_4$) δ: -205.96 (dt, *J* = 30.7, 10.7 Hz, C*F*), -212.9 - -213.4 (m, C*F*), -218.3 (td, *J* = 25.6, 25.1, 12.6 Hz, C*F*); HRMS (ESI$^+$) $C_{26}H_{29}N_4O_5F_5Na$ $[M+Na]^+$ found 595.1939, requires 595.1956; ν_{max}/cm^{-1} 1693 (C=O), 1685 (C=O), 1635 (C=O), 1136 and 1049 (C-F);

N-((*S*)-1-(((*S*)-1-Hydroxy-3-((*S*)-2-oxopyrrolidin-3-yl)propan-2-yl)amino)-1-oxo-3-((*1r,2R,3R,4R,5S,6S*)-2,3,4,5,6-pentafluorocyclohexyl)propan-2-yl)-1H-indole-2-carboxamide 47

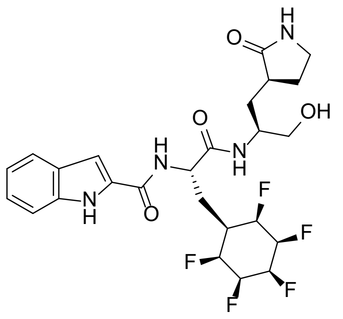

According to a literature procedure,[4] to a solution of methyl (*S*)-2-((*S*)-2-(1H-indole-2-carboxamido)-3-((1*r*,2*R*,3*R*,4*R*,5*S*,6*S*)-2,3,4,5,6-pentafluorocyclohexyl)propanamido)-3-((*S*)-2-oxopyrrolidin-3-yl)propanoate **46** (110 mg, 0.19 mmol, 1.0 equiv.) in anhydrous MeOH (5.0 mL) was added sodium borohydride (363 mg, 9.61 mmol, 50 equiv.) slowly at 0 °C. Then, the reaction mixture was stirred at r.t. for 16 h. Upon completion saturated NH_4Cl solution (20 mL) was added to quench the reaction. Evaporated methanol and extracted in EtOAc (25 mL × 3). The organic phase was washed with brine (5 mL), dried (anhyd.Na_2SO_4), filtered and concentrated under vacuum. The residue was purified by silica gel column chromatography (0-6 % MeOH in CH_2Cl_2) to afford N-((*S*)-1-(((*S*)-1-hydroxy-3-((*S*)-2-oxopyrrolidin-3-yl)propan-2-yl)amino)-1-oxo-3-((1*r*,2*R*,3*R*,4*R*,5*S*,6*S*)-2,3,4,5,6-pentafluorocyclohexyl)propan-2-yl)-1H-indole-2-carboxamide **47** as a white solid (93 mg, 89%); m.p.: 178-180 °C; $[\alpha]_D^{20}$ = -1.2 (*c* 1.0, MeOH); ^{1}H NMR (400 MHz, Methanol-*d*$_4$) δ: 7.60 (dt, *J* = 8.1, 1.0 Hz, 1H), 7.42 (dq, *J* = 8.4, 0.9 Hz, 1H), 7.22 (ddd, *J* = 8.3, 7.0, 1.1 Hz, 1H), 7.18 (d, *J* = 0.9 Hz, 1H), 7.06 (ddd, *J* = 8.0, 7.0, 1.0 Hz, 1H), 5.37 – 4.91 (m, 3H), 4.81 – 4.48 (m, 3H), 4.07 – 3.97 (m, 1H), 3.61 – 3.47 (m, 2H), 3.27 – 3.13 (m, 2H), 2.60 – 2.40 (m, 2H), 2.36 – 2.18 (m, 2H), 1.99 – 1.89 (m, 1H), 1.75 (dq, *J* = 12.6, 9.0 Hz, 1H), 1.49 (ddd, *J* = 14.3, 11.1, 3.4 Hz, 1H), 1.38 – 1.27 (m, 1H), 0.90 (t, *J* = 7.1 Hz, 1H); ^{13}C NMR (126 MHz, Methanol-*d*$_4$) δ: 182.7 (*C*=O), 174.0 (*C*=O), 164.1 (*C*=O), 138.4 (*C*), 131.5 (*C*), 128.9 (*C*), 125.3 (*C*H), 122.9 (*C*H), 121.3 (*C*H), 113.1 (*C*H), 105.4 (*C*H), 90.9 – 86.5 (m, 5 x *C*HF), 65.4 (*C*H$_2$), 52.6 (*C*H), 50.9 (*C*H), 41.5 (*C*H$_2$), 39.5 (*C*H), 36.7-36.3 (m, (*C*H)), 33.3 (*C*H$_2$), 29.8 (*C*H$_2$), 28.9 (*C*H$_2$); ^{19}F NMR (377 MHz, Methanol-*d*$_4$) δ: -205.96 (dt, *J* = 29.0, 11.1 Hz, C*F*), -212.60 (m, C*F*), -213.06 (m, C*F*), -218.17 (td, *J* = 25.8, 12.7 Hz, C*F*); HRMS (ASAP$^+$) $C_{25}H_{28}F_5N_4O_5$ $[M-H]^-$ found 543.2040, requires 543.2036; ν_{max}/cm^{-1} 1668 (C=O), 1653 (C=O), 1635 (C=O), 1134 and 1049 (C-F).

N-((*S*)-1-Oxo-1-(((*S*)-1-oxo-3-((*S*)-2-oxopyrrolidin-3-yl)propan-2-yl)amino)-3-((*1r,2R,3R,4R,5S,6S*)-2,3,4,5,6-pentafluorocyclohexyl)propan-2-yl)-1H-indole-2-carboxamide 48

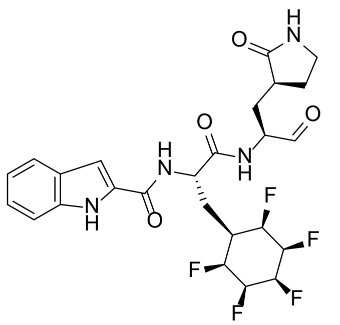

To a solution of *N*-((*S*)-1-(((*S*)-1-hydroxy-3-((*S*)-2-oxopyrrolidin-3-yl)propan-2-yl)amino)-1-oxo-3-((1*r*,2*R*,3*R*,4*R*,5*S*,6*S*)-2,3,4,5,6-pentafluorocyclohexyl)propan-2-yl)-1H-indole-2-carboxamide **47** (20 mg, 0.037 mmol, 1.0 equiv.) in Acetone:CH_2Cl_2 (1 ml:1 ml), added Dess-Martin periodinane (23 mg, 0.055 mmol, 1.5 equiv.) at 0 °C and then stirred at 20-25°C. Upon completion of the reaction (aprox. 6 hr), concentrated under vacuum, and the residue was purified by silica gel column chromatography (0-5 % MeOH in CH_2Cl_2) to afford pure N-((*S*)-1-oxo-1-(((*S*)-1-oxo-3-((*S*)-2-oxopyrrolidin-3-yl)propan-2-yl)amino)-3-((1*r*,2*R*,3*R*,4*R*,5*S*,6*S*)-2,3,4,5,6-pentafluorocyclohexyl)propan-2-yl)-1H-indole-2-carboxamide **48** as a white solid (14 mg, 70%); m.p.: 220-222 °C; $[\alpha]_D^{20}$ = -2.0 (*c* 1.0, MeOH); ^{1}H NMR (400 MHz, Acetone-*d*$_6$) δ: 10.86 (s, 1 H (N*H*)), 9.53 (d, *J* 0.7, 1 H, (N*H*)), 8.71

(1 H, d, *J* 6.6), 8.05 (d, *J* 8.3, 1 H (N*H*)), 7.68- – 7.57 (1 H, m), 7.54 (dd, *J* 8.2, 1.1, 1 H), 7.30 – 7.15 (m, 2 H), 7.07 (ddd, *J* 8.0, 7.0, 1.01 H), 7.00 (s, 1 H (N*H*)), 5.52 – 4.76 (m, 6 H), 3. 35 – 3. 18 (m, 2 H), 2.70 – 2.47 (m, 2 H), 2.37– 2.17 (m, 3 H), 2.03 – 1.94 (m, 1 H), 1.89 – 1.74 (m, 2 H), 1.59 (m, 1 H); ^{19}F NMR (376 MHz, Acetone-d_6) δ: -204.7– -205.2 (m, 2x C*F*), -211.4 – -212.0 (m, C*F*), -212.5– -213.0 (m, C*F*), -217.2– -217.6 (m, C*F*); ^{13}C NMR (126 MHz, Acetone-d_6) δ: 200.9 (*C*HO), 180.0 (*C*=O), 172.8 (*C*=O), 162.6 (*C*=O), 137.8 (*C*), 131.9 (*C*), 128.6 (*C*), 124.9 (*C*H), 122.6 (*C*H), 121.0 (*C*H), 113.1 (*C*H), 104.4 (*C*H), 90.5 – 86.2 (m, 5 x *C*HF), 58.8 (*C*H), 51.3 (*C*H), 40.8 (*C*H$_2$), 39.0 (*C*H), 36.2-35.8 (m, *C*H), 30.6 (*C*H), 30.5 (*C*H$_2$), 30.4 (*C*H$_2$), 29.2 (*C*H$_2$); HRMS (ESI$^-$) $C_{25}H_{26}N_4O_4F_5$ [M-H]$^-$ found 541.1882, requires 541.1880; ν_{max}/cm^{-1} 1668 (C=O), 1653 (C=O), 1647 (C=O), 1136 and 1051 (C-F).

Ethyl (*S,E*)-4-((*S*)-2-((*tert*-butoxycarbonyl)amino)-3-((*1r,2R,3R,4R,5S,6S*)-2,3,4,5,6-pentafluorocyclohexyl)propanamido)-5-((*S*)-2-oxopyrrolidin-3-yl)pent-2-enoate 50

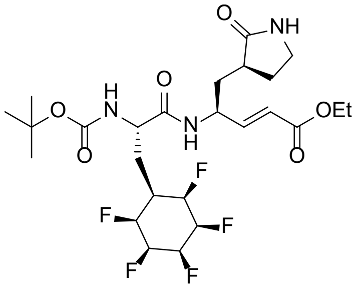

To a solution of ethyl (*S,E*)-4-((tert-butoxycarbonyl)amino)-5-((*S*)-2-oxopyrrolidin-3-yl)pent-2-enoate **49** (140 mg, 0.43 mmol, 1.0 equiv.) in CH_2Cl_2 (5 ml), added TFA (2.45 mL, 21.50 mmol, 50 equiv.) and stirred at 20-25 °C. Upon completion of the reaction from TLC (aprox. 2-3 hr), evaporated the solvent and dried under vacuum for 1.0 hr. Residue dissolved in DMF (2.0 ml). To this, added (2*S*)-2-((*tert*-butoxycarbonyl)amino)-3-(2,3,4,5,6-pentafluorocyclohexyl)propanoic acid **38** (140 mg, 0.39 mmol, 0.9 equiv.), DIPEA (300 µL, 1.72 mmol, 4.0 equiv.) followed by HATU (164 mg, 0.43 mmol, 1.0 equiv.) at 20-25 °C and stirred for 16 hr. Upon completion of the reaction, product was extracted with EtOAc (10 mL x 3), dried (anhyd.Na_2SO_4), filtered and evaporated under reduced pressure. The residue was purified by silica gel column chromatography (0-5% MeOH in DCM) to afford pure ethyl (*S,E*)-4-((*S*)-2-cinnamamido-3-((1*r*,2*R*,3*R*,4*R*,5*S*,6*S*)-2,3,4,5,6-pentafluorocyclohexyl) propanamido)-5-((*S*)-2-oxopyrrolidin-3-yl)pent-2-enoate **50** as a white solid (139 mg, 57%); m.p.: 186-188 °C; $[\alpha]_D^{20}$ = -25.8 (*c* 1.0, MeOH); ^{1}H NMR (400 MHz, Acetone-d_6) δ: 7.99 (d, *J* = 8.1 Hz, 0H), 6.92 (dd, *J* = 15.6, 5.1 Hz, 1H), 6.35 (d, *J* = 8.3 Hz, 0H), 5.95 (dt, *J* = 15.7, 1.6 Hz, 0H), 5.40 (d, *J* = 53.9 Hz, 0H), 5.26 – 4.59 (m, 2H), 4.39 – 4.21 (m, 0H), 4.13 (qd, *J* = 7.1, 0.9 Hz, 1H), 3.38 – 3.19 (m, 1H), 2.54 – 2.27 (m, 1H), 2.25 – 2.10 (m, 0H), 2.05 – 1.92 (m, 1H), 1.88 – 1.74 (m, 1H), 1.65 (ddd, *J* = 13.7, 9.4, 4.0 Hz, 0H), 1.40 (s, 5H), 1.23 (t, *J* = 7.1 Hz, 2H); ^{13}C NMR (126 MHz, Acetone-d_6) δ: 179.9 (*C*=O), 172.6 (*C*=O), 166.5 (*C*=O), 156.7 (*C*=O), 149.3 (*C*H), 121.3 (*C*H), 90.5-86.4 (5 x *C*F), 79.8 (*C*(CH$_3$)$_3$), 60.7 (*C*H$_2$), 53.2 (*C*H), 49.6 (*C*H), 40.7 (*C*H$_2$), 38.8 (*C*H), 36.1 (*C*H$_2$), 28.9 (*C*H$_2$), 28.5 (*C*H$_3$), 14.5 (*C*H); ^{19}F NMR (376 MHz, Acetone-d_6) δ -203.9-204.2 (m, C*F*), -211.0-211.2 (m, C*F*), -211.6-211.8 (m,. C*F*), -216.5-216.8 (m, C*F*); HRMS (ESI$^+$) $C_{29}H_{33}N_3O_5F_5$ [M+Na]$^+$ found 592.2408, requires 592.2416; ν_{max}/cm^{-1} 1683 (C=O), 1670 (C=O), 1134 and 1047 (C-F).

Ethyl (*S,E*)-4-((*S*)-2-cinnamamido-3-((*1r,2R,3R,4R,5S,6S*)-2,3,4,5,6-pentafluorocyclohexyl)propanamido)-5-((*S*)-2-oxopyrrolidin-3-yl)pent-2-enoate 52

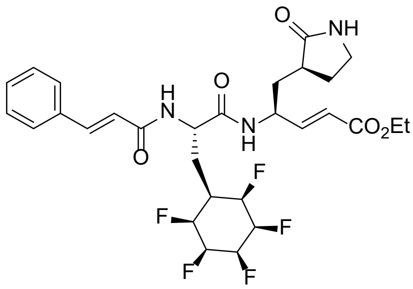

To a solution of ethyl (*S*,*E*)-4-((*S*)-2-((tert-butoxycarbonyl)amino)-3-((1*R*,2*R*,3*R*,4*R*,5*S*,6*S*)-2,3,4,5,6-pentafluorocyclohexyl)propanamido)-5-((*S*)-2-oxopyrrolidin-3-yl)pent-2-enoate **49** (120 mg, 0.21 mmol, 1.0 equiv.) in MeOH (5 ml), added 4N HCl in Dioxane (5.0 ml) and stirred at 20-25 °C. Upon completion of the reaction (aprox. 2 hr) from TLC, evaporated the solvent and dried under vacuum for 1.0 hr (Due to the reaction in MeOH, 50 % of methyl trans-esterified product on the conjugate ester was formed). Residue dissolved in DMF (1.5 ml). To this, added *E*-cinnamic acid (40 mg, 0.27 mmol, 1.3 equiv.), DIPEA (146 µL, 0.84 mmol, 4.0 equiv.) followed by HATU (80 mg, 0.21 mmol, 1.0 equiv.) at 20-25 °C and stirred for 16 hr. Upon completion of the reaction, Product was extracted with EtOAc (10 mL x 3), dried (anhyd.Na_2SO_4), filtered and evaporated under reduced pressure. The residue was purified by silica gel column chromatography (0-5% MeOH in DCM) to afford pure ethyl (*S*,*E*)-4-((*S*)-2-cinnamamido-3-((1*r*,2*R*,3*R*,4*R*,5*S*,6*S*)-2,3,4,5,6-pentafluorocyclohexyl) propanamido)-5-((*S*)-2-oxopyrrolidin-3-yl)pent-2-enoate **52** as a white solid (96 mg, 76%, 1:1 mixture of Et and Me ester); m.p.: 248-250 °C; $[\alpha]_D^{20}$ = -8.2 (*c* 0.6, MeOH); ^{1}H NMR (400 MHz, DMSO-d_6) δ: 8.48 (dd, *J* = 8.5, 5.0 Hz, 1H), 8.42 (d, *J* = 8.0 Hz, 1H), 7.64 – 7.34 (m, 6H), 6.88 (dt, *J* = 15.8, 5.5 Hz, 1H), 6.74 (d, *J* = 15.8 Hz, 1H), 5.86 (ddd, *J* = 15.7, 8.2, 1.6 Hz, 1H), 5.36 (m, 1H), 5.20 – 4.71 (m, 4H), 4.64-4.51 (m, 2H), 4.12 (td, *J* = 7.4, 6.2 Hz, 2H of 50% of ethyl ester OCH_2)), 3.66 (s, 3H of 50% methyl ester OCH_3), 3.18-3.03 (m, 2H), 2.35 – 2.24 (m, 1H), 2.22 – 1.83 (m, 5H), 1.71 – 1.59 (m, 1H), 1.55 – 1.44 (m, 1H), 1.20 (t, *J* = 7.1 Hz, 3H of 50% Ethyl ester CH_3); ^{13}C NMR (126 MHz, DMSO-d_6) δ: 178.4 (*C*=O), 171.1 (*C*=O), 166.1 (*C*=O of *C*$OOCH_3$), 165.7 (*C*=O of *C*$OOCH_2CH_3$), 165.4 (*C*=O), 149.4 (*C*H=CH of Et/Me ester), 149.2 (*C*H=CH of Et/Me ester), 139.3 (*C*H), 134.8 (*C*), 129.7 (*C*H), 129.1 (2 x *C*H), 127.6 (2 x *C*H), 121.9 (*C*H), 119.9 (*C*H=CH of Et/Me ester), 119.6 (*C*H=CH of Et/Me ester), 89.65 – 85.15 (m, 5 x *C*HF), 60.1 (*C*H_2), 51.6 (*C*H), 50.2 (*C*H), 47.8 (*C*H), 37.7 (*C*H), 34.8 (bs, 2 x *C*H_2), 34.65-34.21 (m, *C*H), 29.0 (*C*H_2), 27.4 (*C*H_2), 14.2 (*C*H_3 of Et ester); ^{19}F NMR (376 MHz, DMSO-d_6) δ: -204.6 (dt, *J* = 55.0, 11.6 Hz, 2 x C*F*), -211.6 (m, C*F*), -212.3 (m, C*F*), -217.0 (m, C*F*); HRMS (ESI$^-$) $C_{29}H_{33}N_3O_5F_5$ [M-H]$^-$ found 598.2345, requires 598.2346; ν_{max}/cm^{-1} 1683 (C=O), 1647 (C=O); 1128 and 1051 (C-F).

Ethyl (*S*,*E*)-4-((*S*)-2-(1H-indole-2-carboxamido)-3-((*1r,2R,3R,4R,5S,6S*)-2,3,4,5,6-pentafluorocyclohexyl)propanamido)-5-((*S*)-2-oxopyrrolidin-3-yl)pent-2-enoate 53

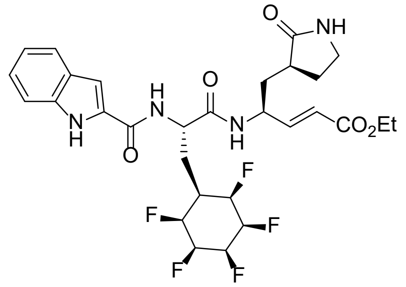

To a solution of ethyl (*S*,*E*)-4-((*S*)-2-((tert-butoxycarbonyl)amino)-3-((1*R*,2*R*,3*R*,4*R*,5*S*,6*S*)-2,3,4,5,6-pentafluorocyclohexyl)propanamido)-5-((*S*)-2-oxopyrrolidin-3-yl)pent-2-enoate **49** (86 mg, 0.15 mmol, 1.1 equiv.) in EtOH (3 ml), added 4N HCl in Dioxane (3.0 ml) and stirred at 20-25 °C. Upon completion of the reaction from TLC (aprox. 5 hr), evaporated the solvent and dried under vacuum for 1.0 hr. Residue dissolved in DMF (1.0 ml). To this, added indol-2-carboxylic acid (24 mg, 0.14 mmol, 1.0 equiv.), DIPEA (105 µL, 0.6 mmol, 4.0 equiv.) followed by HATU (57 mg, 0.14 mmol, 1.0 equiv.) at 20-25 °C and stirred for 16 hr. Upon completion of the reaction, Product was extracted with EtOAc (10 mL x 3), dried (anhyd.Na_2SO_4), filtered and evaporated under reduced

pressure. The residue was purified by silica gel column chromatography (0-5% MeOH in CH_2Cl_2) to afford pure ethyl (*S*,*E*)-4-((*S*)-2-cinnamamido-3-((1*r*,2*R*,3*R*,4*R*,5*S*,6*S*)-2,3,4,5,6-pentafluorocyclohexyl) propanamido)-5-((*S*)-2-oxopyrrolidin-3-yl)pent-2-enoate **53** as white solid (72 mg, 78%); m.p.: 226-228 °C; $[\alpha]_D^{20}$ = -7.5 (*c* 1.0, MeOH); ^{1}H NMR (400 MHz, Acetone-d_6) δ: 10.94 (s, 1H), 8.28 (d, *J* = 8.0 Hz, 1H), 8.16 (d, *J* = 8.4 Hz, 1H), 7.64 – 7.58 (m, 1H), 7.54 (dt, *J* = 8.3, 0.9 Hz, 1H), 7.23 (ddd, *J* = 8.3, 7.0, 1.2 Hz, 1H), 7.19 (dd, *J* = 2.2, 0.9 Hz, 1H), 7.11 – 7.02 (m, 2H), 6.92 (dd, *J* = 15.7, 5.5 Hz, 1H), 5.97 (dd, *J* = 15.7, 1.6 Hz, 1H), 5.49 – 4.69 (m, 7H), 4.12 (q, *J* = 7.1 Hz, 2H), 3.31 – 3.12 (m, 2H), 2.70-2.58 (m, 1H), 2.58 – 2.47 (m, 1H), 2.42 – 2.15 (m, 3H), 2.04 – 1.98 (m, 1H), 1.84-1.71 (dq, *J* = 12.3, 9.1 Hz, 1H), 1.70-1.60 (ddd, *J* = 13.7, 8.9, 4.4 Hz, 1H), 1.22 (t, *J* = 7.1 Hz, 3H); ^{13}C NMR (126 MHz, Acetone-d_6) δ: 180.3 (*C*=O), 172.0 (*C*=O), 166.5 (*C*=O), 162.8 (*C*=O), 149.1 (*C*H), 137.8 (*C*), 131.8 (*C*), 128.5 (*C*), 124.9 (*C*H), 122.6 (*C*H), 121.5 (*C*H), 121.0 (*C*H), 113.1 (*C*H), 104.4 (*C*H), 90.8 – 86.3 (m, 5 x *C*F), 60.8 (CH_2), 51.6 (*C*H), 49.8 (*C*H), 40.8 (CH_2), 39.0 (*C*H), 36.2 (m, *C*H), 36.0 (CH_2), 29.0 (CH_2), 14.5 (CH_3); ^{19}F NMR (377 MHz, Acetone-d_6) δ: -205.0 (dt, *J* = 31.4, 11.3 Hz, 2 x CF), -211.32 (m, C*F*), -212.80 (m, C*F*), -217.34 (m, C*F*); HRMS (ESI$^-$) $C_{29}H_{32}N_4O_5F_5$ [M-H]$^-$ found 611.2299, requires 611.2298; ν_{max}/cm^{-1} 1683 (C=O), 1647 (C=O), 1635 (C=O), 1136 and 1049 (C-F).

Notes & References

[1] C.-J. Kuo, J.-J. Shie, J.-M. Fang, G.-R. Yen, J. T.-A. Hsu, H.-G. Liu, S.-N. Tseng, S.-C. Chang, C.-Y. Lee, S.-R. Shih, P.-H. Liang, *Bioorg. Med. Chem.,* **2008,** *16*, 7388–7398

[2] Y. Wei, B. Rao, X. Cong, X. Zeng, *J. Am. Chem. Soc.,* **2015**, *137*, 9250–9253.

[3] P. P. Geurink, N. Liu, M. P. Spaans, S. L. Downey, A. M. C. H. van den Nieuwendijk, G. A. van der Marel, A. F. Kisselev, B. I. Florea, H. S. Overkleeft, *J. Med. Chem.,* **2010**, *53*, 2319–2323

[4] Methyl (*S*)-2-((*tert*-butoxycarbonyl)amino)-3-(perfluorophenyl)propanoate **36** was prepared as described in reference 3 above, from the commercially available amino acid N-Boc-L-(pentafluoroaryl)phenylalanine, purchased from Fluorochem. Ltd., Catalogue No 008086; CAS Number 34702-60-8.

[5] Y. Zhai, X. Zhao, Z. Cui, M. Wang, Y. Wang, L. Li, Q. Sun, D. Zeng, Y. Liu, Y. Sun, Z. Lou, L. Shang, Z. Yin, *J. Med. Chem.* **2015**, *58*, 9414−9420.

NMR Spectra

methyl 2-((1r,2R,3R,4s,5S,6S)-2,3,4,5,6-pentafluorocyclohexyl)acetate 16

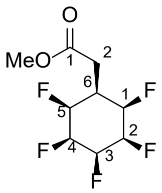

^{1}H NMR (700 MHz, Acetone-d_6)

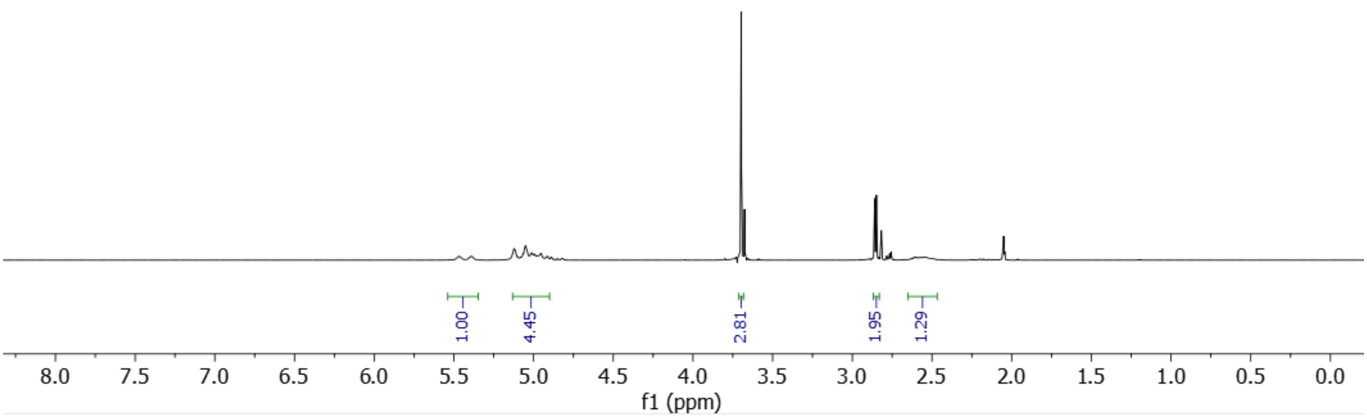

^{19}F NMR (659 MHz, Acetone-d_6)

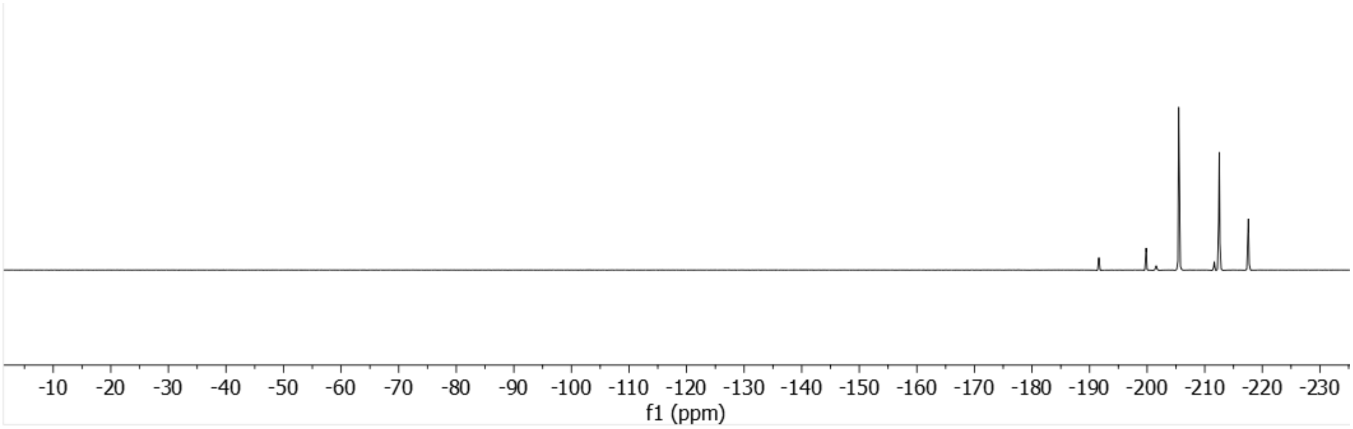

^{13}C NMR (126 MHz, Acetone-d_6)

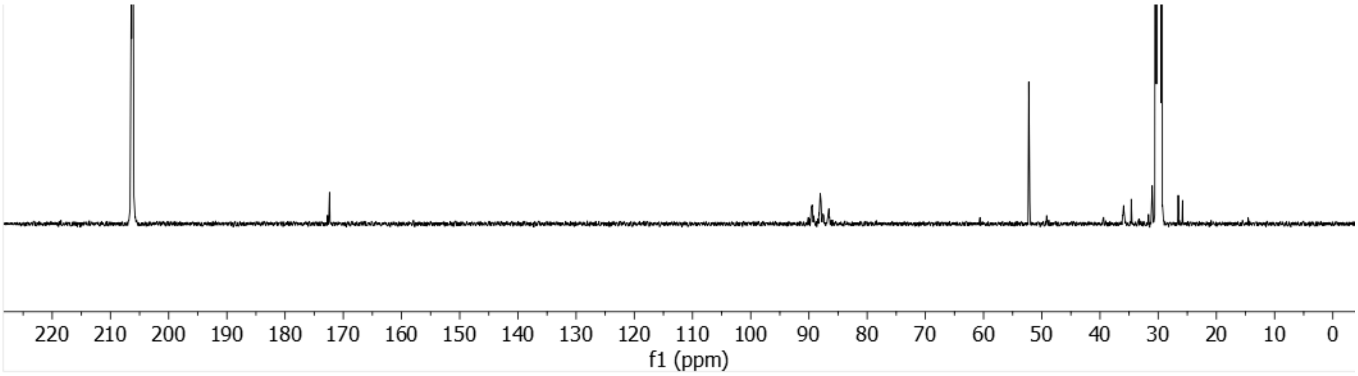

methyl 2-((1S,2S,3R,4R,6S)-2,3,4,6-tetrafluorocyclohexyl)acetate 18

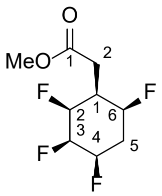

^{1}H NMR (500 MHz, Acetone-d_6)

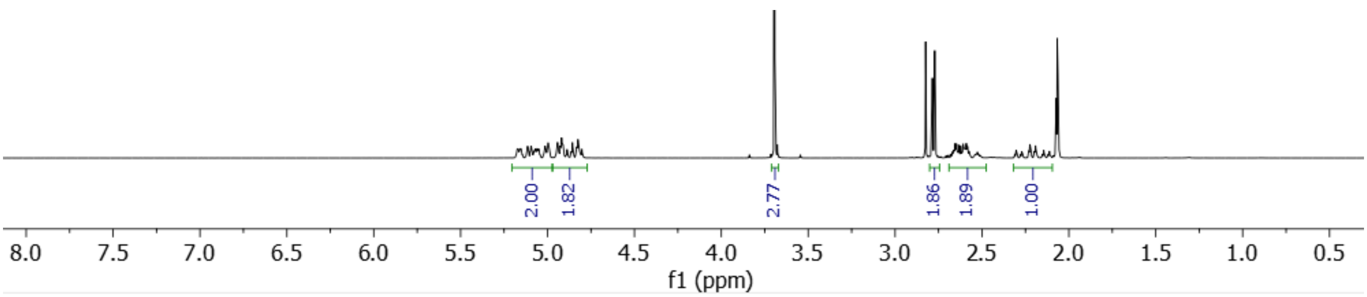

^{19}F NMR (470 MHz, Acetone-d_6)

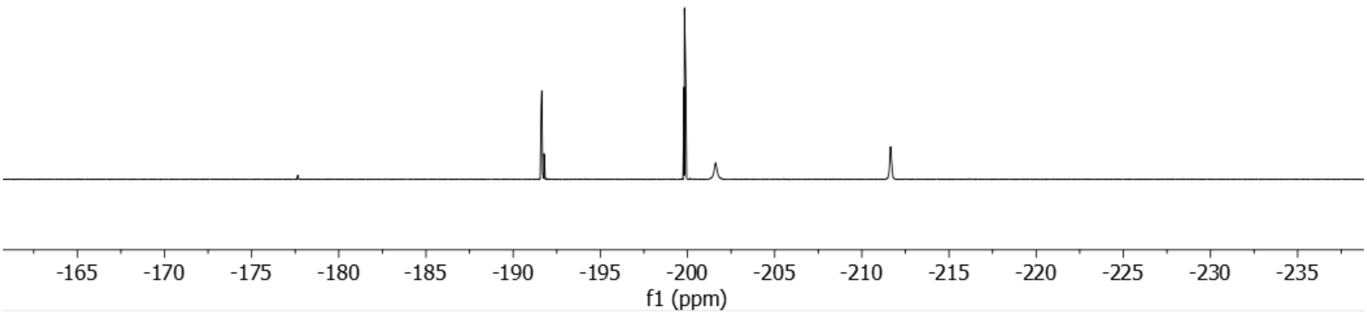

^{13}C NMR (126 MHz, Acetone-d_6)

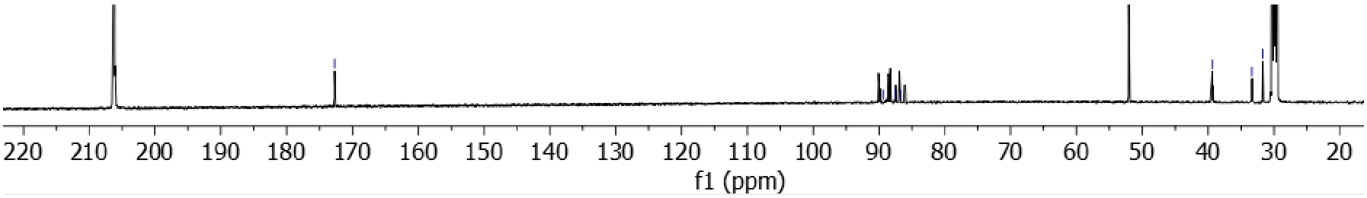

2-((1r,2R,3R,4s,5S,6S)-2,3,4,5,6-pentafluorocyclohexyl)ethan-1-ol 19

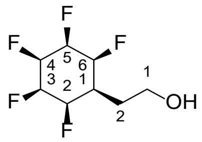

^{1}H NMR (500 MHz, Methanol-*d*$_4$)

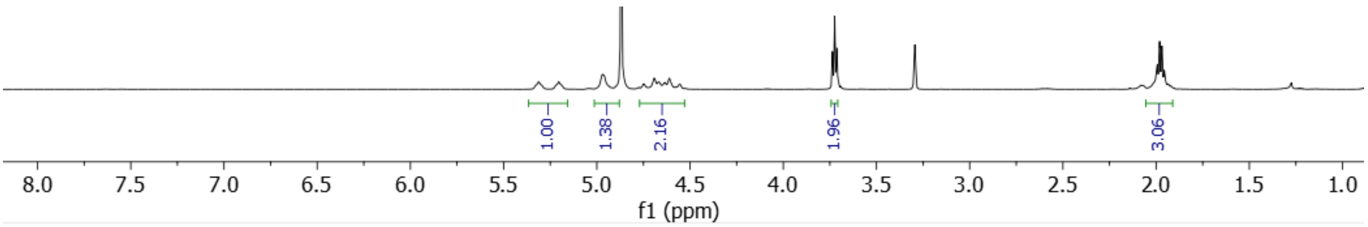

^{19}F NMR (659 MHz, Methanol-*d*$_4$)

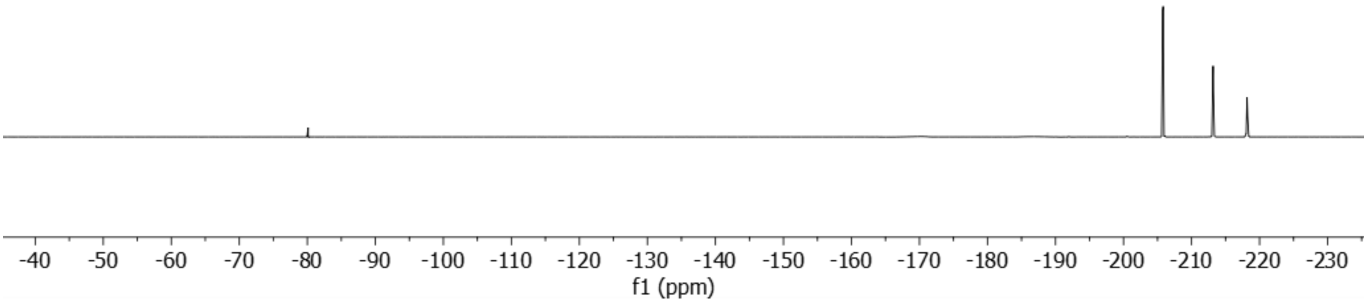

^{13}C NMR (126 MHz, Methanol-*d*$_4$)

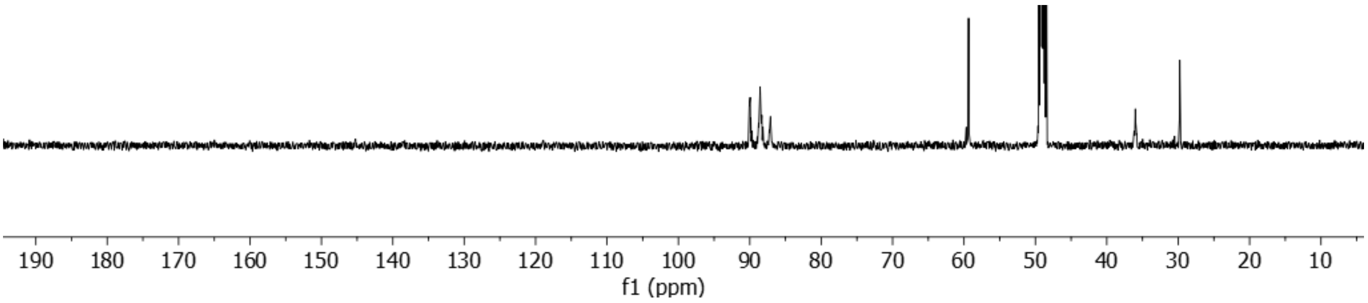

2-((1r,2R,3R,4s,5S,6S)-2,3,4,5,6-pentafluorocyclohexyl)acetaldehyde 13

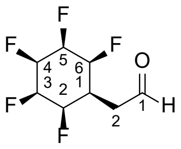

^{1}H NMR (500 MHz, Acetone-d_6)

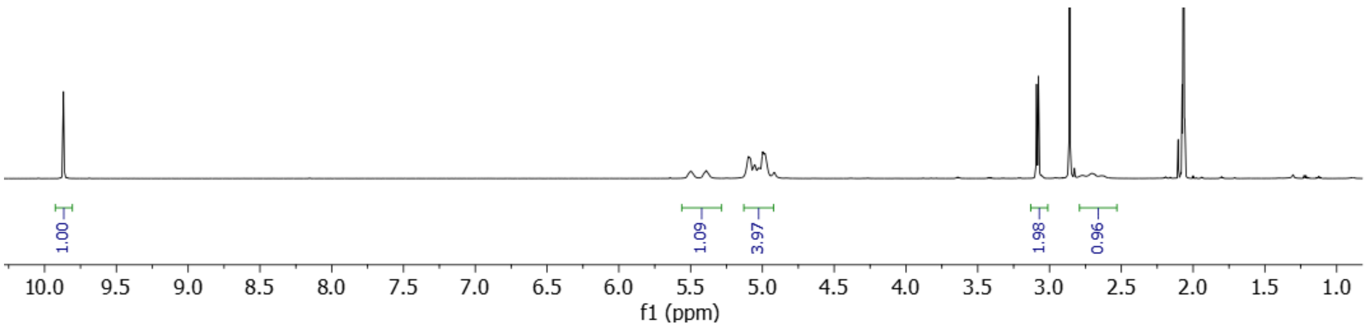

^{19}F NMR (471 MHz, Acetone-d_6)

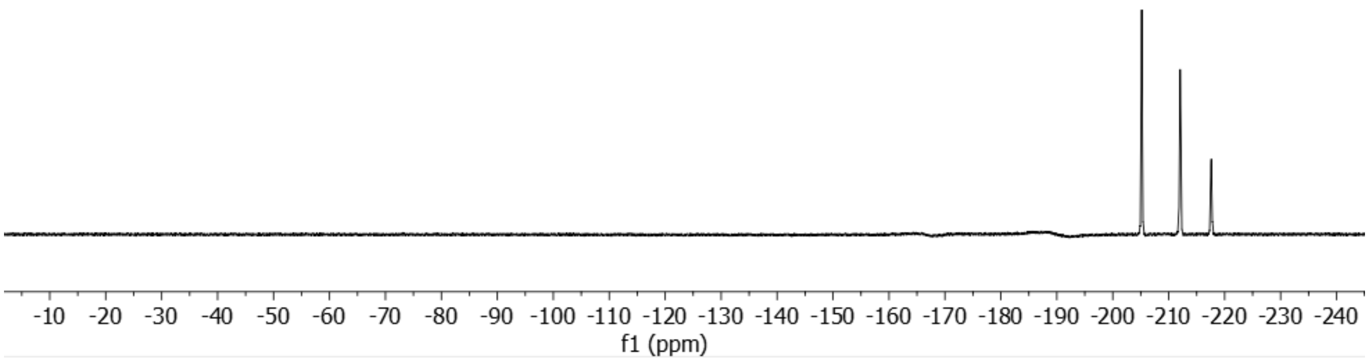

^{13}C NMR (126 MHz, Acetone-d_6)

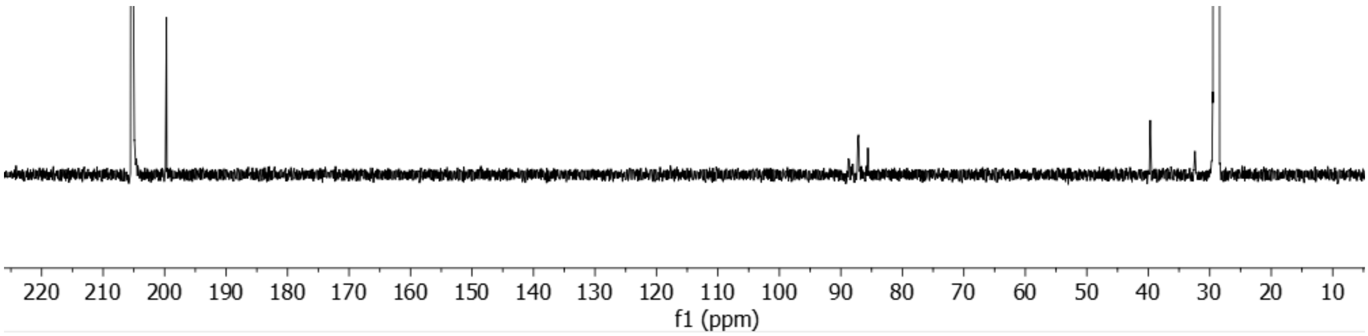

2-(N-allylacetamido)-N-benzyl-3-((1r,2R,3R,4s,5S,6S)-2,3,4,5,6-pentafluorocyclohexyl)propenamide 20

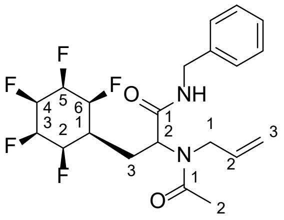

^{1}H NMR (500 MHz, $CDCl_3$)

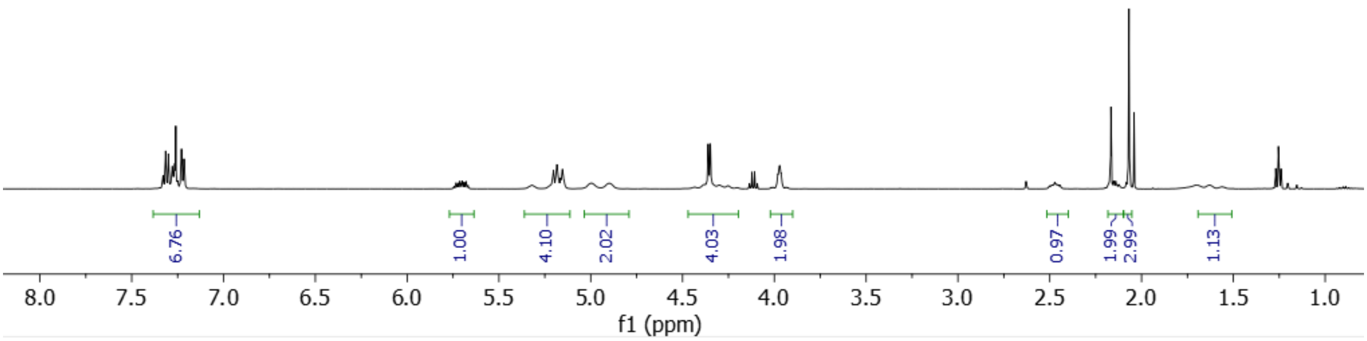

^{19}F NMR (470 MHz, $CDCl_3$)

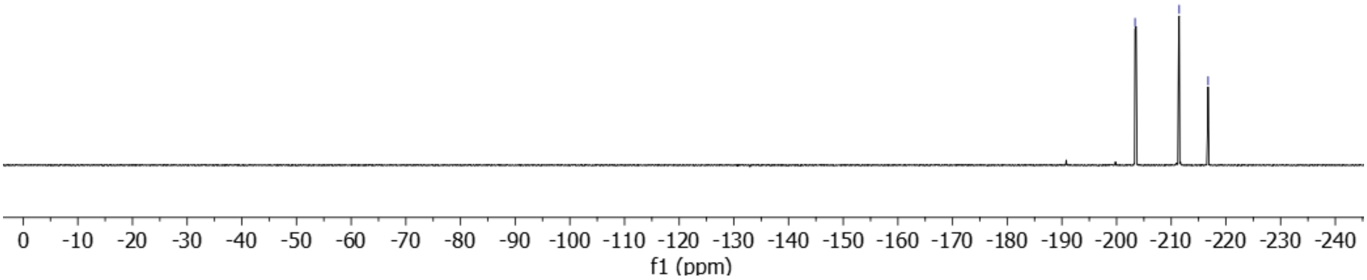

^{13}C NMR (126 MHz, $CDCl_3$)

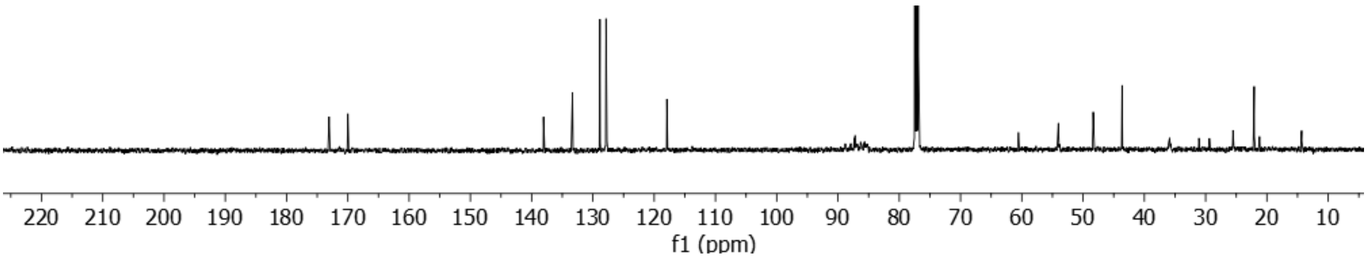

2-(N-allyl-2-(2-chlorophenyl)acetamido)-N-benzyl-3-((1r,2R,3R,4s,5S,6S)-2,3,4,5,6-pentafluorocyclohexyl)propenamide 21

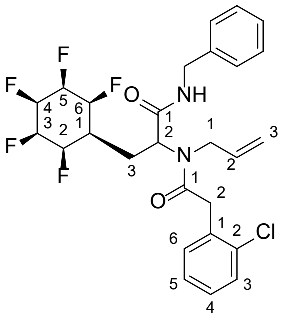

^{1}H NMR (500 MHz, $CDCl_3$)

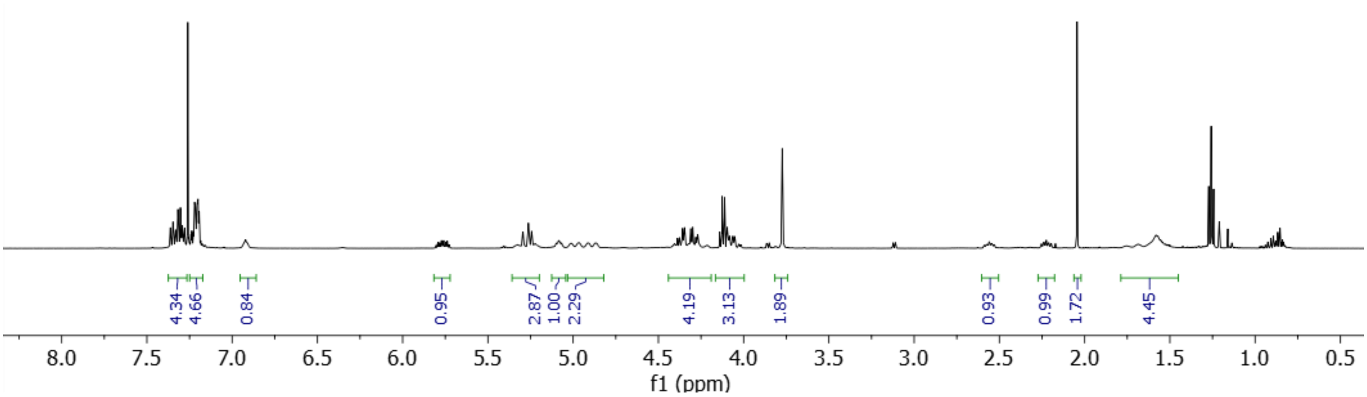

^{19}F NMR (471 MHz, $CDCl_3$)

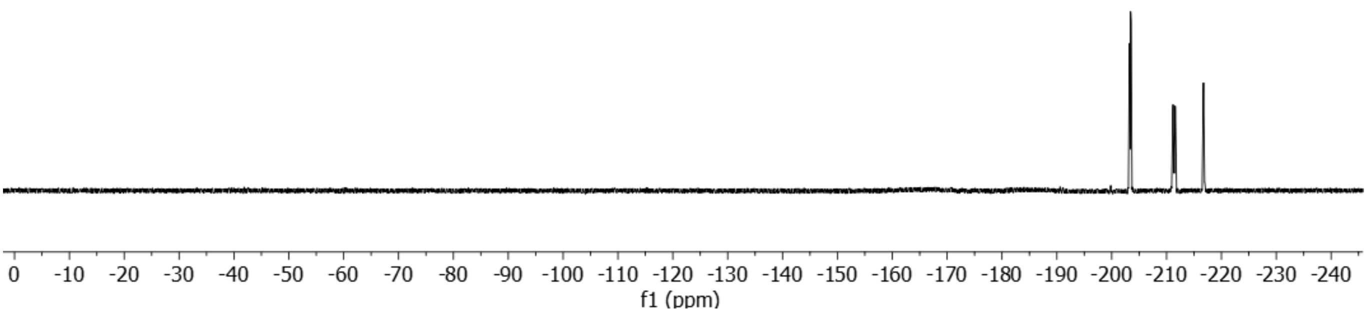

^{13}C NMR (126 MHz, $CDCl_3$)

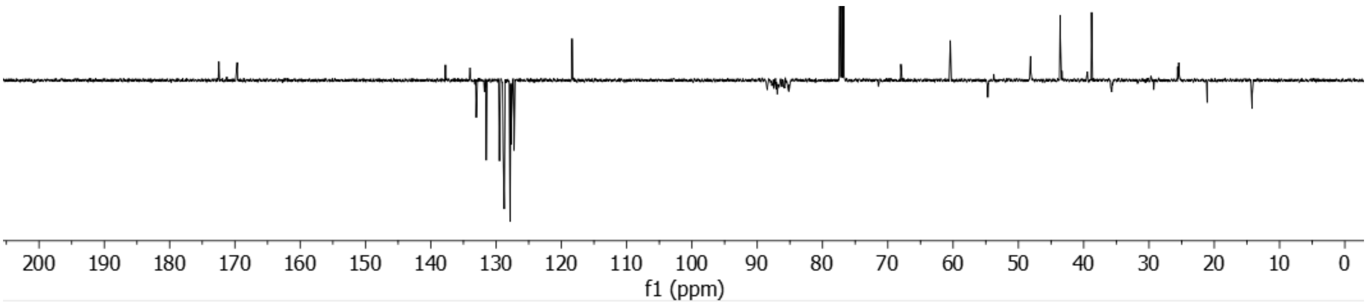

N-benzyl-2-(N-benzylacetamido)-3-((1r,2R,3R,4s,5S,6S)-2,3,4,5,6-pentafluorocyclohexyl)propenamide 22

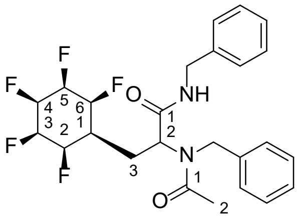

^1H NMR (500 MHz, $CDCl_3$)

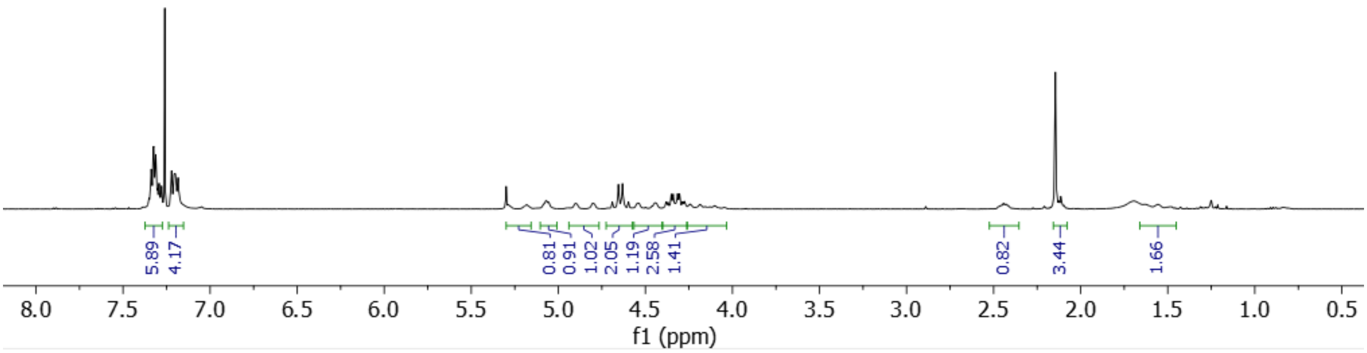

^{19}F NMR (470 MHz, $CDCl_3$)

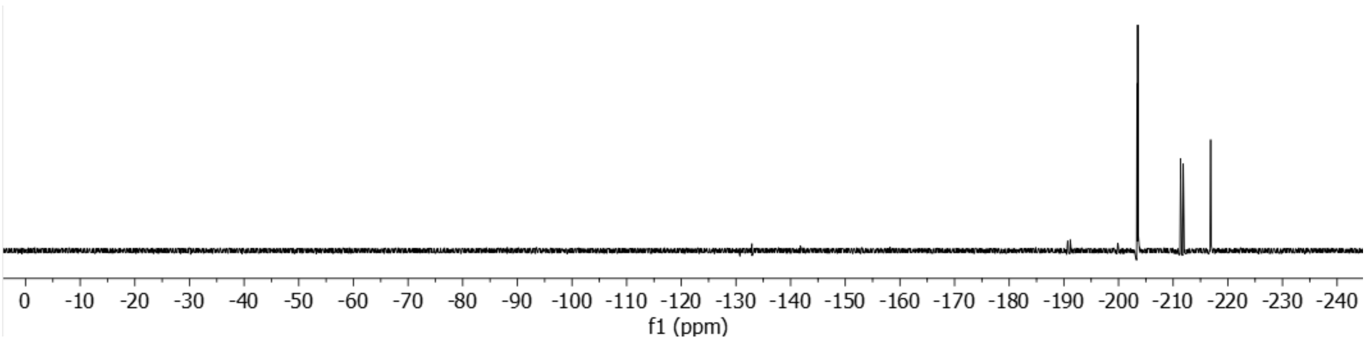

^{13}C NMR (126 MHz, $CDCl_3$)

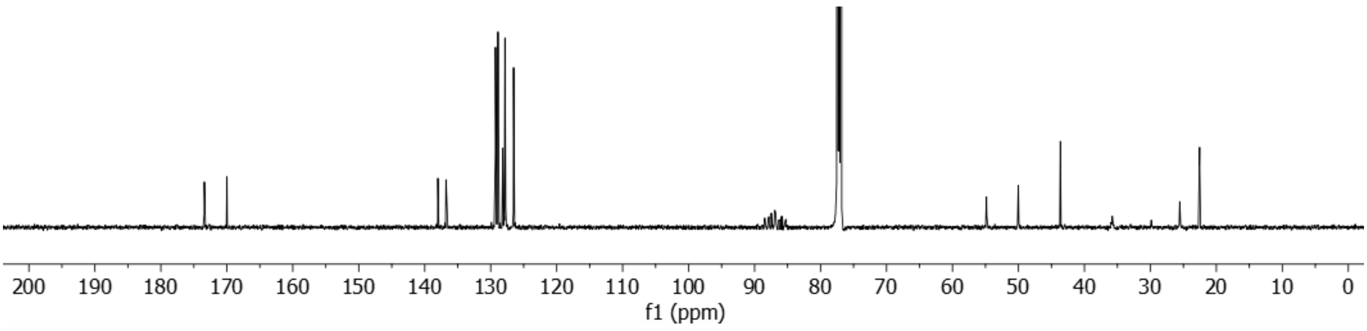

***tert*-butyl ((2S)-1-(benzyl(1-(benzylamino)-1-oxo-3-((1r,2R,3R,4R,5S,6S)-2,3,4,5,6-pentafluorocyclohexyl)propan-2-yl)amino)-3-methyl-1-oxobutan-2-yl)carbamate 23**

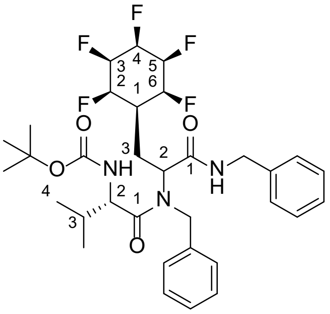

^{1}H NMR (500 MHz, $CDCl_3$)

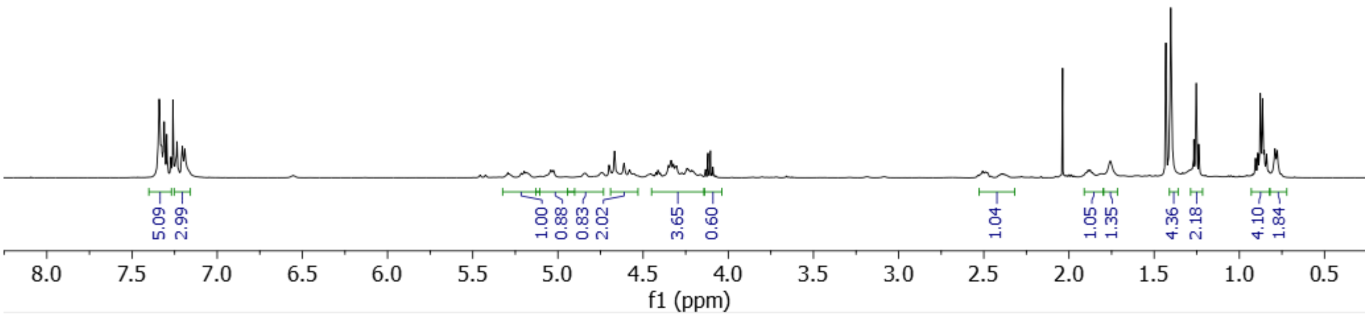

^{19}F NMR (471 MHz, $CDCl_3$)

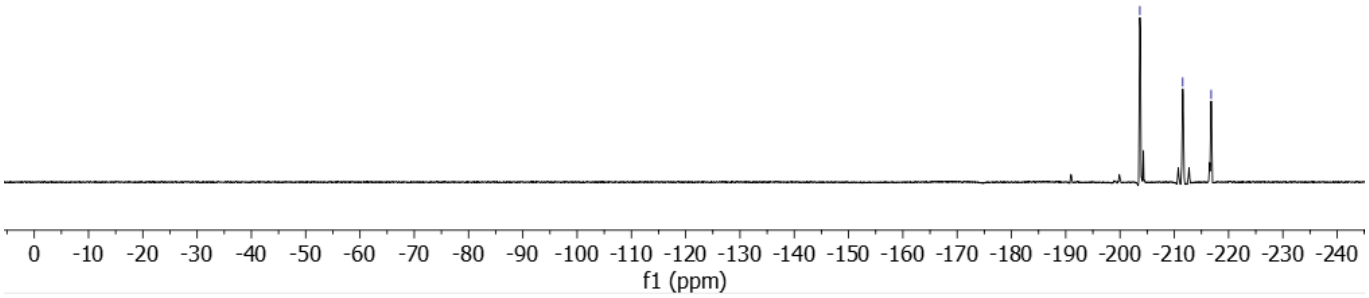

^{13}C NMR (126 MHz, $CDCl_3$)

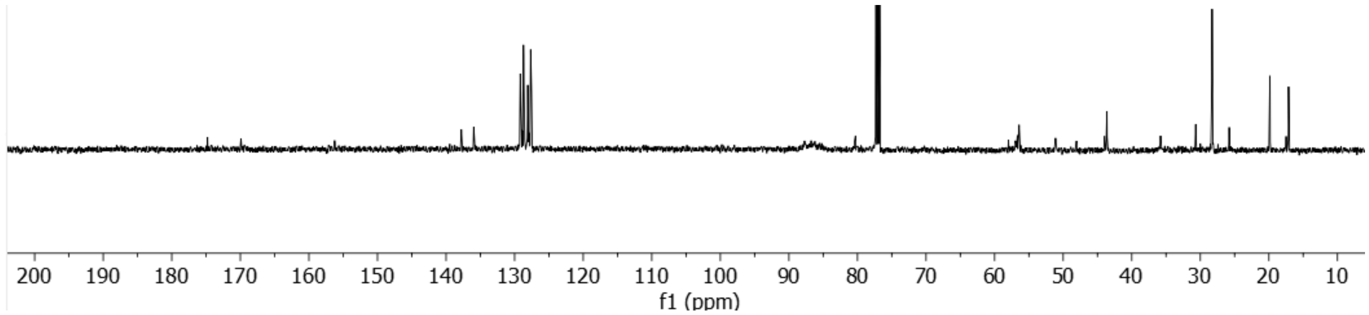

2-(N-allyl-2,2,2-trifluoroacetamido)-N-benzyl-3-((1r,2R,3R,4s,5S,6S)-2,3,4,5,6-pentafluorocyclohexyl)propenamide 24

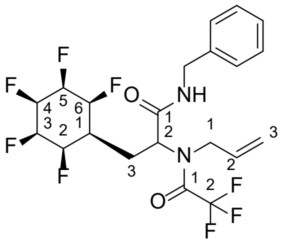

^{1}H NMR (400 MHz, Methanol-d_4)

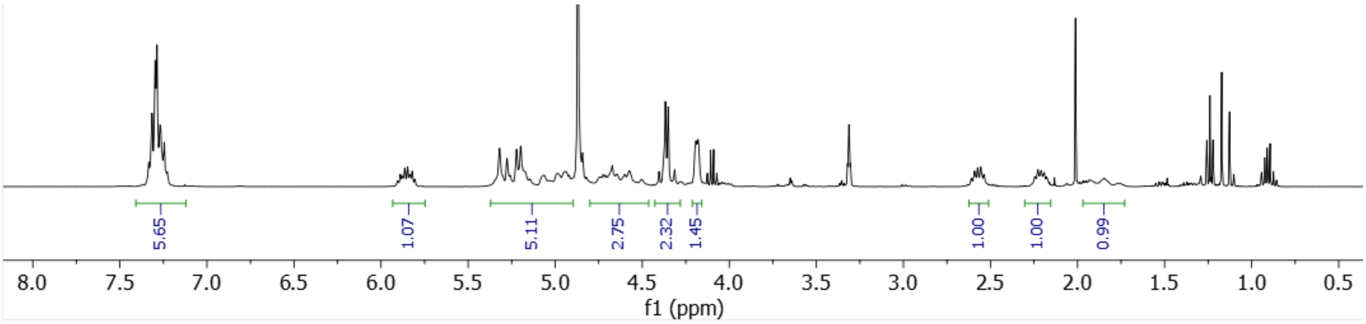

^{19}F NMR (377 MHz, Methanol-d_4)

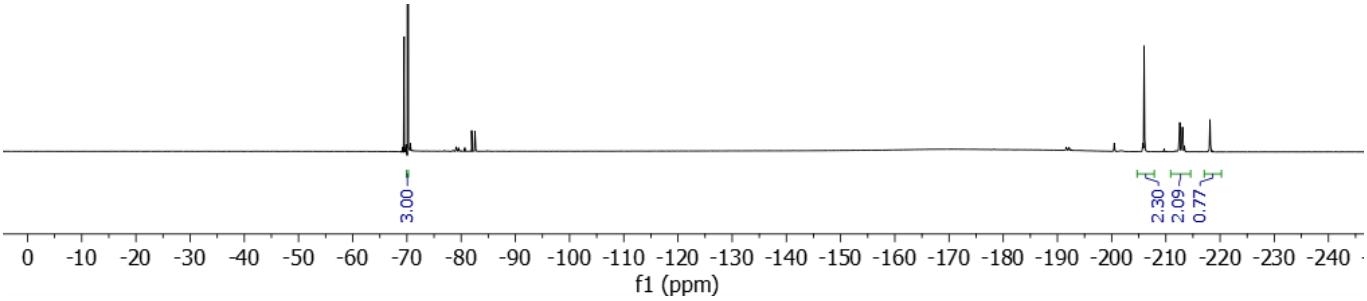

^{13}C NMR (126 MHz, Methanol-d_4)

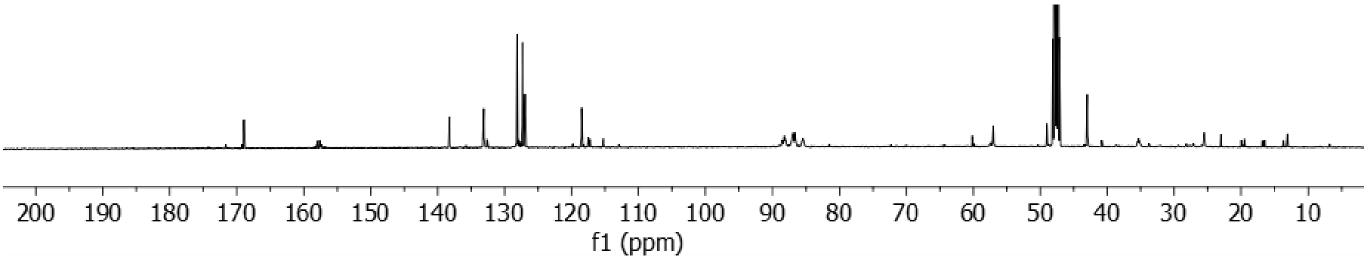

N-benzyl-N-(1-(benzylamino)-1-oxo-3-((1r,2R,3R,4s,5S,6S)-2,3,4,5,6-pentafluorocyclohexyl)propan-2-yl)propiolamide 25

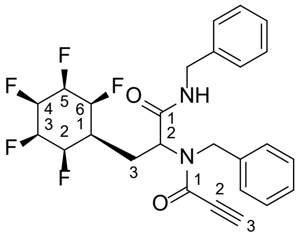

^{1}H NMR (500 MHz, Acetone-d_6)

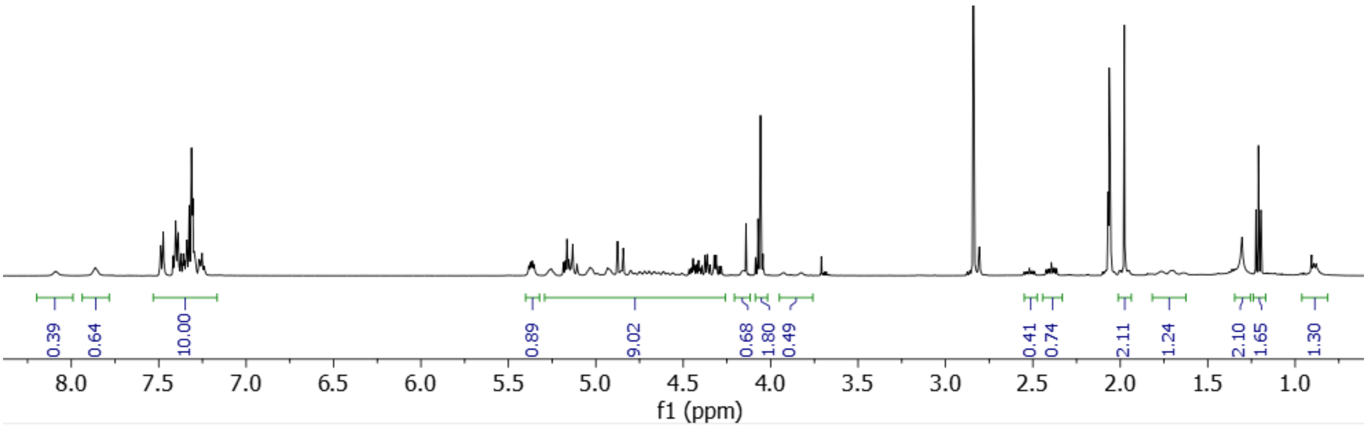

^{19}F NMR (470 MHz, Acetone-d_6)

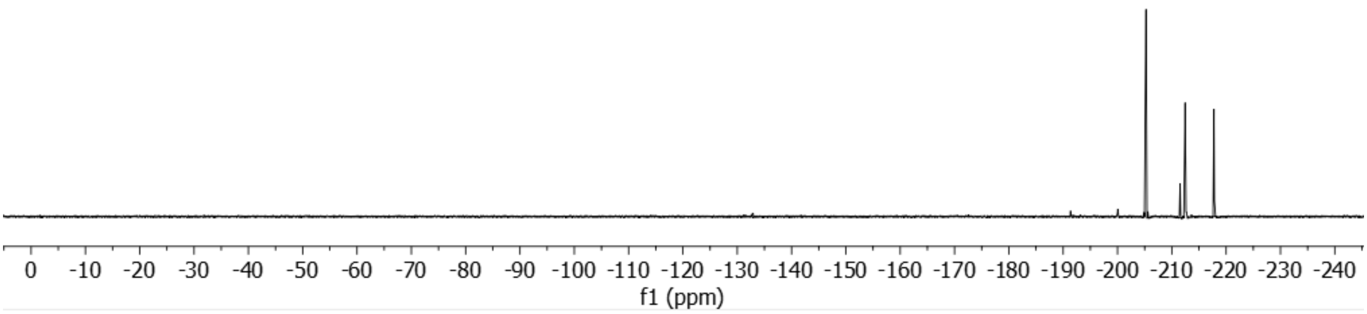

^{13}C NMR (126 MHz, Acetone-d_6)

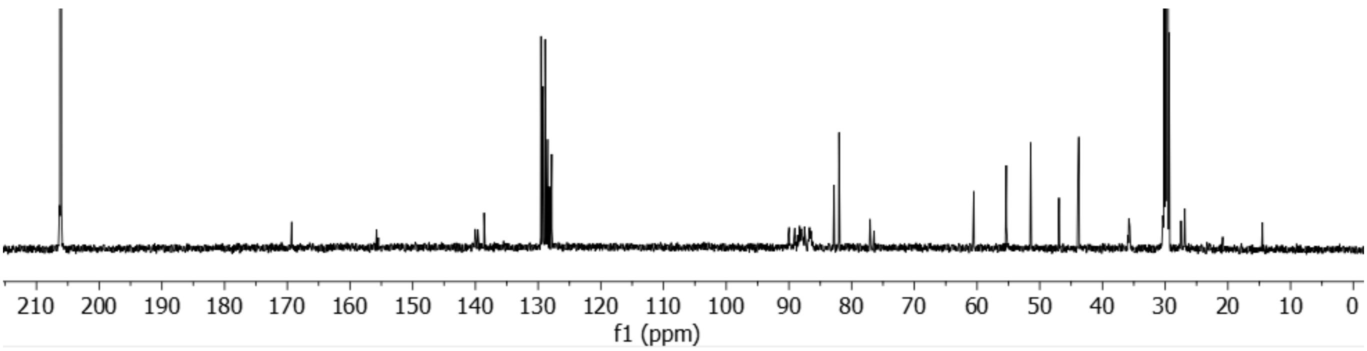

2-(N-allylacetamido)-N-benzyl-3-phenylpropanamide 26

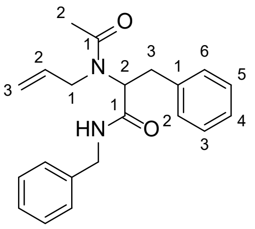

^{1}H NMR (500 MHz, $CDCl_3$)

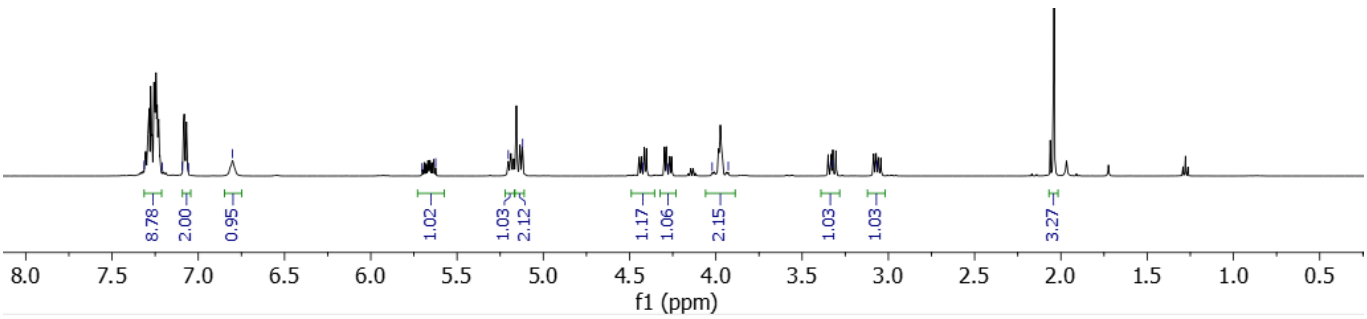

^{13}C NMR (126 MHz, $CDCl_3$)

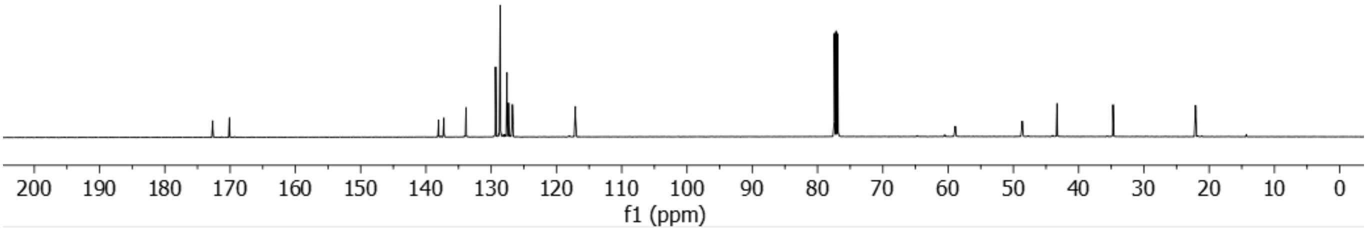

2-(N-allyl-2-(2-chlorophenyl)acetamido)-N-benzyl-3-phenylpropanamide 27

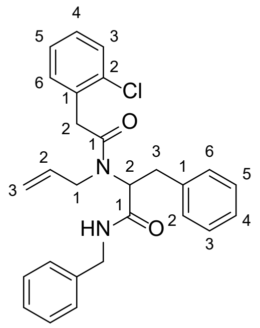

^{1}H NMR (500 MHz, $CDCl_3$)

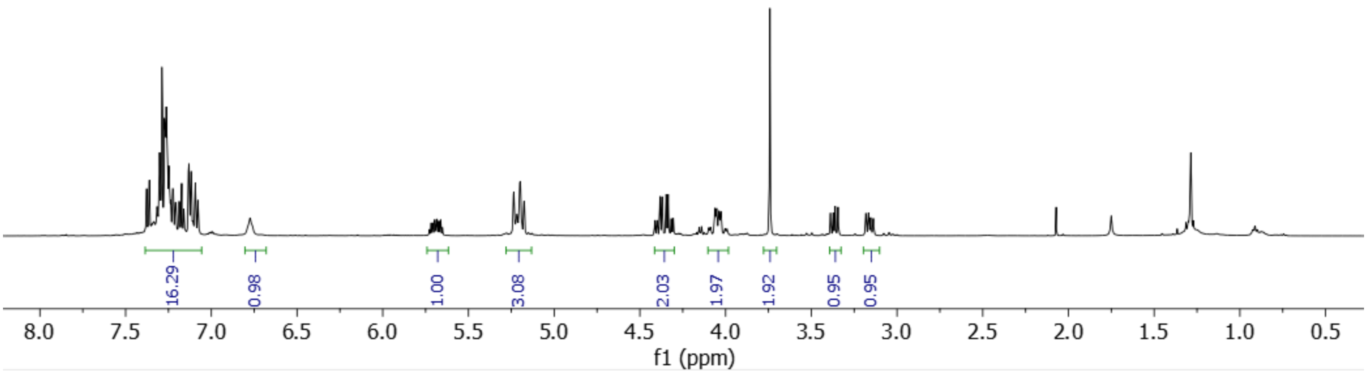

^{13}C NMR (126 MHz, $CDCl_3$)

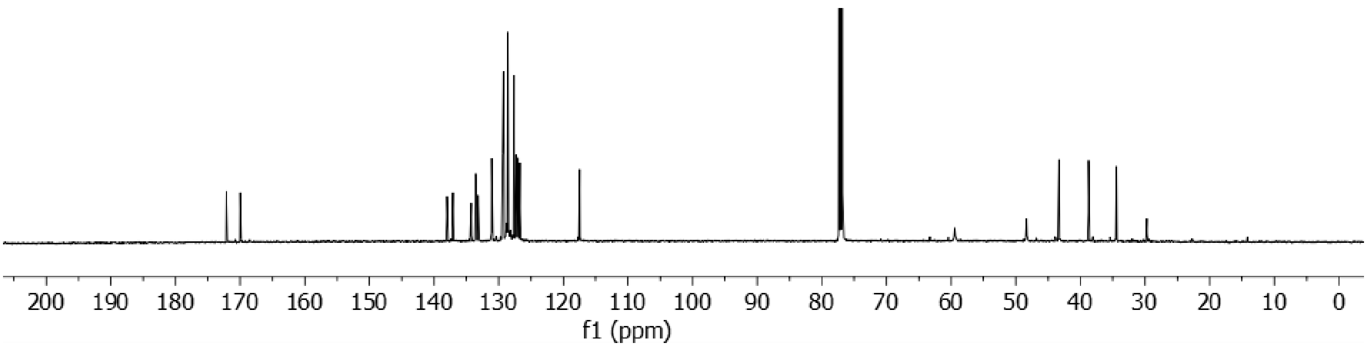

N-benzyl-2-(N-benzylacetamido)-3-phenylpropanamide 28

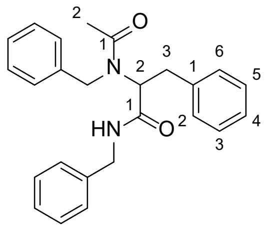

^{1}H NMR (500 MHz, $CDCl_3$)

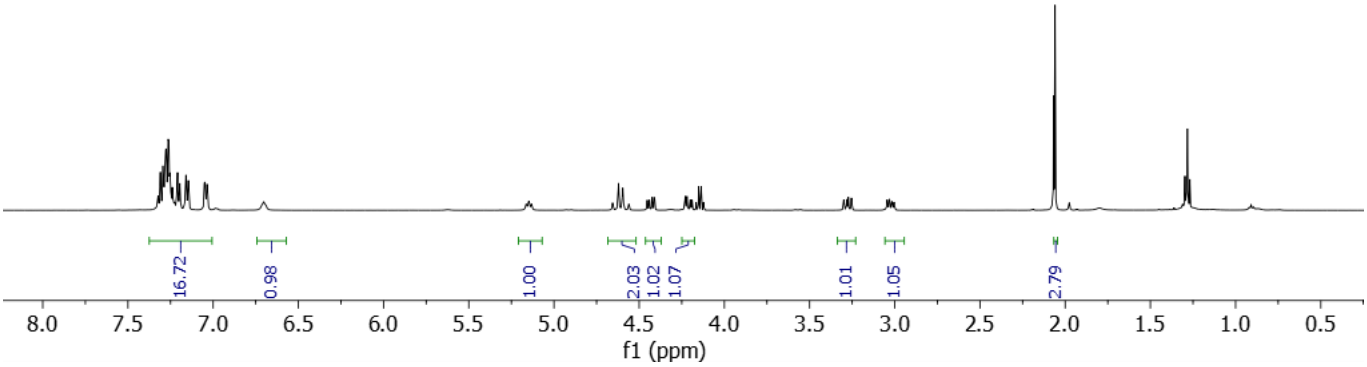

^{13}C NMR (126 MHz, $CDCl_3$)

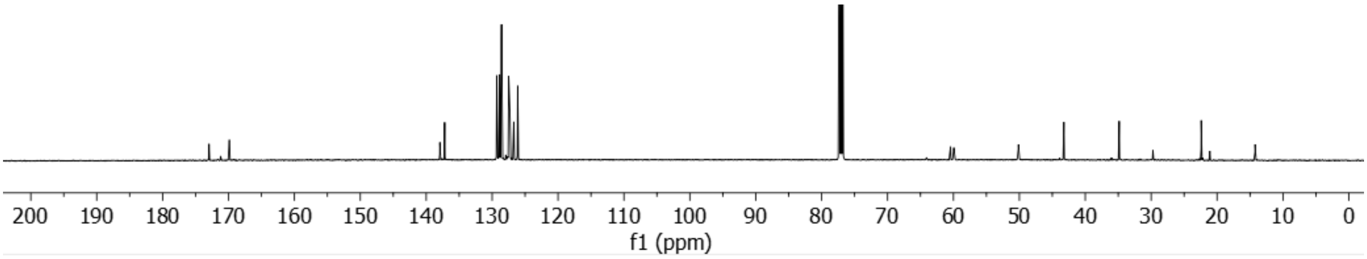

(1r,2R,3R,4s,5S,6S)-1-(2-bromoethyl)-2,3,4,5,6-pentafluorocyclohexane 29

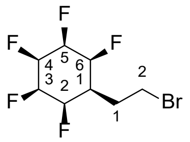

^{1}H NMR (400 MHz, Methanol-d_4)

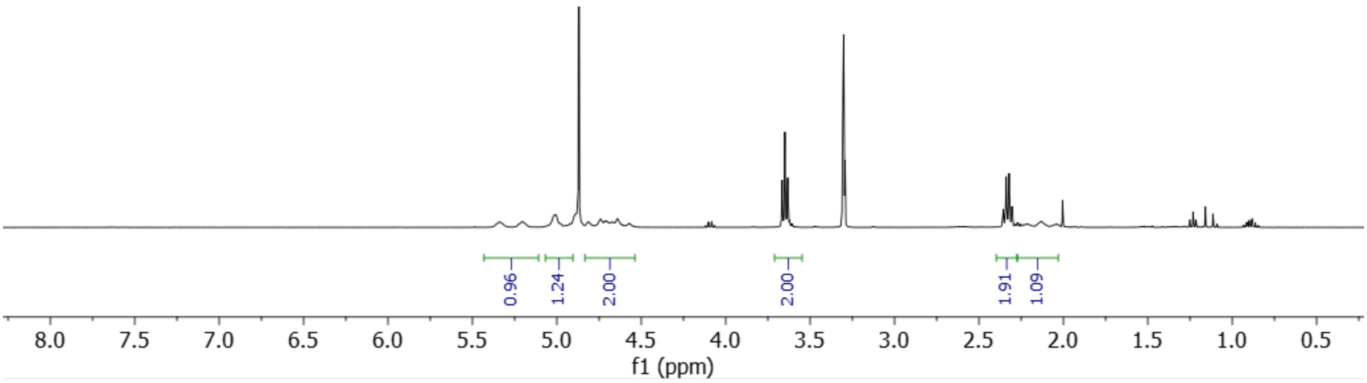

^{19}F NMR (377 MHz, Methanol-d_4)

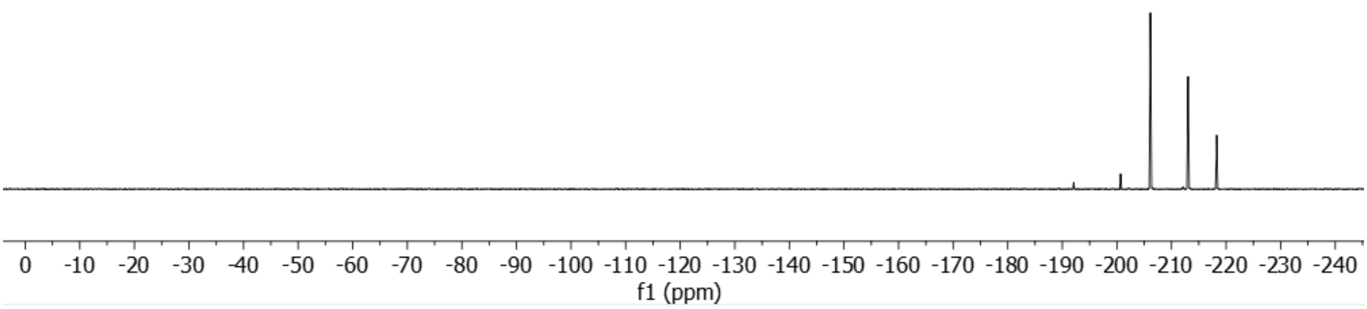

^{13}C NMR (101 MHz, Methanol-d_4)

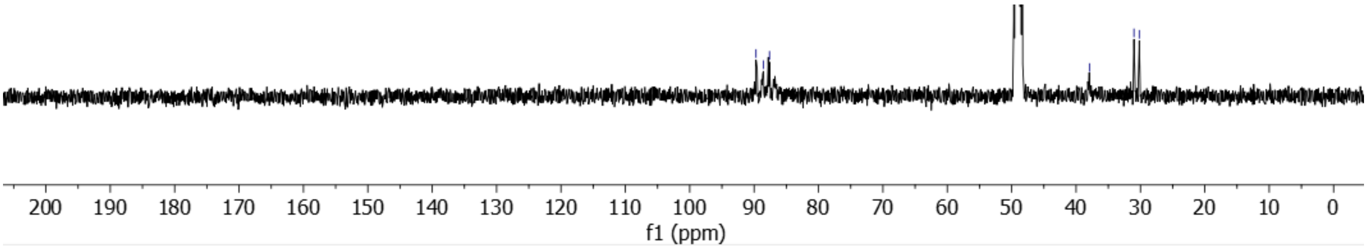

(1r,2R,3R,4s,5S,6S)-1-(2-azidoethyl)-2,3,4,5,6-pentafluorocyclohexane 14

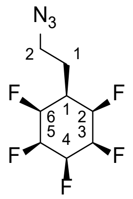

^{1}H NMR (500 MHz, Methanol-d_4)

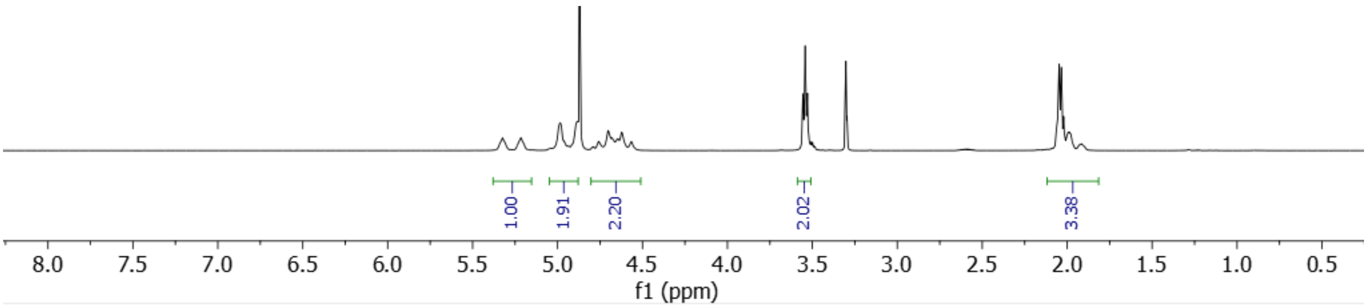

^{19}F NMR (471 MHz, Methanol-d_4)

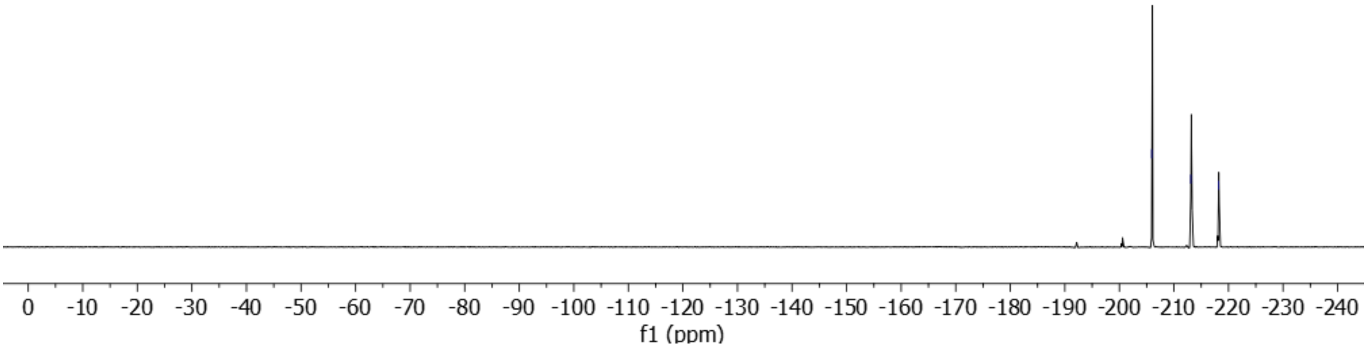

^{13}C NMR (126 MHz, Methanol-d_4)

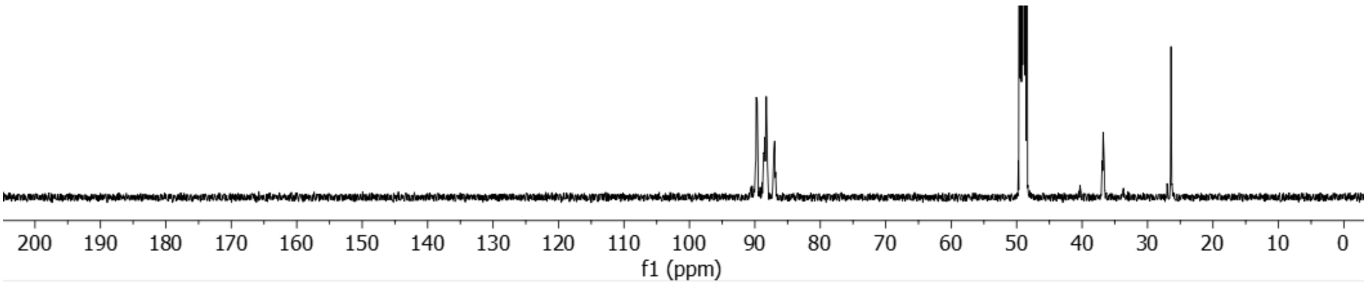

2-((1r,2R,3R,4s,5S,6S)-2,3,4,5,6-pentafluorocyclohexyl)ethan-1-amine 34

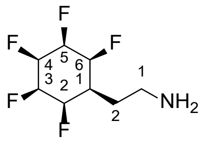

^{1}H NMR (500 MHz, Methanol-d_4)

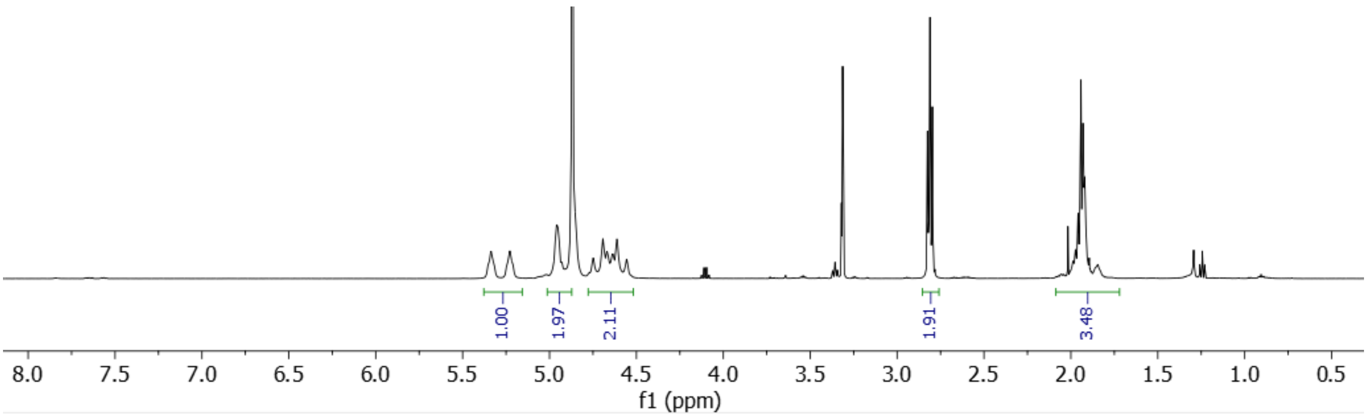

^{19}F NMR (471 MHz, Methanol-d_4)

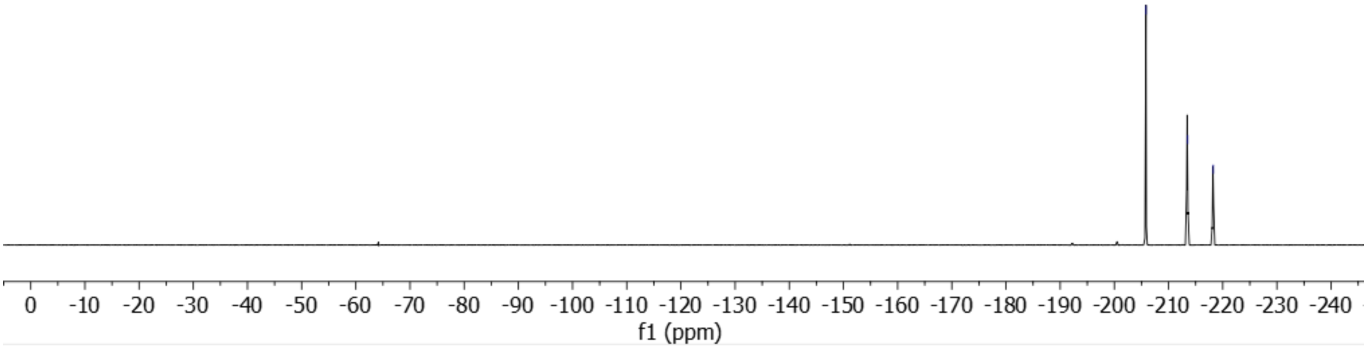

^{13}C NMR (126 MHz, Methanol-d_4)

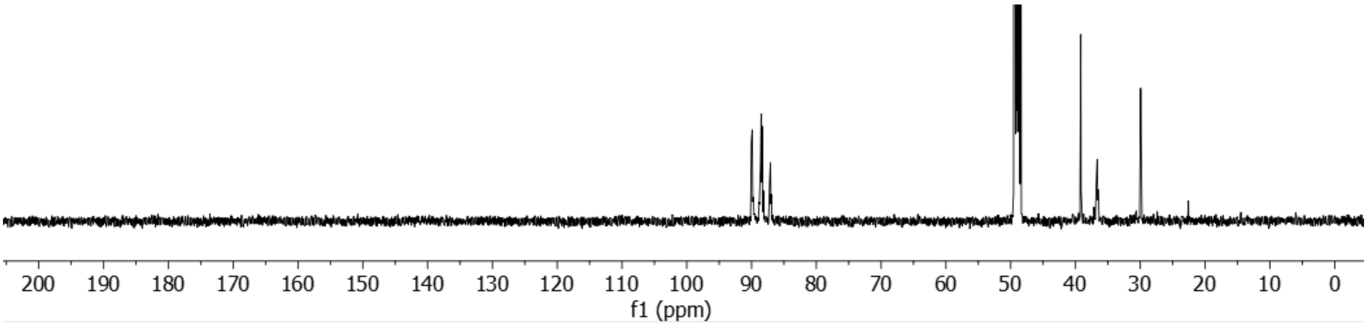

1-(2-((1r,2R,3R,4s,5S,6S)-2,3,4,5,6-pentafluorocyclohexyl)ethyl)-4-(p-tolyl)-1H-1,2,3-triazole 30

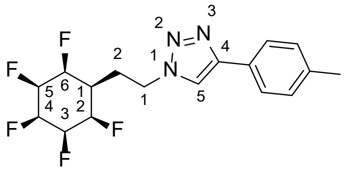

^{1}H NMR (500 MHz, Acetone-d_6)

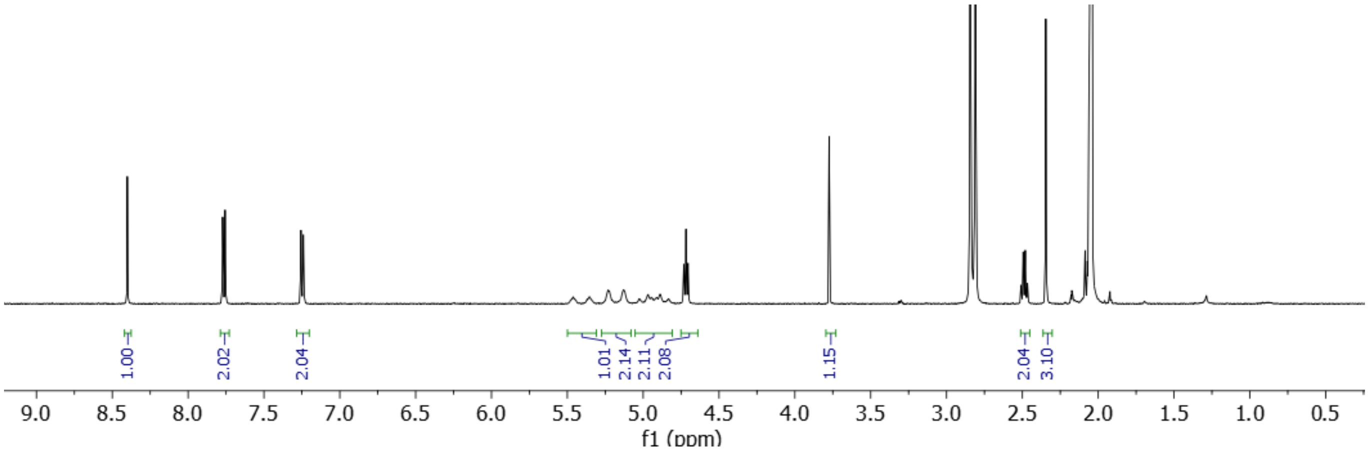

^{19}F NMR (470 MHz, Acetone-d_6)

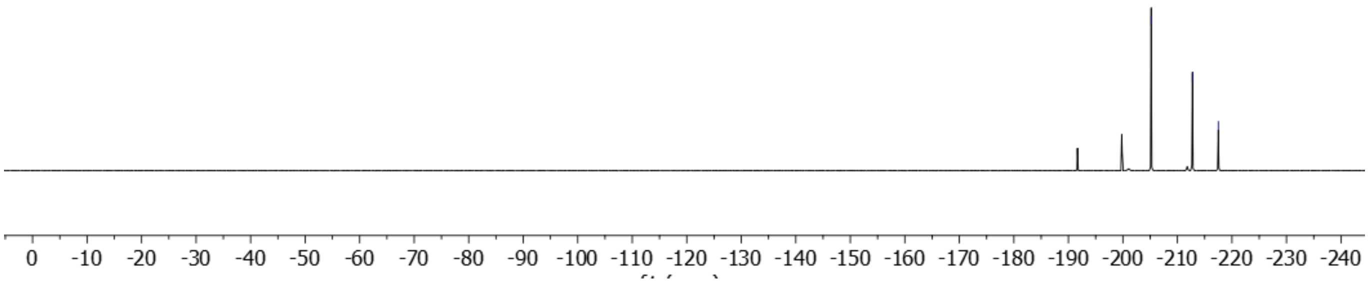

^{13}C NMR (126 MHz, Acetone-d_6)

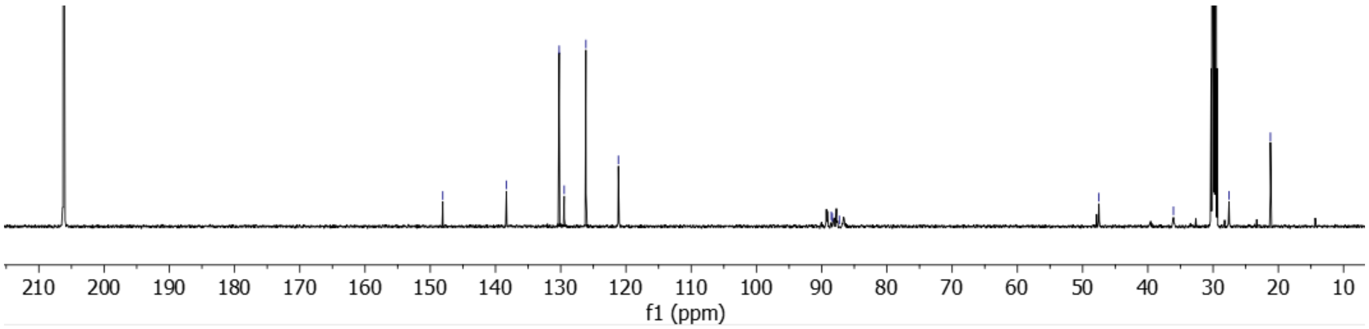

4-([1,1'-biphenyl]-4-yl)-1-(2-((1r,2R,3R,4s,5S,6S)-2,3,4,5,6-pentafluorocyclohexyl)ethyl)-1H-1,2,3-triazole 31

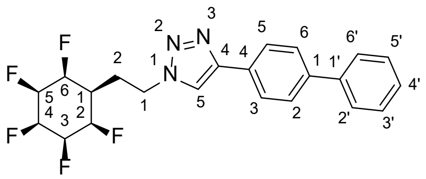

^{1}H NMR (700 MHz, Acetone-d_6)

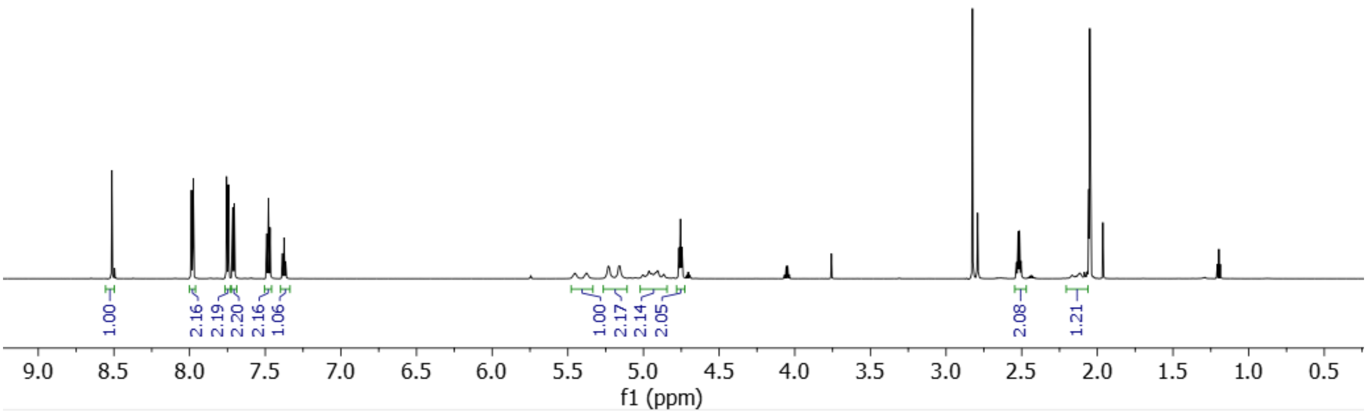

^{19}F NMR (659 MHz, Acetone-d_6)

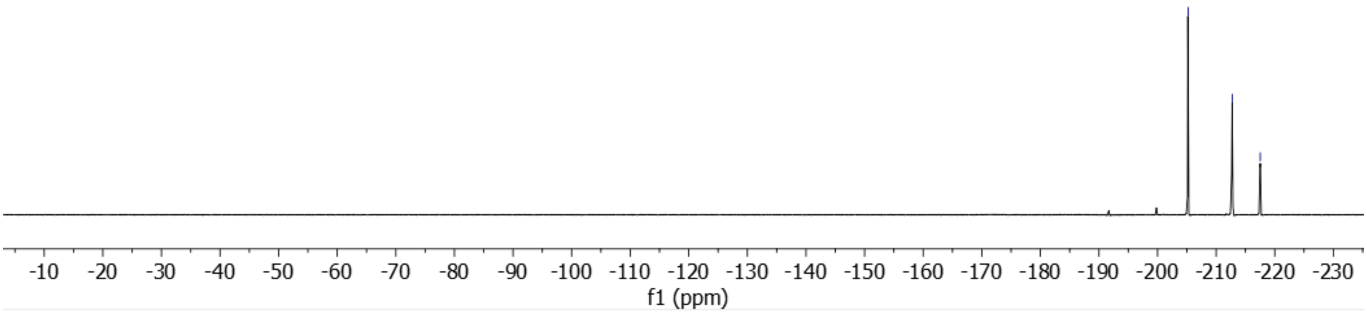

^{13}C NMR (176 MHz, Acetone-d_6)

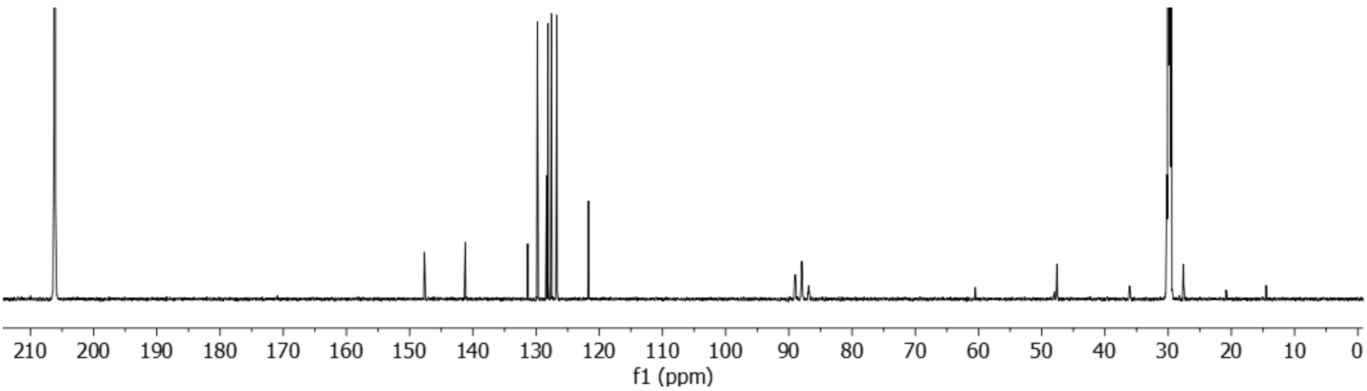

4-(1-(2-((1r,2R,3R,4s,5S,6S)-2,3,4,5,6-pentafluorocyclohexyl)ethyl)-1H-1,2,3-triazol-4-yl)benzoic acid 32

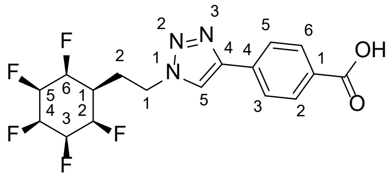

^{1}H NMR (500 MHz, DMSO-d_6)

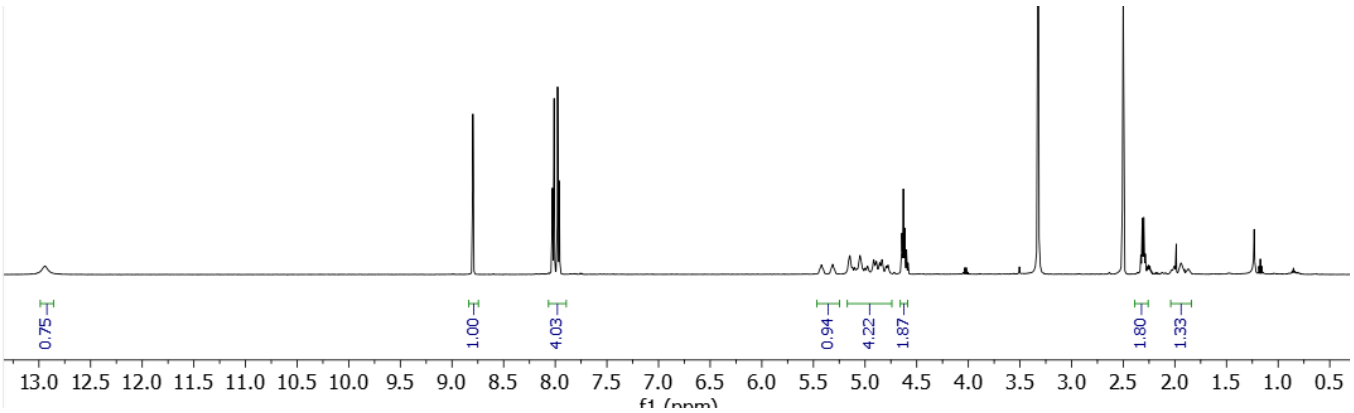

^{19}F NMR (470 MHz, DMSO-d_6)

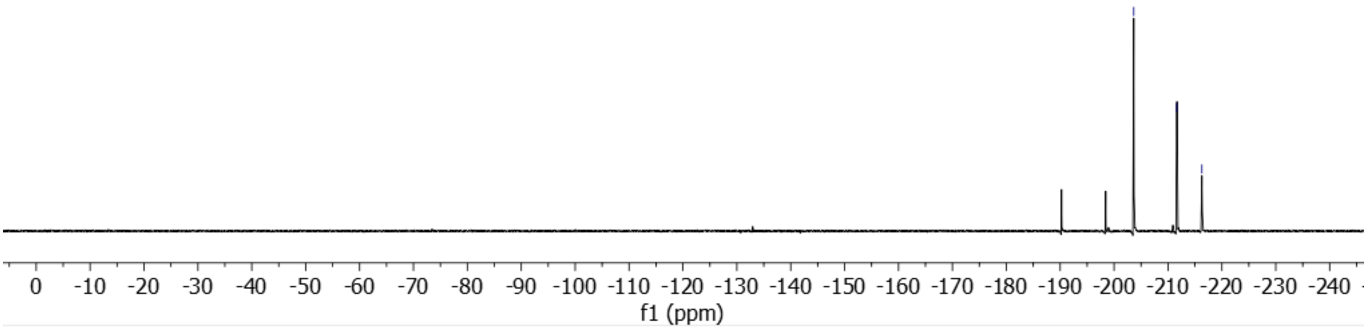

^{13}C NMR (126 MHz, DMSO-d_6)

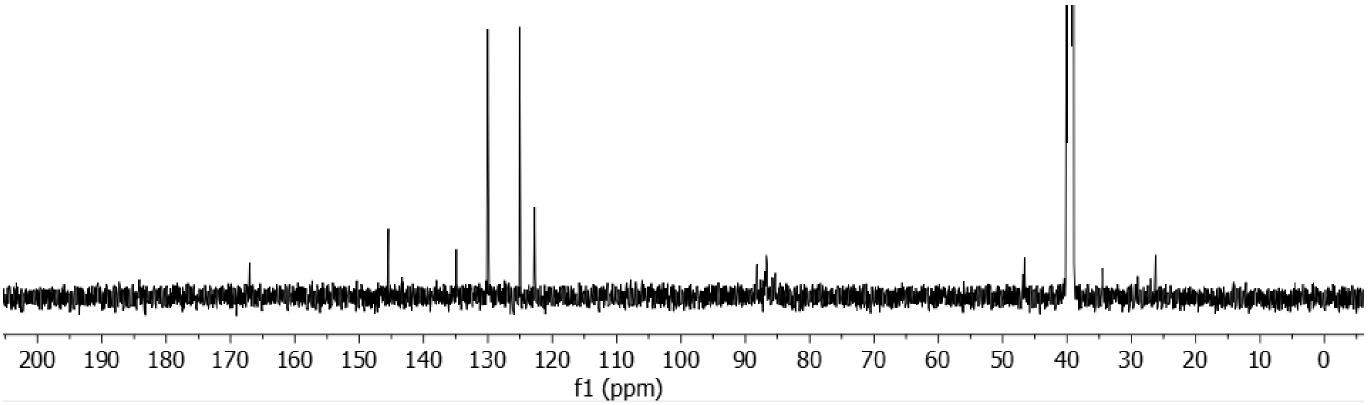

1,4-bis(1-(2-((1r,2R,3R,4s,5S,6S)-2,3,4,5,6-pentafluorocyclohexyl)ethyl)-1H-1,2,3-triazol-4-yl)benzene 33

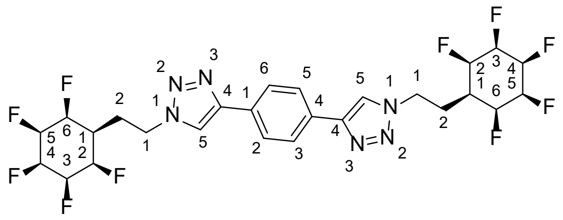

^{1}H NMR (500 MHz, DMSO-d_6)

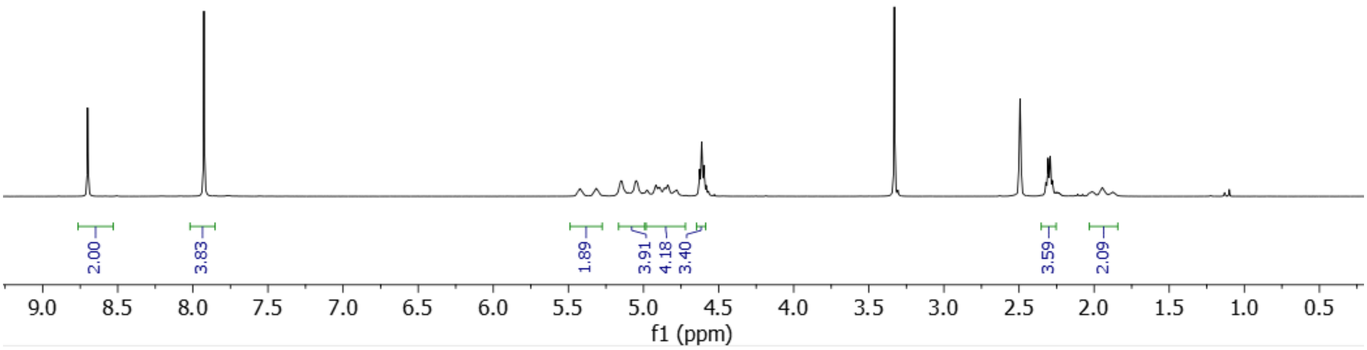

^{19}F NMR (471 MHz, DMSO-d_6)

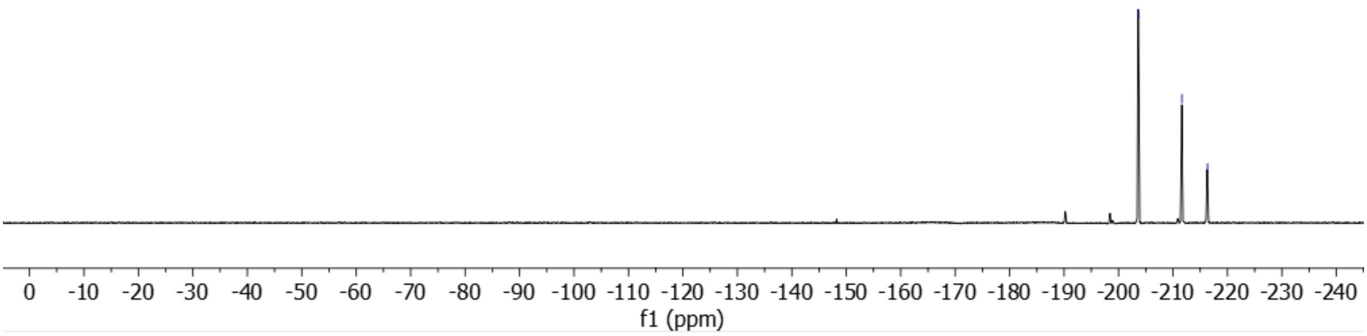

^{13}C NMR (126 MHz, DMSO-d_6)

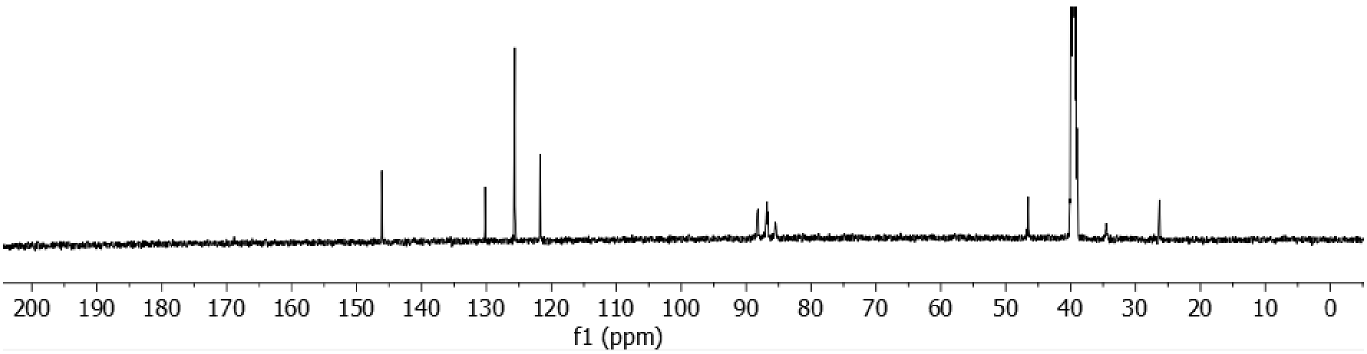

(*S*)-2-((tert-butoxycarbonyl)amino)-3-((1*r*,2*R*,3*R*,4*R*,5*S*,6*S*)-2,3,4,5,6-pentafluorocyclohexyl)propanoic acid 37

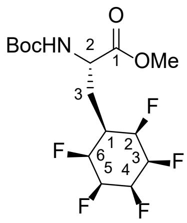

^{1}H NMR (400 MHz, Methanol-d_4)

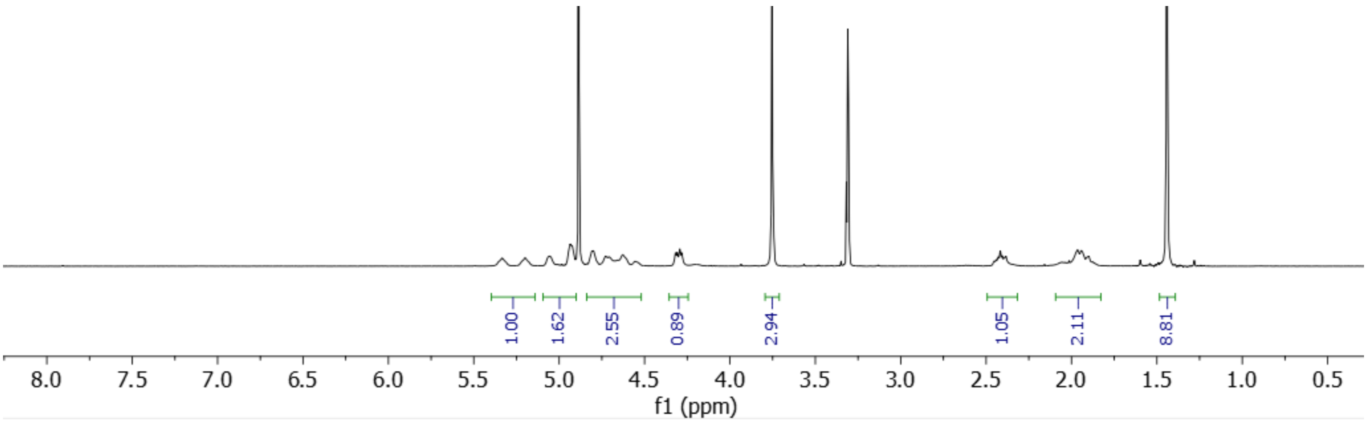

^{13}C NMR (126 MHz, Methanol-d_4)

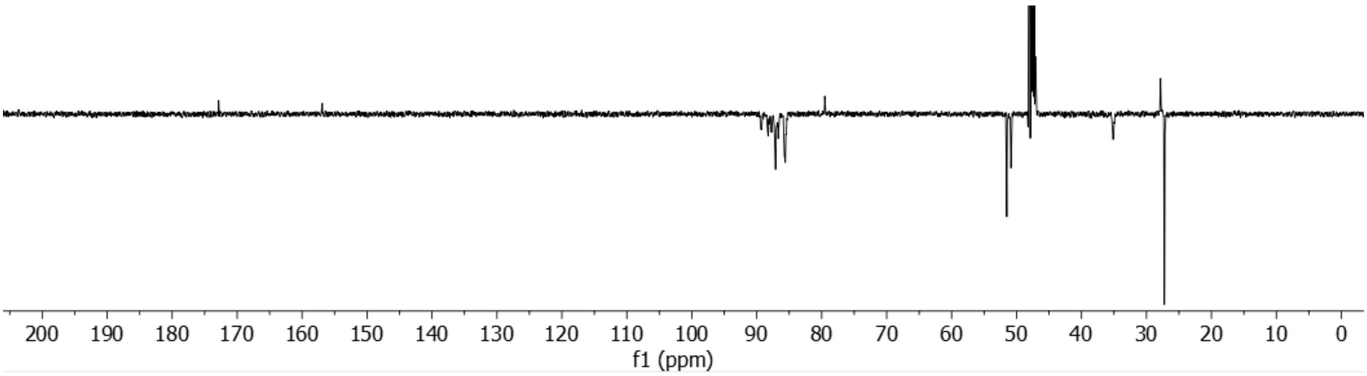

^{19}F NMR (376 MHz, Methanol-d_4)

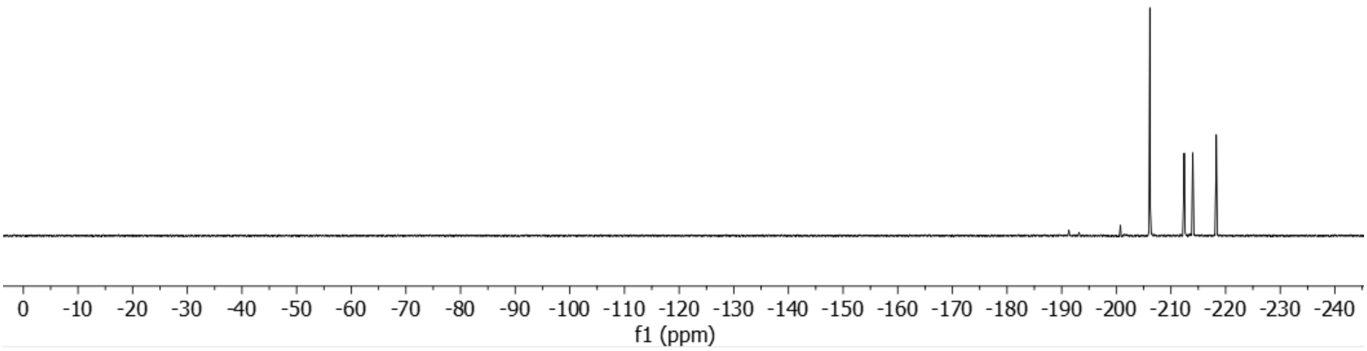

(2*S*)-2-((*tert*-Butoxycarbonyl)amino)-3-(2,3,4,5,6-pentafluorocyclohexyl)propanoic acid 38

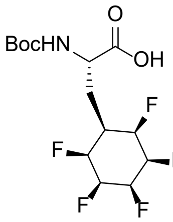

^{1}H NMR (400 MHz, Methanol-d_4)

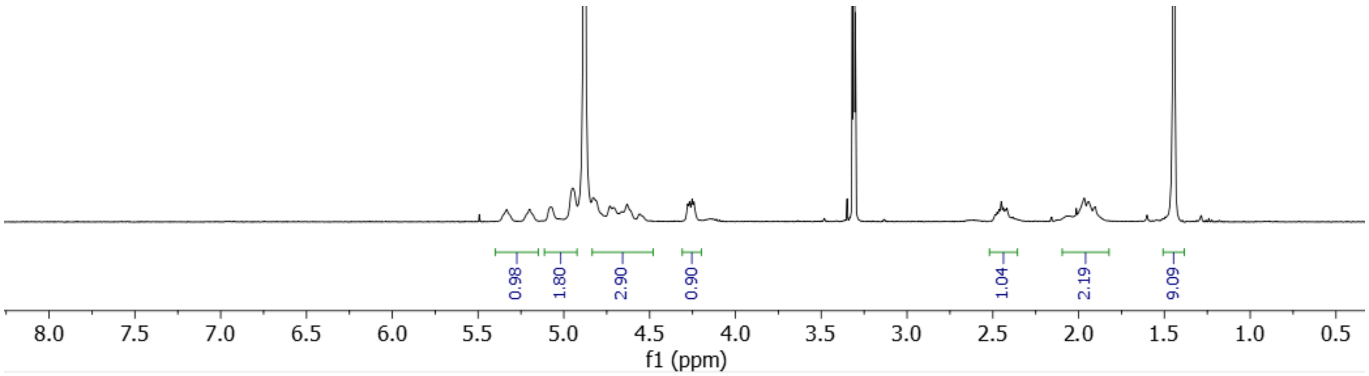

^{13}C NMR (126 MHz, Methanol-d_4)

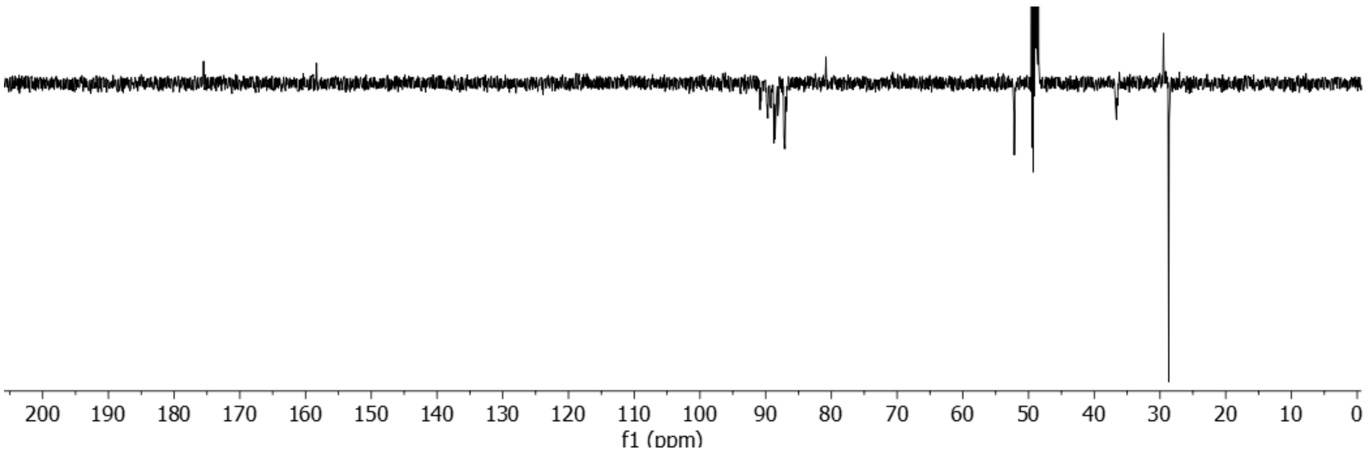

^{19}F NMR (377 MHz, Methanol-d_4)

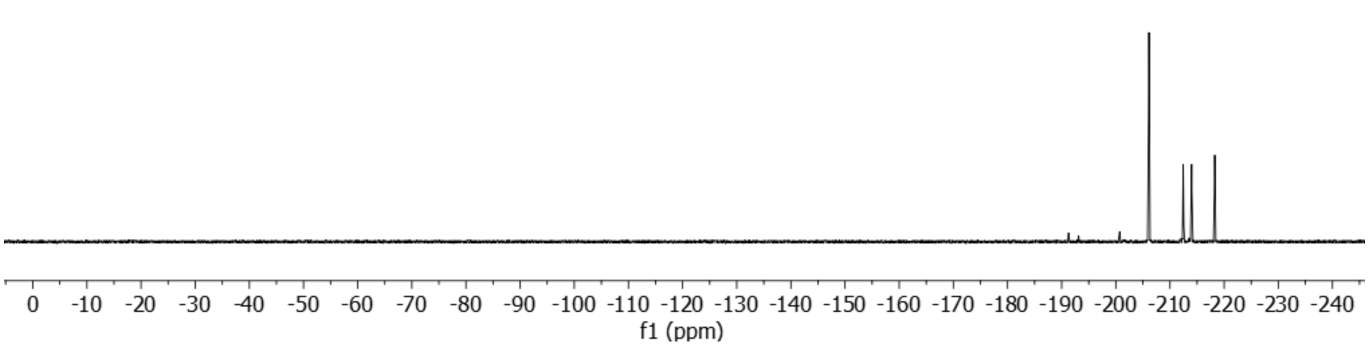

(S)-2-amino-3-((1r,2R,3R,4R,5S,6S)-2,3,4,5,6-pentafluorocyclohexyl)propanoic acid 15

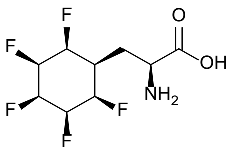

^{1}H NMR (700 MHz, DMSO-d_6)

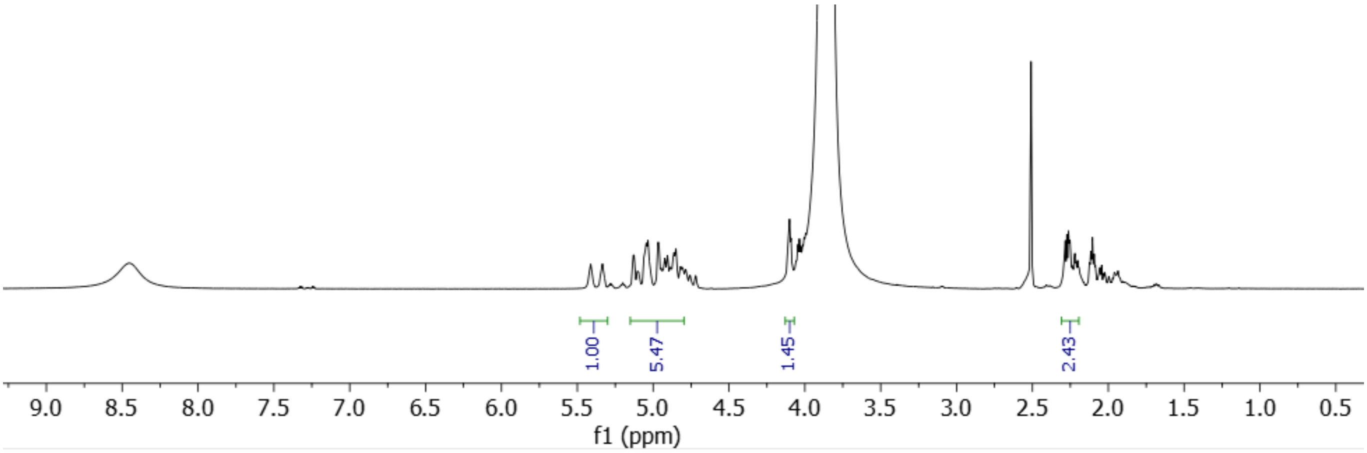

^{13}C NMR (176 MHz, DMSO-d_6)

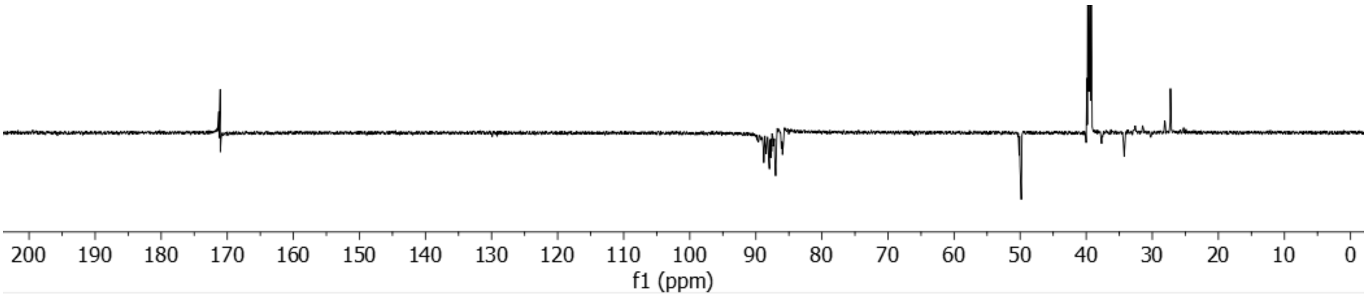

^{19}F NMR (659 MHz, DMSO-d_6)

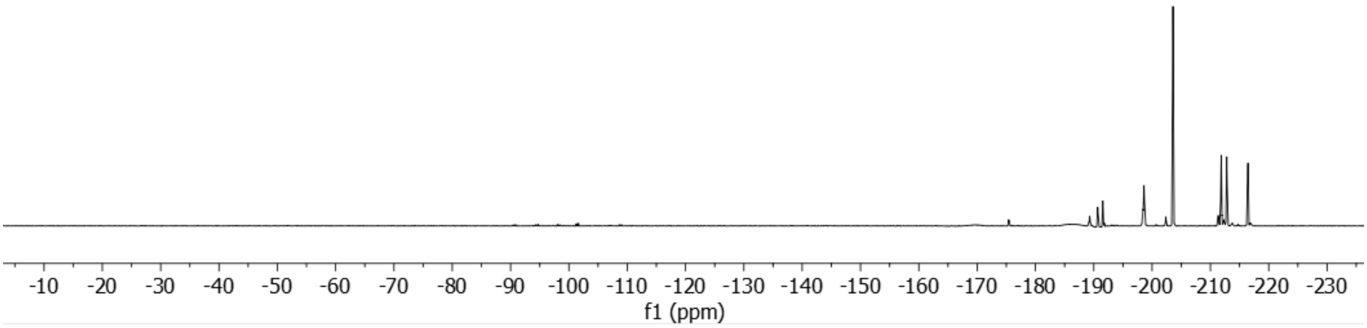

Methyl (*S*)-2-((*S*)-2-((*tert*-butoxycarbonyl)amino)-3-((1*r*,2*R*,3*R*,4*R*,5*S*,6*S*)-2,3,4,5,6-pentafluorocyclohexyl)propanamido)-3-((*S*)-2-oxopyrrolidin-3-yl)propanoate (40)

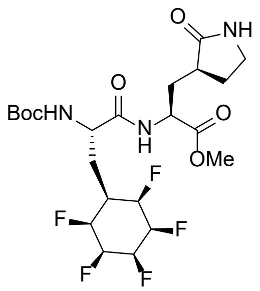

^{1}H NMR (400 MHz, Methanol-d_4)

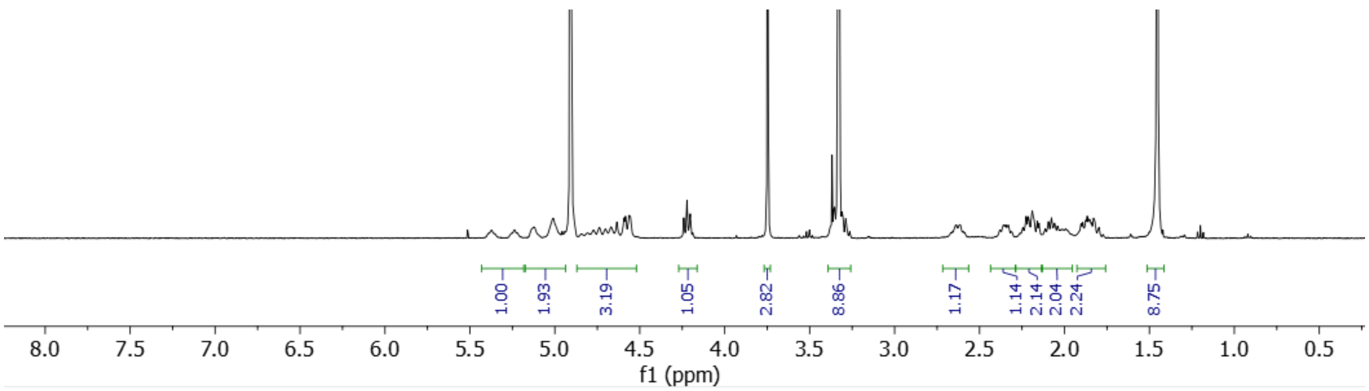

^{13}C NMR (126 MHz, Methanol-d_4)

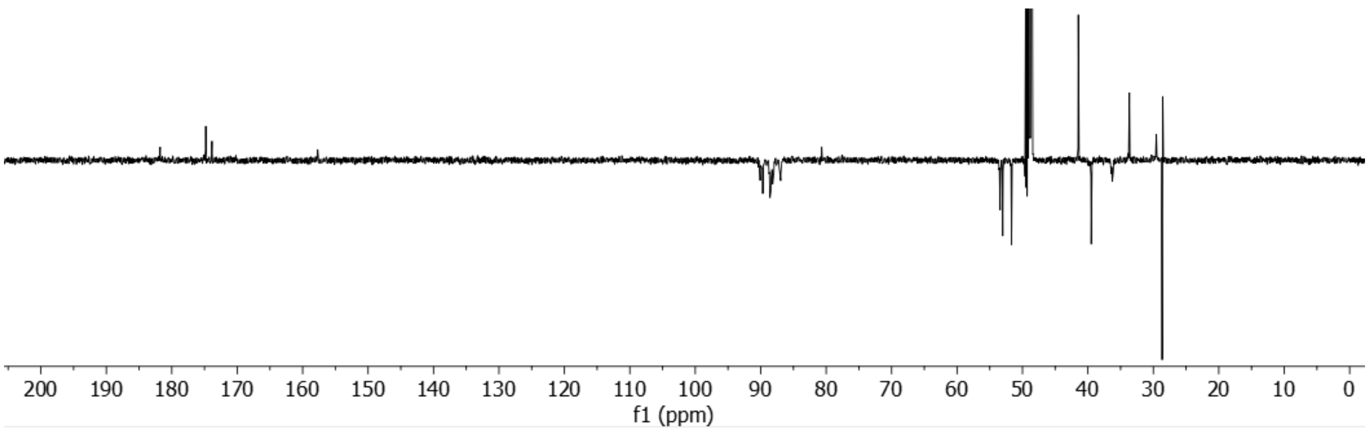

^{19}F NMR (470 MHz, $CDCl_3$)

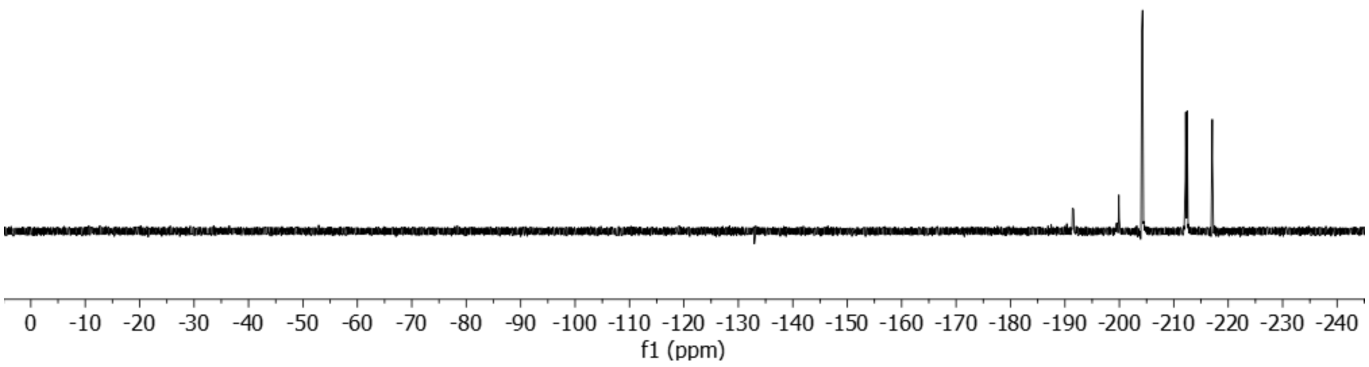

Methyl (*S*)-2-((*S*)-2-cinnamamido-3-((1*r*,2*R*,3*R*,4*R*,5*S*,6*S*)-2,3,4,5,6-pentafluorocyclohexyl)propanamido)-3-((*S*)-2-oxopyrrolidin-3-yl)propanoate (43)

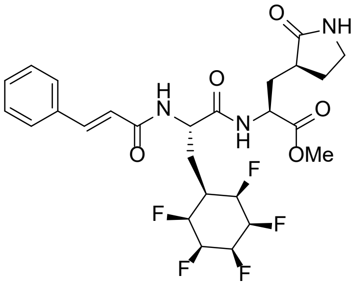

^{1}H NMR (400 MHz, Methanol-d_4)

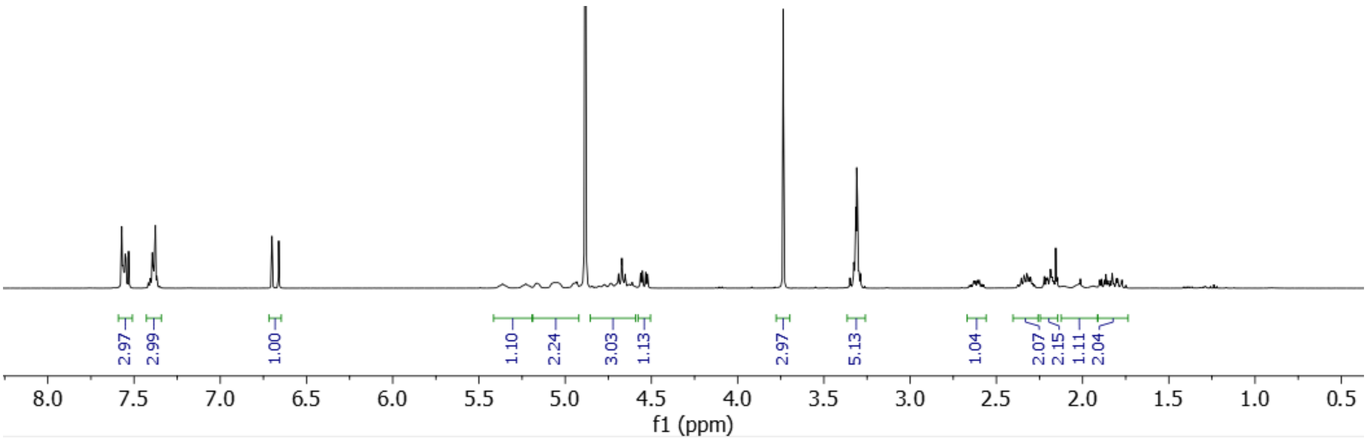

^{13}C NMR (126 MHz, Acetone-d_6)

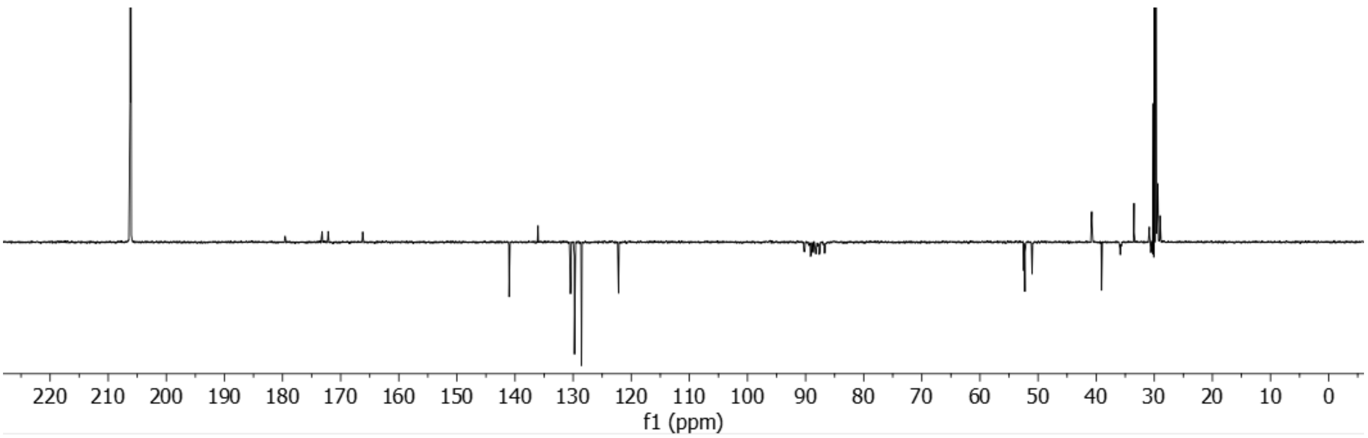

^{19}F NMR (376 MHz, Methanol-d_4)

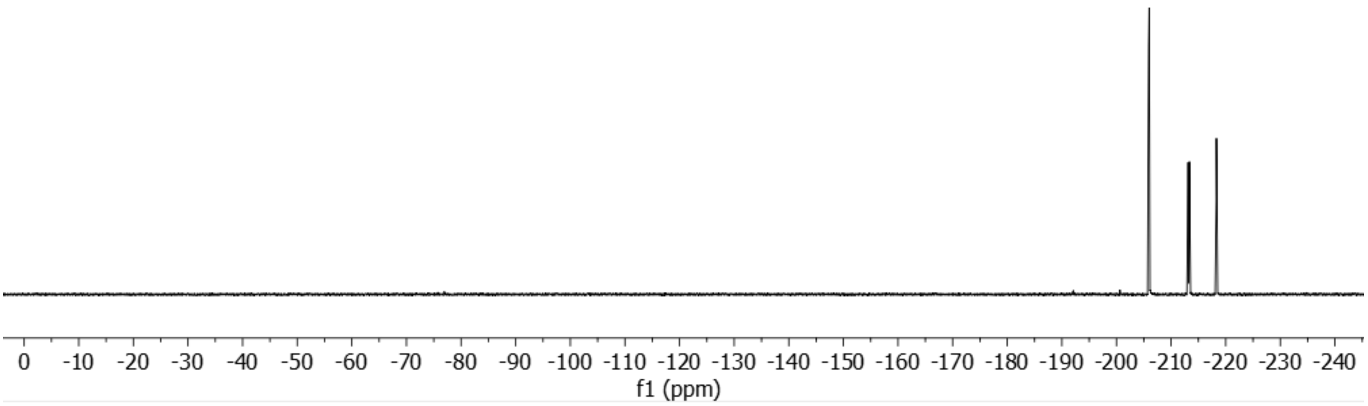

N-((*S*)-1-(((*S*)-1-hydroxy-3-((*S*)-2-oxopyrrolidin-3-yl)propan-2-yl)amino)-1-oxo-3-((1*r*,2*R*,3*R*,4*R*,5*S*,6*S*)-2,3,4,5,6-pentafluorocyclohexyl)propan-2-yl)cinnamamide (44)

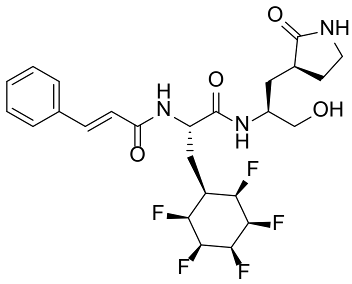

^{1}H NMR (500 MHz, Acetone-d_6)

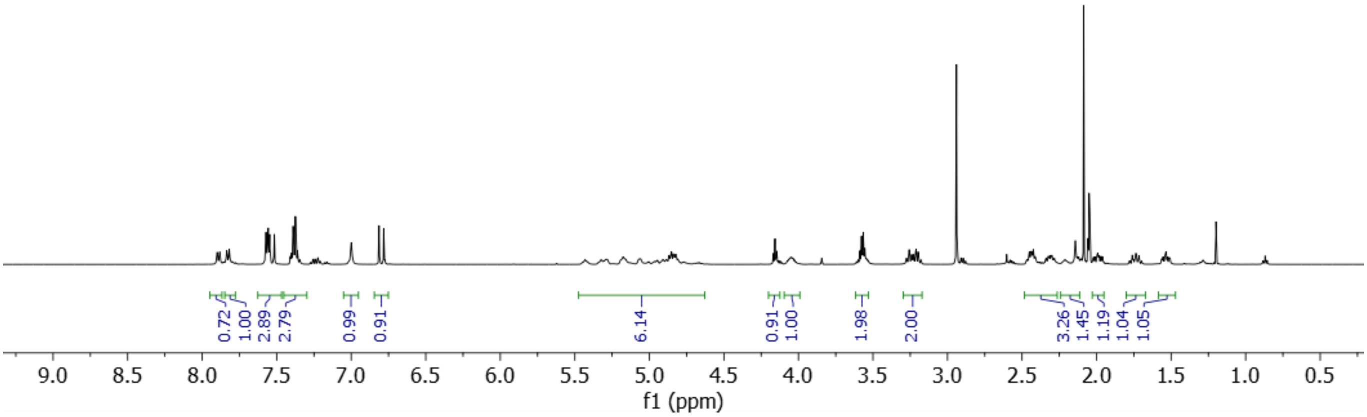

^{13}C NMR (126 MHz, Acetone-d_6)

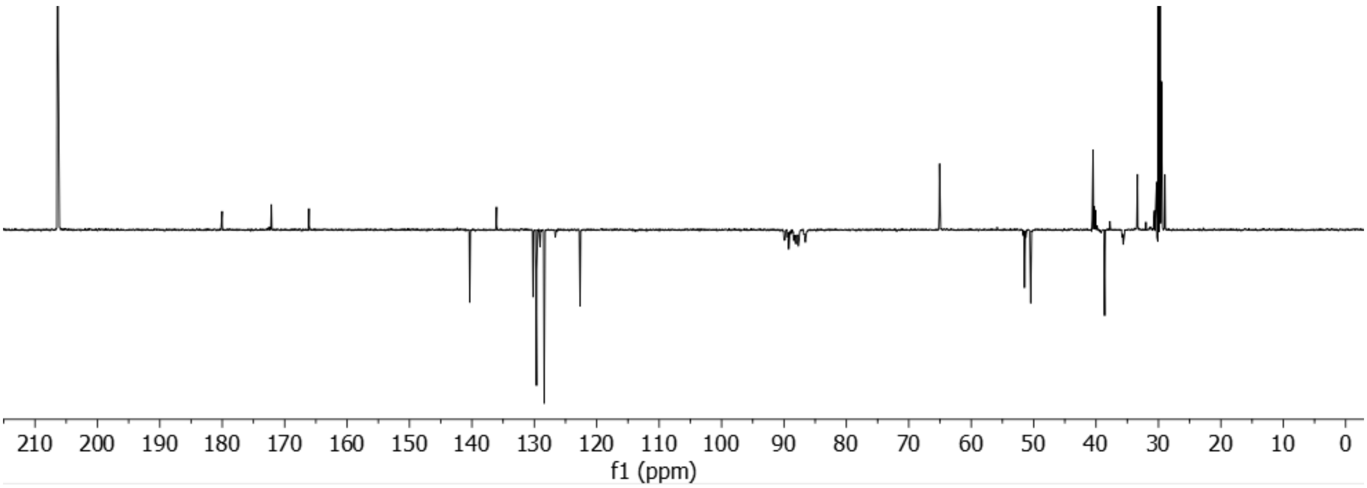

^{19}F NMR (376 MHz, Acetone-d_6)

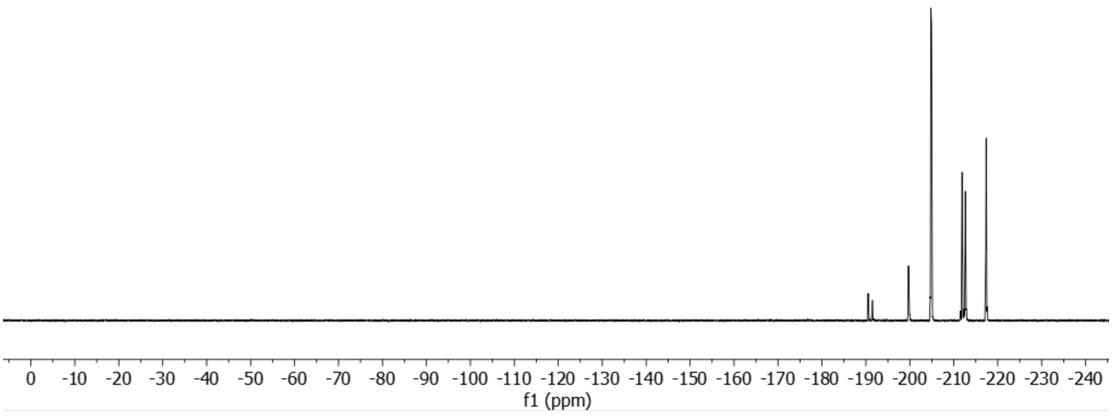

N-((*S*)-1-oxo-1-(((*S*)-1-oxo-3-((*S*)-2-oxopyrrolidin-3-yl)propan-2-yl)amino)-3-((1*r*,2*R*,3*R*,4*R*,5*S*,6*S*)-2,3,4,5,6-pentafluorocyclohexyl)propan-2-yl)cinnamamide (45)

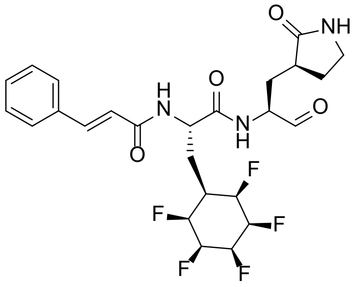

^{1}H NMR (500 MHz, Acetone-d_6)

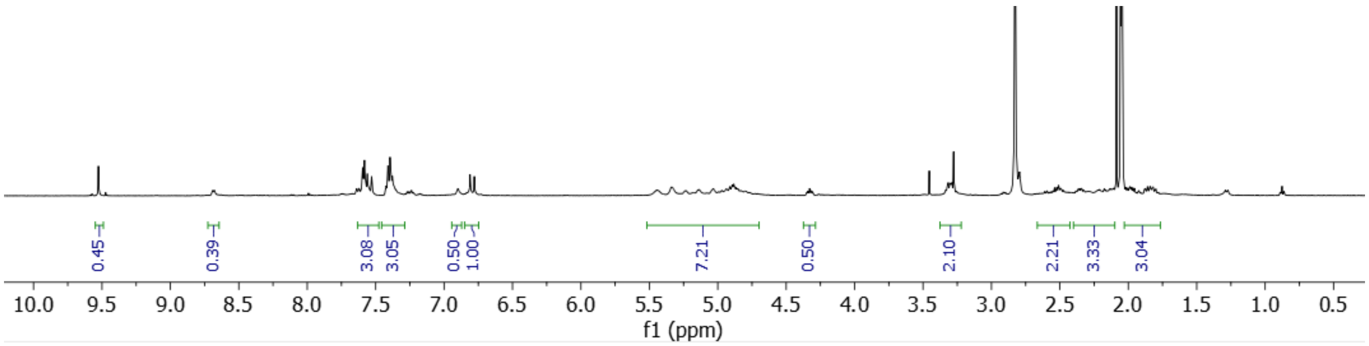

^{19}F NMR (470 MHz, Acetone-d_6)

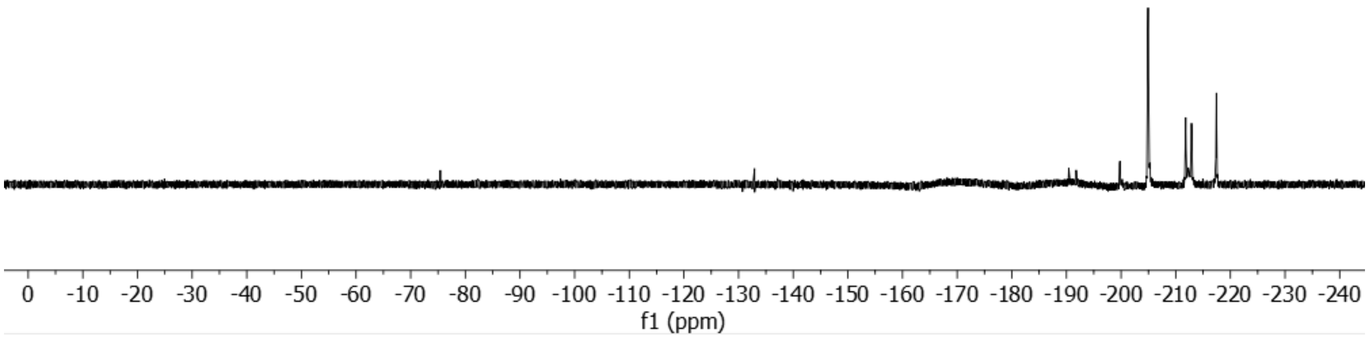

^{13}C NMR (126 MHz, Acetonitrile-d_3)

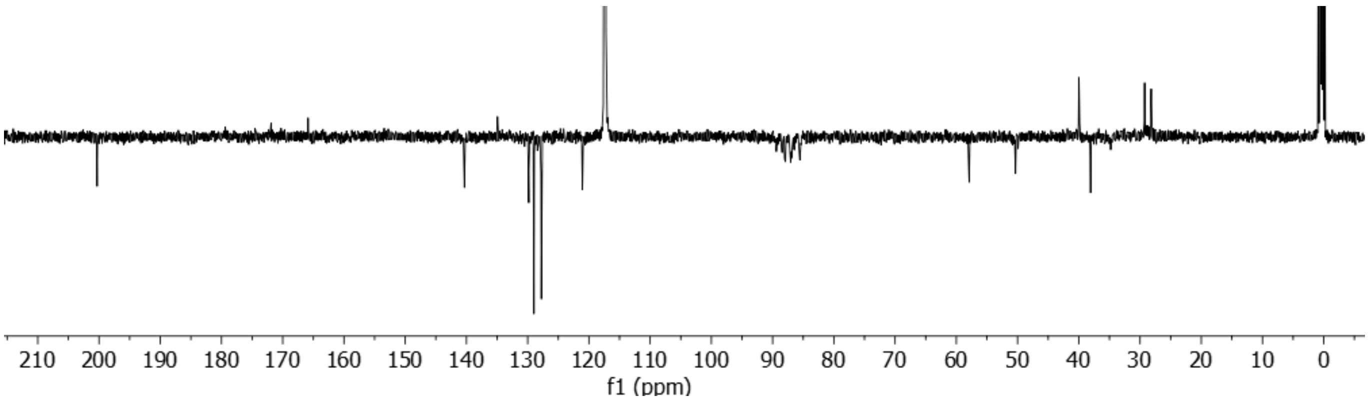

Methyl (*S*)-2-((*S*)-2-(1H-indole-2-carboxamido)-3-((1*r*,2*R*,3*R*,4*R*,5*S*,6*S*)-2,3,4,5,6-pentafluorocyclohexyl)propanamido)-3-((*S*)-2-oxopyrrolidin-3-yl)propanoate (46)

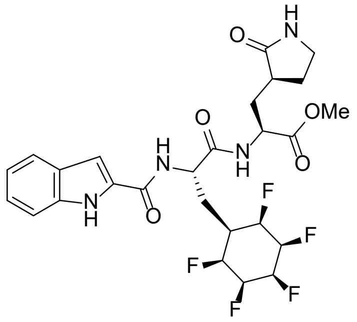

^{1}H NMR (400 MHz, Methanol-d_4)

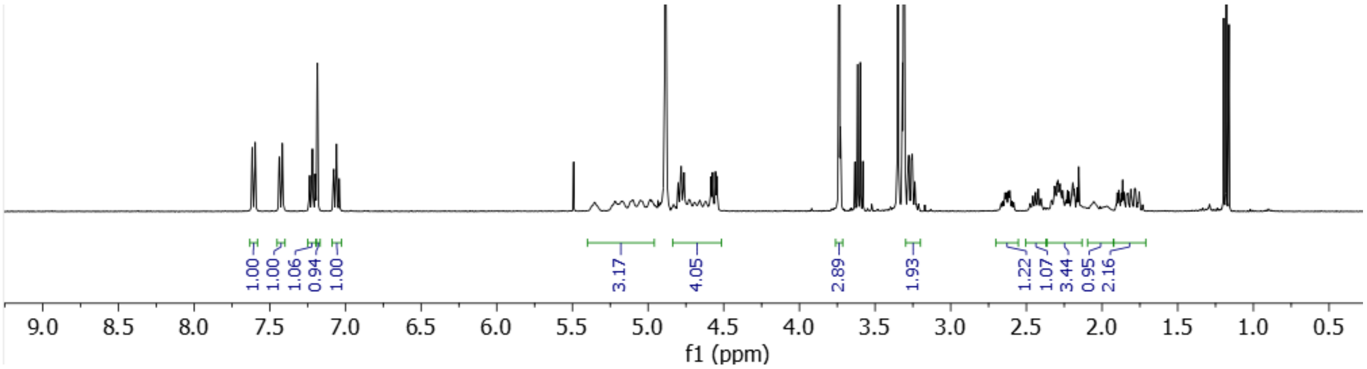

^{13}C NMR (126 MHz, Methanol-d_4)

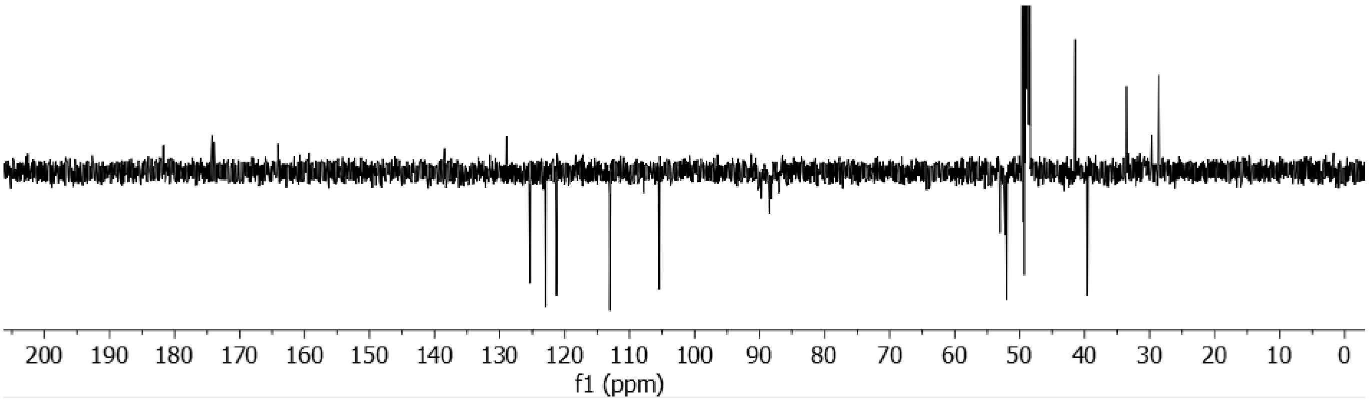

^{19}F NMR (376 MHz, Methanol-d_4)

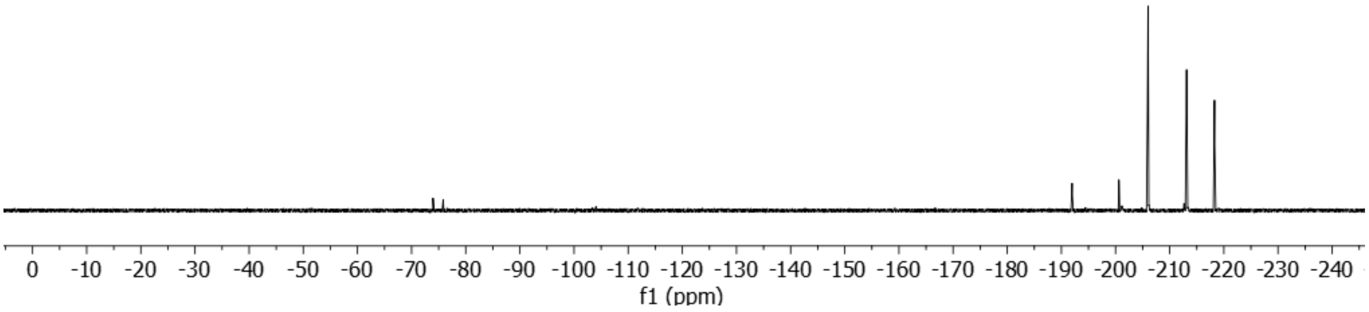

N-((*S*)-1-(((*S*)-1-hydroxy-3-((*S*)-2-oxopyrrolidin-3-yl)propan-2-yl)amino)-1-oxo-3-((1*r*,2*R*,3*R*,4*R*,5*S*,6*S*)-2,3,4,5,6-pentafluorocyclohexyl)propan-2-yl)-1H-indole-2-carboxamide (47)

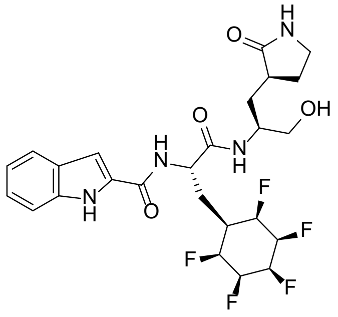

^{1}H NMR (400 MHz, Methanol-d_4)

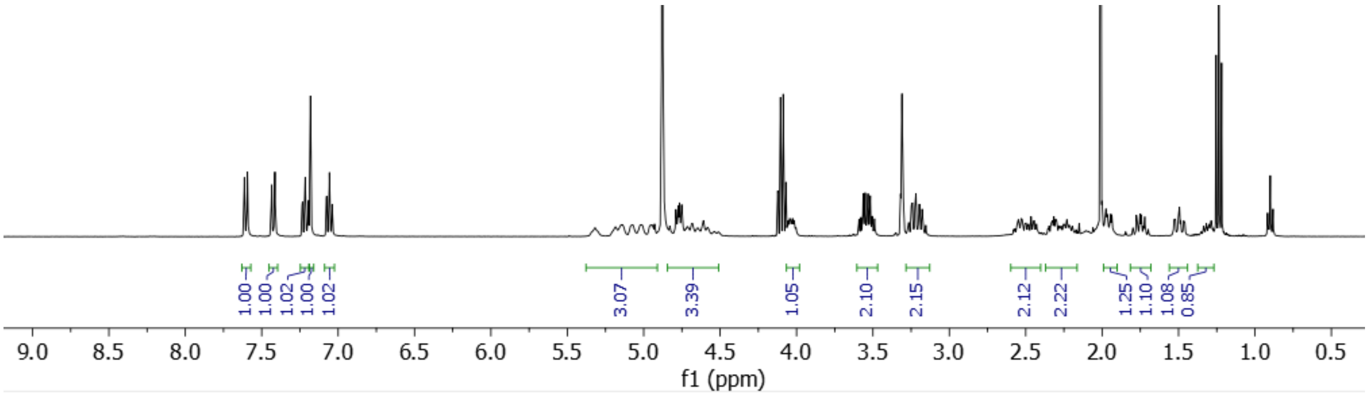

^{13}C NMR (126 MHz, Methanol-d_4)

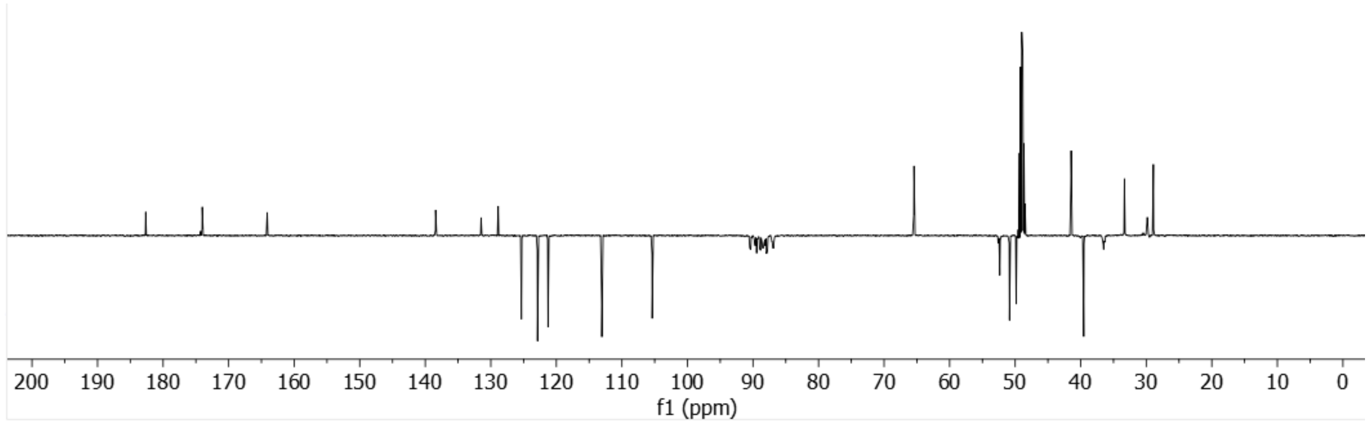

^{19}F NMR (377 MHz, Methanol-d_4)

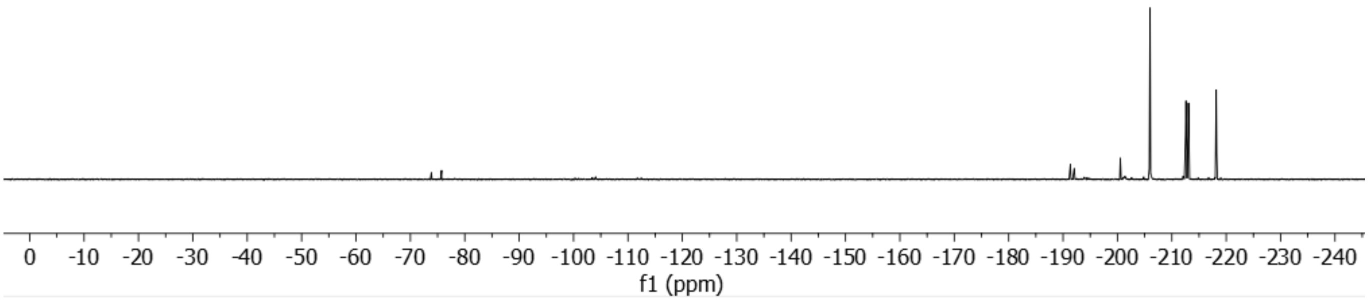

N-((*S*)-1-oxo-1-(((*S*)-1-oxo-3-((*S*)-2-oxopyrrolidin-3-yl)propan-2-yl)amino)-3-((1*r*,2*R*,3*R*,4*R*,5*S*,6*S*)-2,3,4,5,6-pentafluorocyclohexyl)propan-2-yl)-1H-indole-2-carboxamide (48)

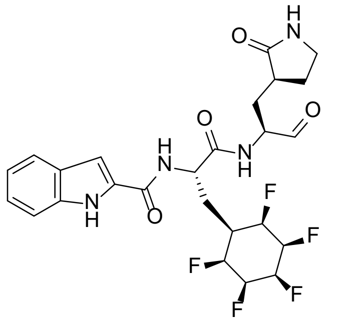

^{1}H NMR (400 MHz, Acetone-d_6)

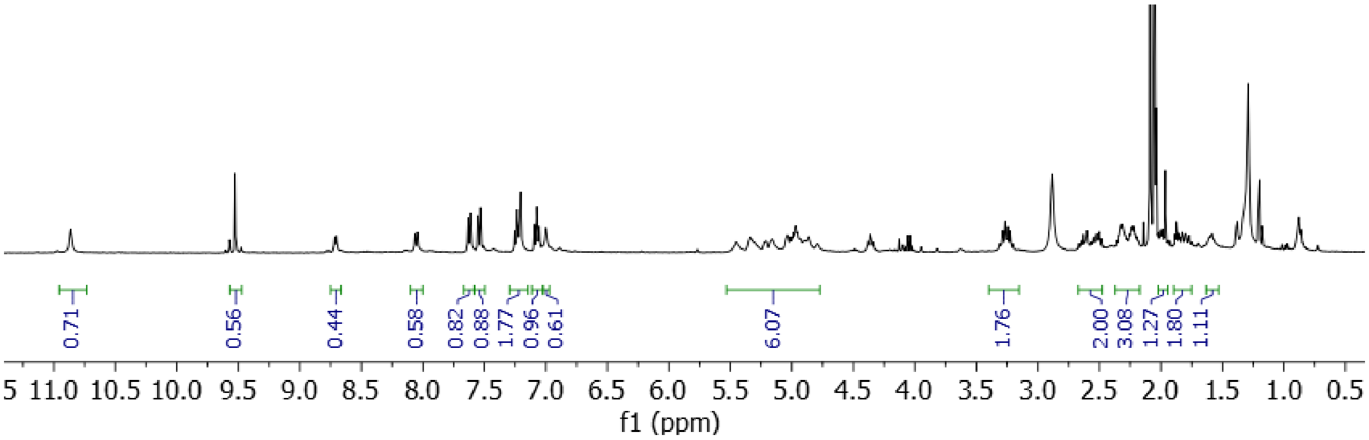

^{19}F NMR (376 MHz, Acetone-d_6)

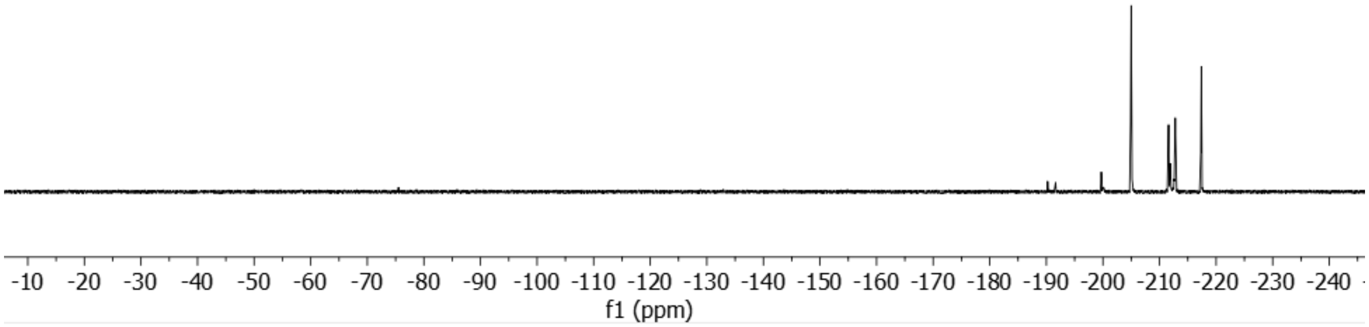

^{13}C NMR (126 MHz, Acetone-d_6)

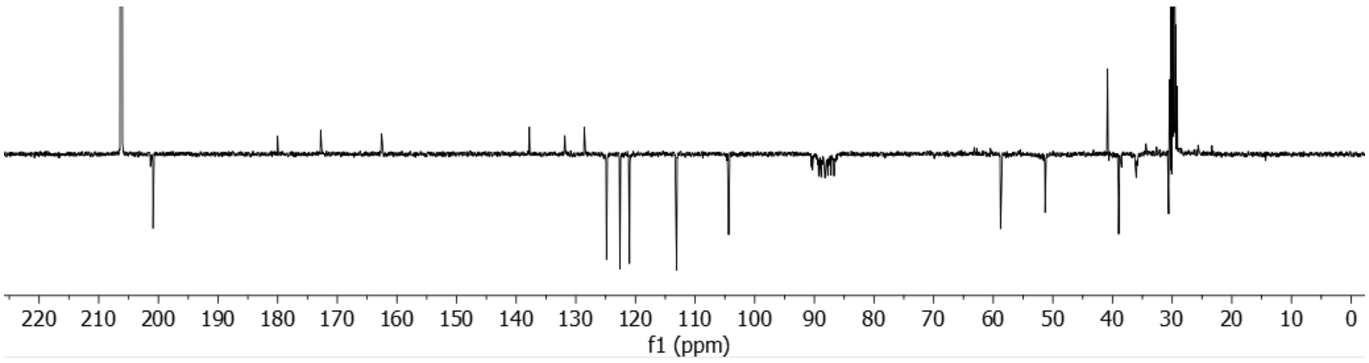

Ethyl (*S*,*E*)-4-((*S*)-2-((*tert*-butoxycarbonyl)amino)-3-((1*r*,2*R*,3*R*,4*R*,5*S*,6*S*)-2,3,4,5,6-pentafluorocyclohexyl)propanamido)-5-((*S*)-2-oxopyrrolidin-3-yl)pent-2-enoate (50)

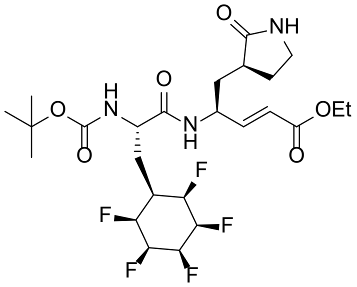

^{1}H NMR (400 MHz, Acetone-d_6)

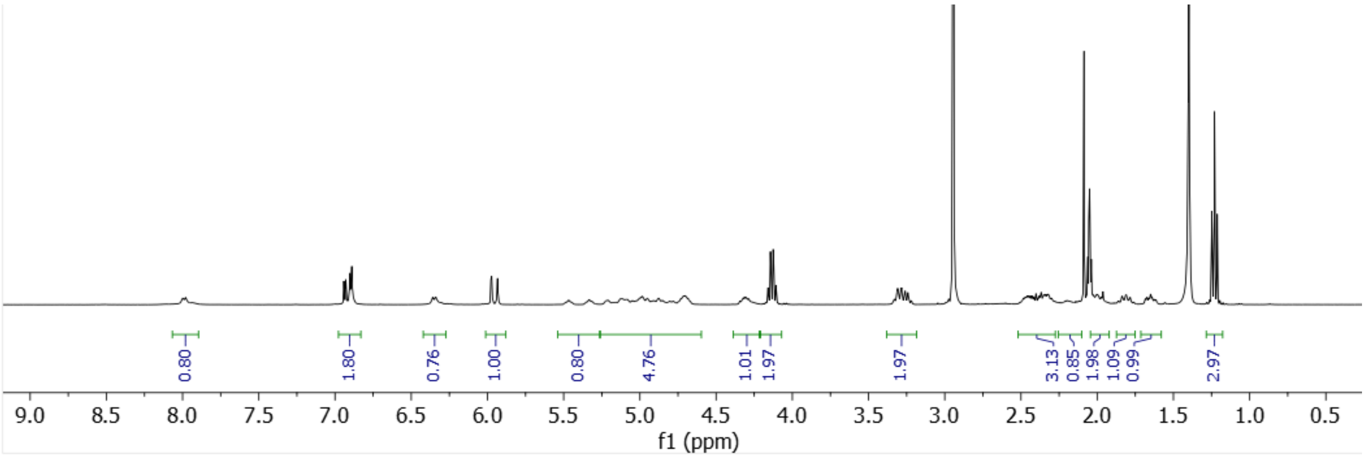

^{13}C NMR (126 MHz, Acetone-d_6)

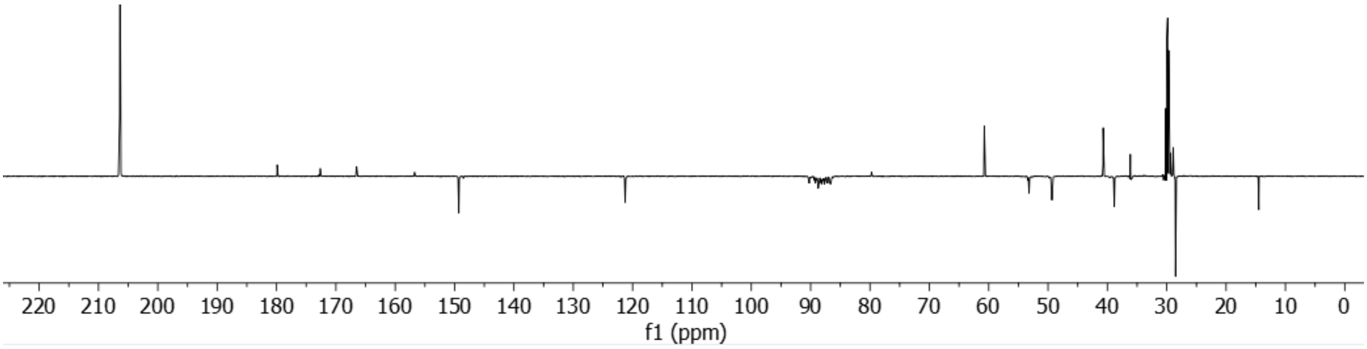

^{19}F NMR (376 MHz, Acetone-d_6)

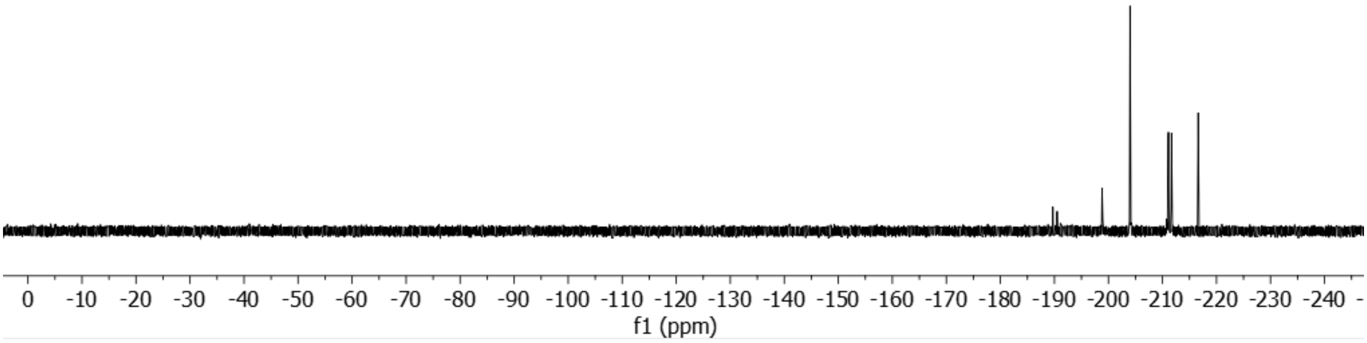

Ethyl (*S*,*E*)-4-((*S*)-2-cinnamamido-3-((1*r*,2*R*,3*R*,4*R*,5*S*,6*S*)-2,3,4,5,6-pentafluorocyclohexyl)propanamido)-5-((*S*)-2-oxopyrrolidin-3-yl)pent-2-enoate (52)

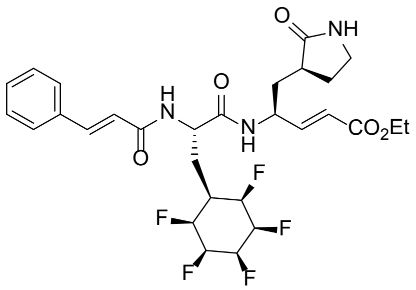

^{1}H NMR (400 MHz, DMSO-d_6)

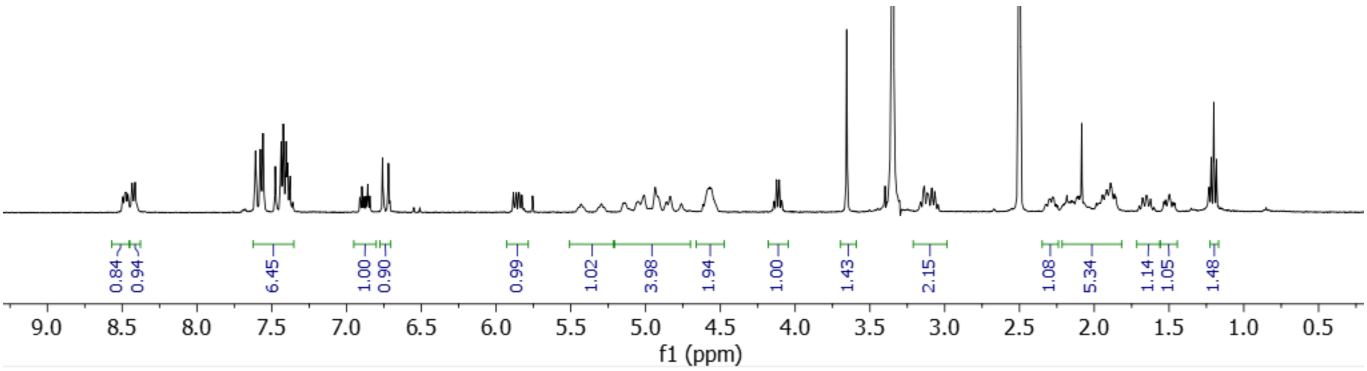

^{13}C NMR (126 MHz, DMSO-d_6)

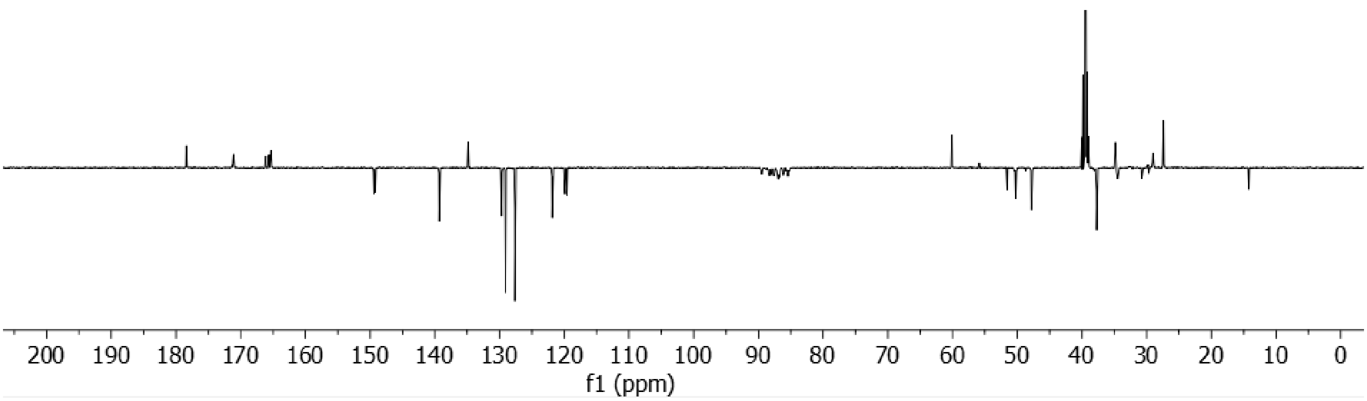

^{19}F NMR (376 MHz, DMSO-d_6)

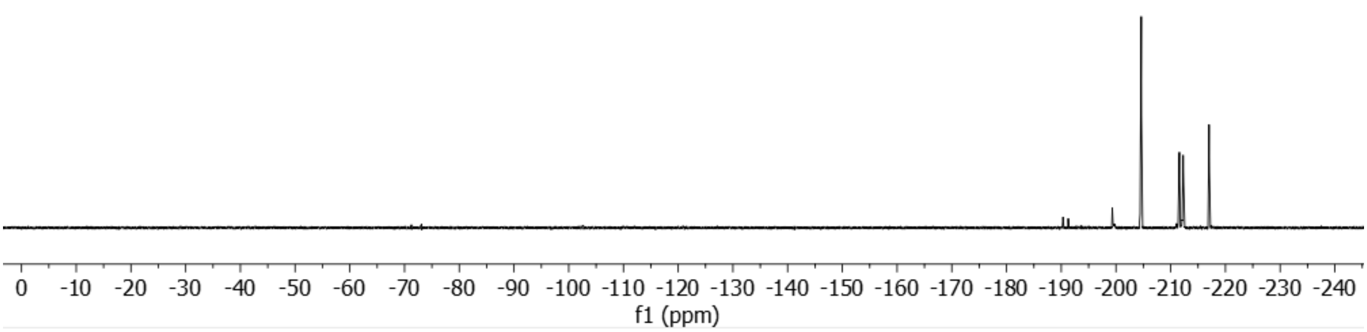

Ethyl (*S*,*E*)-4-((*S*)-2-(1H-indole-2-carboxamido)-3-((1*r*,2*R*,3*R*,4*R*,5*S*,6*S*)-2,3,4,5,6-pentafluorocyclohexyl)propanamido)-5-((*S*)-2-oxopyrrolidin-3-yl)pent-2-enoate (53)

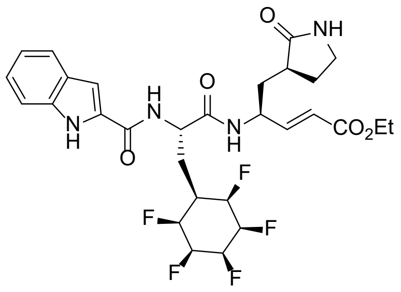

^{1}H NMR (400 MHz, Acetone-d_6)

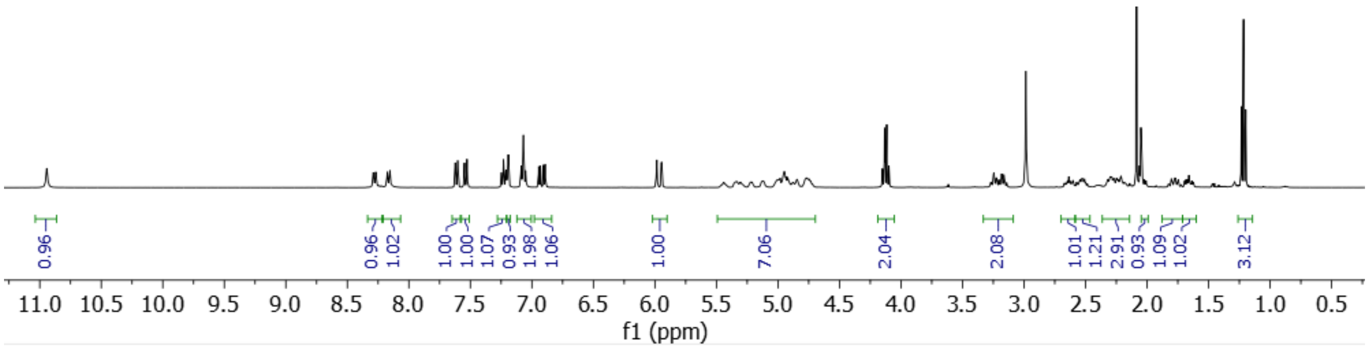

^{13}C NMR (126 MHz, Acetone-d_6)

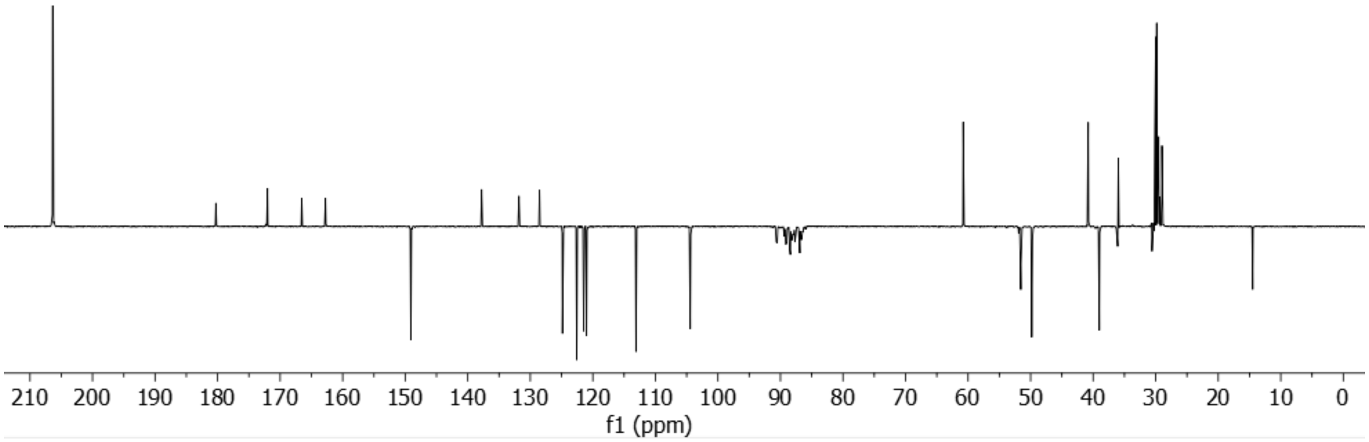

^{19}F NMR (377 MHz, Acetone-d_6)

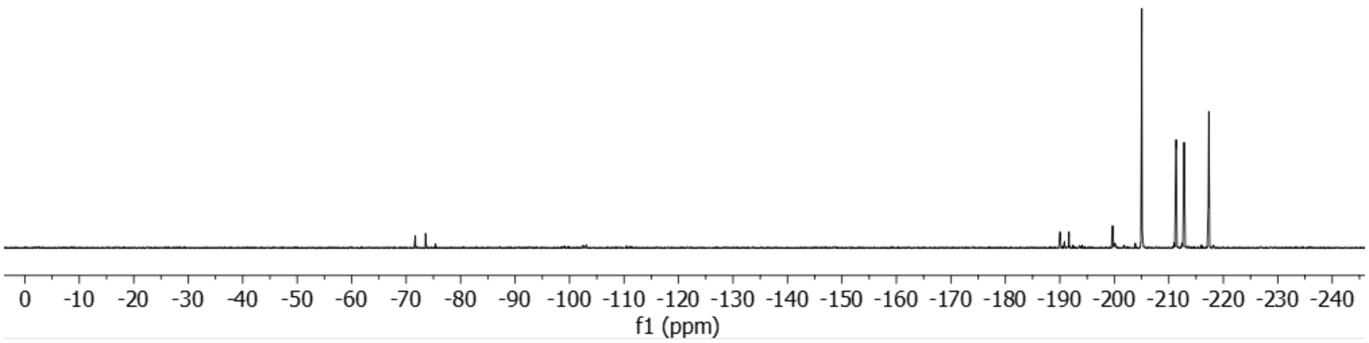

X-ray Crystallography Details

X-ray diffraction data for compounds **14**, **17**, **18**, **20**, **25**, **31** and **53** were collected at 173 K using a Rigaku MM-007HF High Brilliance RA generator/confocal optics with XtaLAB P100 diffractometer [Cu Kα radiation (λ = 1.54187 Å)]. Data for compounds **15**, **34·HCl** and **37** were collected at 125 or 173 K using a Rigaku MM-007HF High Brilliance RA generator/confocal optics with XtaLAB P200 diffractometer [Cu Kα radiation (λ = 1.54187 Å)]. Data for compound **19** were collected at 93 K using a Rigaku FR-X Ultrahigh Brilliance Microfocus RA generator/confocal optics with XtaLAB P200 diffractometer [Mo Kα radiation (λ = 0.71075 Å)]. Intensity data for all compounds were collected (CrystalClear[1]) using either both ω and φ steps or just ω steps, accumulating area detector images spanning at least a hemisphere of reciprocal space. Data for all compounds analysed were processed (including correction for Lorentz, polarization and absorption) using either CrystalClear or CrysAlisPro.[2] Structures were solved by direct (SIR2004[3] or SIR2011[4]), dual-space (SHELXT-2108/2[5]) or charge-flipping (Superflip[6]) methods, and refined by full-matrix least-squares against F^2 (SHELXL-2018/3[7]). Non-hydrogen atoms were refined anisotropically, and alkyl and aryl hydrogen atoms were refined using a riding model. Hydrogen atoms bound to heteroatoms were located from the difference Fourier map and refined isotropically subject to a distance restraint. All calculations were performed using either the CrystalStructure[8] or the Olex2[9] interface. Selected crystallographic data are presented in Table #. Deposition numbers 2098747-2098757 contains the supplementary crystallographic data for this paper. These data are provided free of charge by the joint Cambridge Crystallographic Data Centre and Fachinformationszentrum Karlsruhe Access Structures service www.ccdc.cam.ac.uk/structures.

References

[1] *CrystalClear-SM Expert* v2.1. Rigaku Americas, *The Woodlands, Texas, USA*, and Rigaku Corporation, *Tokyo, Japan*, 2015.

[2] *CrysAlisPro* (v1.171.38.46, v1.171.39.8d or v1.171.40.14a). Rigaku Oxford Diffraction, Rigaku Corporation, *Oxford, U.K.*, 2015-2018.

[3] Burla, M. C.; Caliandro, R.; Camalli, M.; Carrozzini, B.; Cascarano, G. L.; De Caro, L.; Giacovazzo, C.; Polidori, G.; Spagna, R. SIR2004: an improved tool for crystal structure determination and refinement. *J. Appl. Crystallogr.* **2005**, *38*, 381-388. doi: 10.1107/S002188980403225X

[4] Burla, M. C.; Caliandro, R.; Camalli, M.; Carrozzini, B.; Cascarano, G. L.; Giacovazzo, C.; Mallamo, M.; Mazzone, A.; Polidori, G.; Spagna, R. *J. Appl. Crystallogr.* **2012**, *45*, 357-361.

[5] Sheldrick, G. M. SHELXT – Integrated space-group and crystal structure determination. *Acta Crystallogr., Sect. A*. **2015**, *71*, 3-8. doi: 10.1107/S2053273314026370

[6] Palatinus, L. and Chapuis, G. SUPERFLIP – a computer program for the solution of crystal structures by charge flipping in arbitrary dimensions. *J. Appl. Crystallogr.* **2007**, *40*, 786-790. doi: 10.1107/S0021889807029238

[7] Sheldrick, G. M. Crystal structure refinement with SHELXL *Acta Crystallogr., Sect. C*. **2015**, *71*, 3-8. Doi: 10.1107/S2053229614024218

[8] *CrystalStructure* v4.3.0. Rigaku Americas, *The Woodlands, Texas, USA*, and Rigaku Corporation, *Tokyo, Japan*, 2018.

[9] Dolomanov, O. V.; Bourhis, L. J.; Gildea, R. J.; Howard, J. A. K.; Puschmann, H. OLEX2: a complete structure solution, refinement and analysis program. *J. Appl. Crystallogr*. **2009**, *42*, 339-341. doi: 10.1107/S0021889808042726

Table 1. Selected crystallographic data.

	**14**	**15**	**17**	**18**	**19**
empirical formula	$C_8H_{10}F_5N_3$	$C_9H_{12}F_5NO_2$	$C_9H_{11}F_5O_2$	$C_9H_{12}F_4O_2$	$C_8H_{11}F_5O$
fw	243.18	261.19	246.18	228.19	218.17
crystal description	Colourless needle	Colourless prism	Colourless needle	Colourless needle	Colourless plate
crystal size [mm^3]	0.24×0.03×0.02	0.10×0.04×0.01	0.23×0.04×0.01	0.42×0.02×0.01	0.25×0.20×0.01
Temperature [K]	173	173	173	173	93
space group	$P2_1/n$	$P2_1$	$P2_1/n$	$P2_1/c$	Cc
a [Å]	5.0634(2)	6.24684(15)	4.81236(16)	23.6341(18)	16.147(3)
b [Å]	16.4078(6)	5.73991(12)	10.4751(3)	4.9753(3)	19.497(3)
c [Å]	12.1176(5)	13.9736(4)	20.0409(6)	17.6312(13)	8.5209(13)
β [°]	99.401(4)	94.208(2)	92.150(3)	111.648(9)	98.109(4)
vol [Å]^3	993.20(7)	499.69(2)	1009.55(5)	1927.0(3)	2655.7(8)
Z	4	2	4	8	12
ρ (calc) [g/cm^3]	1.626	1.736	1.620	1.573	1.637
μ [mm^{-1}]	1.505	1.638	1.553	1.419	0.175
F(000)	496	268	504	944	1344
reflections collected	10147	5643	10577	20222	17319
independent reflections (R_{int})	1798 (0.0368)	1978 (0.0290)	1838 (0.0321)	3462 (0.0615)	4787 (0.0242)
parameters, restraints	146, 0	166, 4	146, 0	274, 0	391, 5
GOF on F^2	1.080	0.986	1.074	1.017	1.07
R_1 [$I > 2\sigma(I)$]	0.0429	0.0435	0.0465	0.0931	0.0217
wR_2 (all data)	0.1291	0.1302	0.1480	0.2327	0.0581
largest diff. peak/hole [e/Å^3]	0.30, -0.26	0.20, -0.38	0.31, -0.60	1.46, -0.36	0.19, -0.13

Table 1 con't. Selected crystallographic data.

	**20**	**25**	**31**	**34·HCl**	**37**	**53**
empirical formula	$C_{21}H_{25}F_5N_2O_2$	$C_{29}H_{31}F_5N_2O_3$	$C_{23}H_{21.5}F_5N_{3.5}$	$C_8H_{15}ClF_5NO$	$C_{15}H_{22}F_5NO_4$	$C_{29}H_{33}F_5N_4O_5$
fw	432.43	550.56	441.94	271.66	375.34	612.59
crystal description	Colourless prism	Colourless needle	Colourless needle	Colourless plate	Colourless plate	Colourless plate
crystal size [mm^3]	0.35×0.12×0.08	0.18×0.03×0.01	0.27×0.02×0.01	0.15×0.07×0.01	0.08×0.06×0.01	0.12×0.03×0.01
Temperature [K]	173	173	173	125	125	173
space group	$P2_1/n$	$P2_1/c$	$P2_1/n$	*Pbca*	$P2_12_12_1$	$P2_1$
a [Å]	11.2894(2)	13.9942(3)	24.7536(15)	7.0736(3)	5.09320(10)	10.8584(6)
b [Å]	9.5919(2)	21.1211(3)	5.7860(3)	8.7237(4)	15.7075(3)	4.9789(2)
c [Å]	19.4015(3)	9.76957(18)	30.2384(17)	36.5942(17)	22.0064(4)	26.3631(13)
α [°]						
β [°]	100.885(2)	108.414(2)	111.041(7)			100.023(5)
γ [°]						
vol [Å]^3	2063.13(7)	2739.76(9)	4042.1(4)	2258.15(18)	1760.54(6)	1403.51(12)
Z	4	4	8	8	4	2
ρ (calc) [g/cm^3]	1.392	1.335	1.452	1.598	1.416	1.450
μ [mm^{-1}]	1.037	0.931	1.025	3.521	1.193	1.045
F(000)	904	1152	1832	1120	784	640
reflections collected	20644	28847	40404	24201	21390	14682
independent reflections (R_{int})	3765 (0.0233)	4986 (0.0378)	7266 (0.0730)	2324 (0.0704)	3608 (0.0384)	4537 (0.0485)
parameters, restraints	277, 1	354, 0	708, 452	165, 5	234, 1	443, 128
GOF on F^2	1.041	1.035	1.092	1.101	1.047	1.022
R_1 [$I > 2\sigma(I)$]	0.0416	0.0415	0.0829	0.0786	0.0275	0.0412
wR_2 (all data)	0.1048	0.1270	0.2615	0.2284	0.0721	0.1131
largest diff. peak/hole [e/Å^3]	0.29, -0.37	0.28, -0.40	0.40, -0.32	1.44, -0.58	0.12, -0.17	0.19, -0.19
